# Development of a pain education resource for people with spinal cord injury

**DOI:** 10.3389/fpubh.2023.1197944

**Published:** 2023-07-24

**Authors:** Eva Widerström-Noga, Kimberly D. Anderson, Linda E. Robayo, Salome Perez, Alberto Martinez-Arizala, Lindsey Calle-Coule, Nicholas P. Cherup, Gabriel E. Fernandez

**Affiliations:** ^1^The Miami Project to Cure Paralysis, University of Miami Miller School of Medicine, Miami, FL, United States; ^2^Department of Neurological Surgery, University of Miami Miller School of Medicine, Miami, FL, United States; ^3^Department of Physical Medicine and Rehabilitation, MetroHealth Medical Center, Case Western Reserve University School of Medicine, Cleveland, OH, United States; ^4^Research Service, Bruce W. Carter Veterans Affairs Medical Center, Miami, FL, United States

**Keywords:** pain education, spinal cord injury, chronic pain, neuropathic pain, pain management

## Abstract

Many people with spinal cord injury (SCI) develop chronic pain, including neuropathic pain. Unfortunately, current treatments for this condition are often inadequate because SCI-associated neuropathic pain is complex and depends on various underlying mechanisms and contributing factors. Multimodal treatment strategies including but not limited to pharmacological treatments, physical rehabilitation, cognitive training, and pain education may be best suited to manage pain in this population. In this study, we developed an educational resource named the *SeePain* based on published pain literature, and direct stakeholder input, including people living with SCI and chronic pain, their significant others, and healthcare providers with expertise in SCI. The *SeePain* was then 1) systematically evaluated by stakeholders regarding its content, comprehensibility, and format using qualitative interviews and thematic analysis, and 2) modified based on their perspectives. The final resource is a comprehensive guide for people with SCI and their significant others or family members that is intended to increase health literacy and facilitate communication between SCI consumers and their healthcare providers. Future work will quantitatively validate the *SeePain* in a large SCI sample.

## 1. Introduction

Within the first year after spinal cord injury (SCI), up to 80% of the injured develop chronic pain, either nociceptive, neuropathic, or a combination of both (1–4). These pains often negatively impact quality of life by interfering with daily activities such as sleep, mood, social functioning, and physical function required for rehabilitation and vocational activities (2, 5–9). Treatments commonly used in other chronic pain populations are often less effective in reducing SCI-associated neuropathic pain. Thus, chronic neuropathic pain continues to be a very difficult problem and a top priority for improving quality of life in those living with SCI (10, 11). Because of the refractory nature of neuropathic pain in this population, the majority will continue to experience varying degrees of pain long after injury (2, 3, 12). Neuropathic pain is dependent on many different underlying mechanisms and contributing factors, some unique to SCI, thus, multimodal treatment strategies that incorporate adaptation and coping strategies (13) with pharmacological and non-pharmacological approaches may be best suited to manage this distinct pain condition (14).

Qualitative studies including people with SCI have revealed important perspectives regarding pain’s significant and negative impact on daily life (15–18). Some of the top facilitators to optimal pain management included having access to non-pharmacological treatment options and to better understand and communicate about pain. Other areas of interest included acquiring skills and knowledge regarding how to best self-manage and cope with pain to reduce the impact of pain on daily life (16, 17, 19, 20). Indeed, evidence suggests that pain catastrophizing and other cognitive strategies, such as a belief in one’s ability to control pain, substantially influence the severity of pain symptoms and development of comorbid psychological distress in those living with SCI (21, 22).

Internal locus of control includes the belief that one’s actions determine various health-related factors (23, 24). This concept has been associated not only with pain severity (25) and evoked pain (26) but also with long-term adjustment (27), coping mechanisms (28), psychological distress (29), physical disability (30), and satisfaction with daily activities (27). In addition, associations between lower pain intensity and greater levels of internal locus of control, adaptive coping, and less catastrophic thoughts after SCI have been suggested (31). Although locus of control is generally considered a personality trait, some data suggest that providing patient education can increase internal locus of control (32). Indeed, education integrated as part of a multimodal pain management program has been shown to reduce the severity of neuropathic pain after SCI (33).

Some research suggests that pain education should ideally be combined with other pain treatment strategies (34–36). A recent study by Mittinty et al. (36) examined the effects of pain education across people with heterogeneous chronic pain conditions. These investigators found that participants who reported an improved understanding of pain and self-management after pain education also reported lower pain intensity and positive treatment expectations (36). The results of this study correspond with SCI healthcare providers’ (HCPs) perceptions that effective provider-patient communication regarding therapeutic options and management goals is an important facilitator to better pain management (15). Consequently, pain management strategies incorporating pain education as an essential part of its framework may make chronic pain symptoms more manageable. Despite this apparent need, comprehensive consumer grounded education resources regarding chronic pain for those living with SCI are limited. Our research team has subsequently developed a unique educational resource titled the *SeePain*. The overarching goal of the *SeePain* is to provide relevant information regarding pain and pain management options, with the purpose of increasing health literacy for both patients and their significant others to facilitate better patient-provider communication. The *SeePain* was developed based on published pain literature, and direct stakeholder input, including people living with SCI and chronic pain, their significant others, and HCPs who specialize in treating SCI-related chronic pain.

The *SeePain* has not been systematically evaluated by primary stakeholders. Therefore, the primary purpose of the present study was to refine a preliminary version of the *SeePain* based on stakeholders’ input. The principal areas we sought feedback from stakeholders were: (1) Content, (2) Comprehensibility/Clarity, and (3) Format.

## 2. Methods

### 2.1. Participants

The study adhered to the principles of the Declaration of Helsinki and was approved by the Institutional Review Board of the University of Miami Miller School of Medicine.

Participants were recruited from The Miami Project to Cure Paralysis (Miami, FL) and from various SCI professional networks. Participants from the SCI pain group included men and women between 18 and 70 years of age who were fluent in English and who had experienced moderate to severe chronic neuropathic pain (CNP) (numeric rating scale [NRS] ≥ 4/10) for a minimum of a year. Participants were excluded if they reported a history of systemic illness (e.g., multiple sclerosis, rheumatoid arthritis, cancer). Significant others of individuals who experienced severe CNP after SCI were also recruited. For purposes of this study, a “significant other” was defined as an able-bodied person that is close to a person who meets the SCI pain group entry criteria but did not necessarily have to be a participant in this study. However, all of the significant others included in this study were close or immediate relatives of the participants from the SCI group. HCP participants were physicians who were required to have a minimum of 30% SCI patients in their clinical service. Generally, HCPs were between 35 and 59 years of age but were also eligible up to 75 years of age if they were actively practicing. HCP participants did not have any connections to the individuals with SCI or significant others participating in the study.

### 2.2. The *SeePain*

The preliminary educational tool, *SeePain*, was developed based on scientific literature and suggestions from people with SCI, their significant others/family members, and their HCPs. The *SeePain* contains basic information regarding pain after SCI in two separate modules. The first module focuses on pain in SCI, including pain classification, real cases, chronicity, and the psychosocial impact of pain. The second module focuses on different ways to manage pain, including self-management, non-pharmacological and pharmacological approaches. Both modules contain multiple quotes from all stakeholders that have been collected in previous qualitative studies to provide real-life context.

### 2.3. Research design

The study was designed as a two-phase mixed methods study, including qualitative interviews and a survey. The present paper presents the results from the qualitative interviews with people with SCI, their significant other/family members, and HCPs. Responses to the online survey were collected from a larger group of individuals with SCI who experience chronic pain and will be reported in a subsequent manuscript. Each person participated in 2–3 sessions. Most of the interviews were conducted via Zoom due to the COVID-19 pandemic.

#### 2.3.1. Session 1

After a phone screening to assess eligibility and provide information about the study, participants completed the informed consent process and the collection of demographic factors and medical history, pain, and psychosocial impact of pain (when appropriate) using interview-format questionnaires including the:

*International Spinal Cord Injury Pain Basic Data Set version 2 (ISCIPBDS)*. The ISCIPBDS is a brief instrument that contains questions about clinically relevant information concerning up to 3 separate pain problems during the last week, including pain interference with sleep, activities, and mood, pain intensity, and pain classification (37).

*The Multidimensional Pain Inventory Spinal Cord Injury version (MPI-SCI)*. The MPI-SCI (25) assesses the impact of chronic pain based on psychosocial factors. The MPI-SCI is based on a cognitive-behavioral perspective and was designed to assess the impact of chronic pain, responses by significant others to that pain, and emotional and physical adaptation to chronic pain (38). The answers are given on a numeric rating scale ranging from 0 to 6. The MPI-SCI’s psychometric properties have been investigated in the chronic pain SCI population and supports that the MPI-SCI is appropriate for use in this population (25).

*Difficulty in dealing with pain*. Participants rated overall, how hard is it for you to deal with your pain? (39) on an NRS from 0 (not hard at all) to 10 (extremely hard). Global ratings of difficulty in dealing with chronic pain and other consequences of injury have previously been used in the SCI population (40).

*The Spinal Cord Injury Pain Instrument (SCIPI)*: The SCIPI is a 4-item yes/no questionnaire used to screen for neuropathic pain in people with SCI (41). Each item endorsed for their worst pain is scored from 0 (negative) or 1 (positive) regarding sensations of electric shock, tingling or pins and needles, hot/burning or cold/freezing, and location of pain in an area with no sensation. The SCIPI was used to support neuropathic pain classification.

#### 2.3.2. Sessions 2 and 3 (qualitative interview)

Each participant completed one or two semi-structured Zoom or face-to-face interviews, with interview guides used to facilitate consistency across the interviews. The interview guide was based on a framework focused on the comprehensibility/clarity, content, and format of *SeePain*, and the analysis was based on grounded theory. The goal of this study was to ensure that the *SeePain* was firmly consumer grounded with optimal utility. All interviews were conducted jointly by Drs. Anderson and Widerström-Noga.

### 2.4. Data collection and analysis

The sample size was based on recommendations for grounded theory qualitative studies (42). All interviews were recorded and transcribed verbatim by an independent transcriber and checked for accuracy by study staff. The transcribed documents were analyzed in NVIVO by Drs. Anderson and Widerström-Noga. Qualitative content analysis was used to summarize and describe the information inherent in the data. Due to the narrow scope of our study, we reached saturation (no new themes emerged) at 12 SCI subjects, 10 SO subjects, and 8 SCI HCPs. Therefore, we completed our interviews with 15 SCI, 12 SO, and 10 HCP. The themes that emerged from Drs. Anderson and Widerström-Noga’s thematic analyses were discussed in study team meetings, which included an SCI pain neurologist (AM-A) and two SCI psychologists (SP, LC-C), and, if needed, themes were revised.

## 3. Results

### 3.1. Demographics, injury, and pain information

Demographic information for participants with SCI and their significant others is shown in Table 1. Participants in the SCI group included males (80%) and females (20%) with chronic neuropathic pain (mean age of 43.8 ± 9.2 years). The SO group participants included parents, spouses, and partners/companions of the SCI participants. HCP participants included physicians (7 females and 3 males) with extensive experience and expertise in SCI and board certification in Physical Medicine and Rehabilitation and/or SCI Medicine. Practice locations included Public Hospitals, the Veterans Affairs Healthcare System, and University-based Hospitals.

**Table 1:** Demographic information for SCI and SO participants.

	Spinal cord injury (SCI)	Significant other (SO)
	*N* = 15	*N* = 12*
Age (mean, SD)	43.8 (9.2)	47.9 (12.9)
Gender (n, %)		
Male	12 (80.0)	4 (33.3)
Female	3 (20.0)	8 (66.7)
Race/ethnicity (n, %)		
African American	4 (26.7)	3 (30.0)
Asian	0 (0)	0 (0)
Hispanic	6 (40.0)	6 (60.0)
White (Non-Hispanic)	3 (20.0)	1 (10.0)
Native American	0 (0)	0 (0)
Other	2 (13.3)	0 (0)
Marital status (n, %)		
Single	6 (40.0)	–
Married	7 (46.7)	–
Divorced/separated	2 (13.3)	–
Education (n, %)		
Pre-high school	1 (6.7)	0 (0)
High school	3 (20.0)	1 (10.0)
Some college	4 (26.7)	2 (20.0)
Bachelor’s degree	4 (26.7)	6 (60.0)
Trade school	0 (0)	1 (10.0)
Advanced degree	3 (20.0)	0 (0)
Other	0 (0)	0 (0)
Employment (n, %)		
Full-time	4 (26.7)	6 (60.0)
Part-time	0 (0)	0 (0)
Unemployed	8 (53.3)	3 (30.0)
Student	1 (6.7)	0 (0)
Retired	0 (0)	0 (0)
Homemaker	0 (0)	1 (10.0)
Other	2 (13.3)	0 (0)
Living arrangements (n, %)		
Living alone	1 (6.7)	–
Living with parents	5 (33.3)	–
Living with spouse	8 (53.3)	–
Living with roommate	0 (0)	–
Living with other relatives	1 (6.7)	–
Living in a nursing home	1 (6.7)	–

* Race/ethnicity, education, and employment were not reported by 2 SO participants.

Information regarding their injury, pain characteristics, medication, and psychosocial impact of pain for participants with SCI are included in Table 2. These participants sustained their SCI at the average age of 29.9 ± 12.2 years, and the average time since their injury was 14.0 ± 12.2 years. This group included 53.3% of participants with paraplegia and 46.7% with tetraplegia. The most common cause of injury was a motor vehicle accident (non-pedestrian). The onset of pain was within 1 year following their injury, consistent with previous literature for the development of neuropathic pain after SCI (3). More than 66% of SCI participants reported experiencing two or more pain problems, with an average intensity of 7.24 ± 1.6 (on a 0–10 NRS) for their worst pain problem during the last 7 days. Common medications reported by these participants included anti-convulsants, muscle relaxants, non-steroidal anti-inflammatory drugs, and opioids. The MPI indicated that the SCI participants experienced severe pain, moderate life interference, moderate affective distress, low activity levels despite moderate life control, and high perceived positive social support. Overall, this group presents a similar profile as the dysfunctional subgroup characterized in a previous study conducted in our laboratory (43).

**Table 2:** Injury and pain information for SCI participants.

	Spinal cord injury (SCI)
	*N* = 15
Age at injury (mean, SD)	29.9 (12.2)
Time since injury (mean, SD)	14.0 (12.2)
Type of injury (n, %)	
Paraplegia	8 (53.3)
Tetraplegia	7 (46.7)
Cause of injury (n, %)	
Sporting accident	1 (6.7)
Motor vehicle accident (Non-pedestrian)	8 (53.3)
Act of violence	3 (20.0)
Other	3 (20.0)
Pain onset (n, %)	
On date of injury	11 (73.3)
Months after injury (< 12 months)	3 (20.0)
Years after injury (≥ 12 months)	1 (6.7)
Number of pain problems (n, %)	
One	5 (33.3)
Two or more	10 (66.7)
Average pain intensity in the last 7 days (mean, SD)^a^	
Worst pain problem	7.24 (1.60)
Second worst pain problem	6.50 (2.20)
Third worst pain problem	6.00 (2.74)
Medication (n, %) *	
NSAIDs	2 (13.3)
Opioids	1 (6.7)
Anti-convulsants	5 (33.3)
Muscle relaxants	3 (20.0)
Anxiolytics	0 (0)
Cannabis	0 (0)
None	8 (53.3)
MPI Section 1 (mean, SD)^b^	
Pain severity	4.40 (1.61)
Life interference	3.53 (2.04)
Life control	3.33 (1.65)
Affective distress	3.07 (1.72)
Support	4.80 (1.39)
MPI Section 2 (mean, SD)^c^	
Negative responses	1.93 (2.67)
Solicitous responses	4.32 (1.75)
Distracting responses	3.35 (1.93)
MPI Section 3 (mean, SD)^c^	
General activities	1.62 (1.06)
Household activities	2.55 (1.96)
Outdoor activities	0.33 (0.67)
Activities away from home	2.30 (0.76)
Social activities	1.28 (0.86)
Pain impact	2.92 (2.65)
Household activities	3.45 (2.47)
Outdoor activities	2.00 (2.83)
Activities away from home	2.70 (2.56)
Social activities	3.53 (2.73)

* Some participants reported using more than one type of medication. ^a^ on a 0–10 scale, ^b^ on a 0–6 scale, ^c^ on a 0–6 scale.

### 3.2. Thematic analysis

The results of the thematic analysis are shown below. Examples of participants’ quotes are included for each theme (content, comprehensibility, and format). Additional quotes are provided in the Supplementary material.

#### 3.2.1. The value of *SeePain*

In general, the *SeePain* was well received. People living with SCI and their significant others expressed the value of the *SeePain* under a broad theme of useful and relevant information. Representative quotes include:

*“And it’s funny because I read the whole thing and most of the stuff, it helps you understand it clearly and it’s stuff that I learned on my own. And all the stuff that I learned on my own without nobody telling me, everything’s here.” “I know that it is not me anymore…, I guess its everybody, different areas maybe. But, it is a lot. I did not know the type of different pains, I thought it was just pain.” “….the quotes from people explaining the pain … I could understand it better.” “It was very informational because … I wish I would’ve gathered this when I first landed in the hospital and it would’ve helped me understand.”* “*I think it’s perfect that you include the healthcare providers. It’s important because sometimes…. all the quotations from people with spinal cord injury, we feel like they do not understand us, and sometimes they are the bad guys, and it’s not. They are in a really hard position.” “It was interesting… to hear the health care providers, the doctors…and it helps you see, or in my case have me see how to better try to communicate with them so they can understand.”*

HCPs also expressed the value of the *SeePain* in three sub-themes:

- Value in general: “*I thought it was an interesting approach in terms of mixing basic information with more scientific information with patient experience, interview responses. I think it’s helpful to have the three different perspectives.*”
- Value in clinical practice: “*I love this page and I actually personally took away something from it, because I like how it talks about acceptance, but accepting where something does not mean that you are giving in and that you are giving up.*”
- Value of the patient perspective: “*like there’s a lot more from the patient’s perspective because a lot of what we are doing is giving them our perspective. Occasionally, they might get to talk with another patient about what they are experiencing but for the most part it’s us telling them again and again what we are thinking.*”

#### 3.2.2. Suggested modifications of the *SeePain*

Participants with SCI, their significant others, and SCI HCPs offered extensive suggestions for the improvement of *SeePain*. These suggestions were organized in an *a priori* overarching framework concerning the *content*, *comprehensibility*, and *format* of the *SeePain*. All stakeholder suggestions were then organized into themes under each of these overarching categories. The interview transcripts from participants with SCI and their significant other were analyzed together, and the HCP transcripts separately. The subthemes were ordered in descending order based on the number of comments with representative quotes presented below. Additional quotes are provided as Supplementary materials.

##### 3.2.2.1. Content of the *SeePain*

The following represent the suggested modification provided by the SCI/Significant Others.

###### 3.2.2.1.1. Adding more relevant resources

*“It will be nice to have a health providers list that they manage this kind of chronic pain of people with spinal cord injury, because not all of them know how to.”*

###### 3.2.2.1.2. Adding more information about self-management and pain aggravation

*“We could add a section that says… what triggers the pain more? Like, for me, it’s…. sitting down too long, that’s number one.” “I used to go to the gym, it used to help a lot.”*

###### 3.2.2.1.3. Adding perspectives and support for significant others

*“We have to, the way they help us, we have to help them. So maybe somewhere a little more of our part, we have to do more to try to help them understand that and give them more emotional support too.”*

“*Because it’s like we are there for the person all the time, but it’s hard to have a conversation to a person who’s in that situation. So maybe what we can do so they feel less distressed or not in that much pain. How do we can help? And that is not in the booklet.”*

###### 3.2.2.1.4. Adding more information about cannabis and opioids

*“It (cannabis) takes away the… I do not know about the nerve pain a little bit but what is it? That hypersensitive skin that I have, it makes all that go away so my legs do not feel cold.”*

*“So, he was trying to back me off of those (opioids), and it worked, got me to a point where I do not even have to take it, except for those special occasions where my pain goes on and on, goes from the regular 4 to an 8, and I want to stop it before it comes a 10. That’s what I use it for.”*

*“I think there could be more on the positive reactions from people with pain to cannabis than what is here.”*

###### 3.2.2.1.5. Adding a word list with definitions and phonetics

*“Glossary would be the best thing because… you could pick it up quick on your phone but just to go back and reference….” Okay, that’s what that means. “And flip back. It’s so much quicker and easier. So… I think that would help out a lot.”*

###### 3.2.2.1.6. Adding more information about pain

*“For the real cases of pain, I can imagine there are a lot of examples. So if you want to go into details of cases of pain, it can be an ocean or you can make a whole book of it, if you want. I would say, maybe choose a couple of them to try to put into context what is the most cases.”*

###### 3.2.2.1.7. Adding more information about pain medication

*“When you look at this table, it’s good information, but it does not tell you the whole story. You could say, “There are more treatments.”*

*“I think it’s very helpful to make you understand that …. they can create addiction…. It helped me understand about an opioid, and that you got to be careful with it.”*

###### 3.2.2.1.8. Adding more information about the role of psychological factors

*“Yeah, it’s normal when you have an accident or whenever you have pain and you get told you are going to deal with it for the rest of your life. Yes, you just go over it mentally, some people struggle and they have suicidal thoughts.”*

###### 3.2.2.1.9. Adding more healthcare providers’ perspectives

*“The thing is, the healthcare providers I think it was very minimal, minimum, because it wasn’t very …they did not have, really, much to say about it.”*

The following represent the suggested modification provided by the HCPs.

###### 3.2.2.1.10. Adding other treatments used clinically but without strong evidence

*“Spinal cord stimulation, intrathecal pain pumps, effusions. I mean, there’s not … I think too much in there in terms of other procedural … there’s a rhizotomy mentioned, but it’s not talking about peripheral nerve blocks or axial injections, things like that.”*

###### 3.2.2.1.11. Mention that some treatments that may not be available to all patients

*“It’s like one of those things where research says it helps, and I think that’s true, but is it actually something that we can provide for our patient?”*

###### 3.2.2.1.12. Adding self-management and exercise adaptations

*“I immediately thought of what about the patients who cannot engage in exercise. It’s also something that you’ll probably want to include here. I’m thinking of that tetraplegic patient who would not be able to benefit.”*

###### 3.2.2.1.13. Other resources

*“The American Board of PMNR has a search function on their website where you can find somebody who’s board certified in the subspecialty of spinal cord injury.”*

###### 3.2.2.1.14. Adding potential medication side effects

*“I know if you look it up in your little app on your phone like I have, it lists hyponatremia as a potential side effect.”*

In summary, we addressed the comments regarding *content* by adding relevant resources, including free and paid resources and websites where physicians that are board certified in SCI are listed. Because self-management and avoiding triggers is an integral part of pain management mentioned by nearly all participants, we expanded information about self-management and aggravation and included common pain triggers. This section also included some text regarding common psychological comorbidities. Several participants with SCI suggested adding some content to provide support for their significant others or family members, and this was added by team members with expertise in SCI and psychological management. Participants also suggested adding additional information and perspectives on pain treatments including cannabis and opioids. The *SeePain* was expanded with respect to information about these pain medications and others, including common side effects. In addition, we updated the evidence-based treatment recommendations based on published guidelines, including those treatments where the evidence base was weak or absent but where the clinical experience may be positive and could be discussed with the provider. Similarly, we added the caveat that even though a treatment has a strong evidence base, it may not be available to all patients and providers. Based on the suggestions from several participants, we added a wordlist with definitions to help those with SCI and their significant others to better understand and communicate with their HCPs. Participants generally appreciated the different “voices” and perspectives regarding how pain impacted others with SCI living with neuropathic pain. However, they also stressed the importance of the HCPs’ perspectives to help them understand the physicians’ perspectives and better communicate about their pain. Information regarding perspectives from health care providers and how they view the treatment of pain was expanded based on this feedback.

##### 3.2.2.2. Comprehensibility of the *SeePain*

The following represent the suggested modification provided by the SCI/Significant Others.

###### 3.2.2.2.1. Clarification of figures, tables, and text

*“No, the pictures are good… but… it’s just like they seem kind of like hard to understand*.”

*“… I understand the colors, that part and then the numbers that go up, how it goes from zero to 100. But the thing I do not understand is the very good, rather good and sufficient and also the missing data.”*

###### 3.2.2.2.2. Clarification of concepts (pain, pain mechanisms)

*“I did try to understand it and I was … I learned something new, that there is a specific axon that carries the pain signals all the way to the brain through the spinal cord. That was something I did not know, and I was like, oh well, there it is.”*

*“Now that you are explaining it to me, I understand it. When I read it, I cannot tell … I did get that the pain is processed in different part of the brain, but I just got now that your brain, you feel the type of a pain, you feel how if it’s stabbing, or if it’s burning, or if it’s … I just learned that from you … now that you are mentioning it. Not from the module.”*

###### 3.2.2.2.3. Clarification of terminology

*“I would say its a lot of the medical, technical words that I could not understand. I am a scientist by training. I have BS in geology, in fact I did chemistry and physics and math and all that other stuff and I was overwhelmed.”*

The following represent the suggested modification provided by the HCPs.

###### 3.2.2.2.4. Adding clarification

*“Maybe we’ll just say, “In this module, we present multiple options, but based on your pain and your discussions with your healthcare provider and your insurance, collectively, you’ll make a decision about what’s best for you.”*

*“A little bit more explanation at the beginning, because otherwise, it’s like a 60 page document, which is probably going to be intimidating for people. If it’s kind of framed as there are different parts and find what parts are most useful to you, what parts you may skip over, that might be helpful.”*

###### 3.2.2.2.5. Opioids

*“There is the risk that someone could read it and say, “Oh, well, it looks to me like a lot of patients use opioids.” And according to this chart, that’s one of the highest zero to 10 relief comes from opioids. Why are you not giving me opioids?”*

###### 3.2.2.2.6. Modify or add figures

*“I think there was a slide that went through the different exercises to do. Maybe I just thought that it would be helpful if there were actual pictures of how to do this.”*

###### 3.2.2.2.7. Manage patient expectations

*“Think if you start with the expectation that, “Look, we will do our best, and we’ll keep trying as long as you want to, and there’s a lot of things we can try to do, but you need to understand that there is no easy magic bullet for this one, or for this type of pain, and if at some point along the way you decide this is good enough, I do not want to keep trying, other risks, other side effects, I’m happy to work with you wherever that point is.”*

Participants with SCI and their significant other suggested clarifications across figures, tables, and other text and making it clear from the beginning what to expect from the *SeePain*. Certain sections, especially the description of pain mechanisms and how the pain signal is generated and reaches the brain, were difficult to understand for some participants. We significantly clarified these sections and included analogies to better explain these concepts. We also modified those figures and diagrams to improve the readability and clarity of these sections. Related to this topic HCPs stressed the importance of discussing treatment options among the patient, their healthcare provider, and insurance carrier to ensure the feasibility of a particular treatment approach. Pictures describing the mechanisms of pain, non-pharmacological treatments, and average relief from treatments were not clear for some participants. Additionally, HCPs suggested that these figures needed to be clear concerning treatment effects. We modified figures and diagrams to improve the readability and clarity of these sections. Participants also requested Clarification of some terms. Therefore, we added definitions and phonetics to the word list at the end of module 2. HCPs stressed the importance of managing treatment expectations and trying many different approaches to find the best solution for the individual patient.

##### 3.2.2.3. Format of the *SeePain*

The following represent the suggested modification provided by the SCI/Significant Others.

###### 3.2.2.3.1. Format preferences

*“For me, personally….it would be a lot easier maybe watching that in a video…. but that’s my personal opinion… sometimes I have to read twice …to understand it.”*

*“The paper and the website are good. It’s, it’s always good to have both, in my opinion.”*

###### 3.2.2.3.2. Improve graphics

*“I did not understand very well this one. Like I understand the concept of the cells, like they change and they activate and all the factors and substance that are released can cause hypersensitivity. But the graphic, I’m not sure about it. I mean, think I can see it here but … I can understand it but when I see the picture … If I only can see the picture, I cannot understand.”*

###### 3.2.2.3.3. Change text or order of sections

*“Planning, learning, anticipating, big thing. As a matter of fact, I would’ve put this way earlier in the book than putting it there.”*

*“To give us some spaces, like a space between will it work and how it works. Leave like a space, a line there.”*

###### 3.2.2.3.4. Short version vs. long version

*“The books and the literature that you can own that people break down scientific… it’s all right. That’s for a person who wants to really be informed on the pain. Me, I just want to look for remedies. You know how sometimes on websites, if you hover over something, a little box will pop up with a definition?”*

The following represent the suggested modification provided by the HCPs.

###### 3.2.2.3.5. Modify text or layout

*“Trying to kind of parse them (quotations) down into the ones that really add more, and then even among those that add the most, maybe taking a shorter phrase or phrases within them.”*

*“Have something a summary at the end of each section, have a little summary set of bullet points.”*

###### 3.2.2.3.6. Format

*“As opposed to quoted written language, I think it would be more powerful if it was spoken.’*

*“I think, in general, having a written document like this that you could share when somebody’s in acute rehabilitation during one of these sort of education sessions, I think something like that would be fantastic. I think when someone has more chronic pain, unfortunately, probably a lot of them never get this information. So, still having that written or online thing would be good.”*

###### 3.2.2.3.7. Modify the order of sections

*“I had hoped to see it sooner… avoiding other triggers for pain. Because I’m an SCI doctor, I’m not just looking at their pain. I need to make sure that their bowel is actually well managed, that they do not have an active UTI, that their spasticity is actually controlled.”*

*“Move all of the other treatments, the psychological, psychosocial treatments to the front.”*

###### 3.2.2.3.8. Adding questions about pain and space for notes

*“I wonder if maybe at the back of the module, if we created a page with different things, like, you know, my pain is like where they can fill in information, you know, information about my pain, you know, what are my triggers? What are my, what do I, what are my goals? What do I want to use for non-pharmacologic options? What is my doctor, maybe, you know, like they fill it out with their doctor maybe as, what are my goals and what are the multiple strategies I want to use to manage my pain kind of thing.”*

###### 3.2.2.3.9. Short versus long version of *SeePain*

*“I think a shorter version would be helpful maybe even in the acute setting. Just so that they could have something to kind of look at in between sessions of therapy or whatever they are particularly doing.”*

*“Maybe trim it down for one version and keep the full version available.”*

All participants had varying preferences and valuable suggestions regarding the text and layout of the *SeePain*. While participants with SCI and their significant other primarily focused on the utility for their own use, the HCPs provided feedback based on a broader perspective incorporating their clinical expertise and the utility of education in SCI clinical settings. To address the suggestions of those with SCI and their significant others, substantial changes were made about color choices, pictures, and photos, and adding clarity to figures through better explanations. Both HCPs and those with SCI suggested changes to the order of sections; specifically, sections regarding how people planned their activities to better manage their pain, avoiding triggers of pain, and non-pharmacological treatment options were moved to earlier sections of the *SeePain*. With respect to the format and length of the *SeePain,* some felt that while it was important to have a comprehensive version of the *SeePain*, but that shorter versions could be useful, e.g., for those who had cognitive impairments, for early in-patient settings and for those who wanted to bring something simple to their doctor visits. There were also suggestions for brief points to emphasize important information. Some participants also suggested formats to be used in future versions of the *SeePain*. This included short videos of personal experiences, PowerPoint presentations, and different smartphone apps.

### 3.3. The revised *SeePain*

The revised module 1 contains 23 pages (see Supplementary Data Sheet 1), including a cover page, authors page, background, table of contents, main content, and space for notes. The main content is divided into 7 sections: what is pain?, pain after SCI, SCI classification, types of pain after SCI, real-cases of pain, chronic nature of pain after SCI, and impact of pain. In addition, module 1 contains multiple figures, diagrams and examples that help explain the concepts.

The revised module 2 contains 43 pages (see Supplementary Data Sheet 2), including a table of contents, main content, space for notes, resources, wordlist with definitions and references. This module is more extensive than module 1. The main content is divided into 11 sections: ways to manage your pain, avoiding triggers, evidence-based treatments, non-pharmacological approaches, recommended treatments, cannabinoids, thoughts about opioid medication, thoughts about pain medications, anxiety and depression, significant others/family/caregivers and final message. In addition, module 2 contains several pictures, diagrams and examples that help explain the concepts, as well as multiple resources.

## 4. Discussion

Chronic pain is a distressing comorbidity for people living with SCI (8, 44–46) and their caregivers (47–49), with many reporting significant reductions in quality of life as a direct result of their symptoms. Unfortunately, chronic pain associated with SCI is exceptionally difficult to treat, with clinicians stressing the significance and multidimensionality of both pathophysiological and psychosocial features (26, 50–52). Unique injury characteristics, the degree of social support, and other psychological concerns are all well known to differentially contribute to the capacity of people to manage their pain symptoms (46, 53, 54). Available evidence indicates that those with chronic pain greatly benefit from learning more about the nature of their pain and how to self-manage (33, 36). Increased health literacy and improved cognitions associated with pain and its treatments equip people living with pain and their significant others with pertinent information from which they may optimize communication with their healthcare provider (55). Additionally, educational material that emphasizes a range of pain management techniques provides individuals with a variety of alternatives that they may not have been exposed to or considered otherwise. We developed the *SeePain* to address this information gap and constructed an easily accessible resource entailing how chronic pain develops after SCI while also presenting various pain management strategies that have been advocated for in those with lived experience.

### 4.1. Pain management through pain education

Previous SCI studies have highlighted that many SCI consumers report difficulties in describing pain symptoms to their HCPs, with many questioning the expertise of their care team when treating neuropathic pain symptoms specifically (17, 56). For example, Widerstrom-Noga et al. (15) found that *poor healthcare provider communication about pain and available treatment options* were among the most significant barriers to consumers when discussing their treatment experiences. Moreover, individuals recounted that providers did not spend enough time nor provide enough information about their condition (17). This is especially concerning if patients rely on HCP recommendations for relief that is not adequately communicated. Such approaches may eventually lead to maladaptive coping strategies, negative cognitions, and unhealthy behaviors that ultimately exacerbate these symptoms in the long term. Indeed, these assumptions have been documented within a meaningful subset of this population with maladaptive coping skills and negative belief patterns significantly contributing to psychological distress and greater pain-related suffering (21, 57).

Patient education has subsequently been identified as a necessary and actionable step to improve chronic pain symptoms in a variety of clinical locals (58–60). For example, in a randomized controlled trial, Burton et al. (59) showed that a novel educational booklet that included evidence-based pain information and mitigation advice for those with low back pain led to significant improvements in individuals’ fear avoidance beliefs and pain-related disability. These individuals also retained these improvements at the one-year follow-up. Similarly, Moseley (58) showed that a single one-on-one pain education session, which included pain mechanisms and information concerning lumbar spine anatomy and physiology, led to appreciable improvements in pain related attitudes and beliefs, and resultant physical performance. The author suggested that merely providing information about the multidimensional nature of pain helps individuals to reconceptualize their symptoms through transference and reconfiguration of preconceived notions. The same benefit even seems to be the case when pain education is provided as a positive control. Notably, Thorn et al. (60) discovered that when compared to cognitive behavioral therapy (CBT) intervention, pain education led to small yet significant improvements in pain intensity and interference ratings at the posttest and 6-month follow-up (60). Compelling findings such as these suggest that information alone can help to improve certain dimensions of pain.

Pain education is often implemented as part of a larger multidisciplinary approach which includes psychological skills training and other pain mitigation strategies in those with SCI (33, 61, 62). This makes direct comparisons or evaluations for the overall efficacy of pain education difficult to assess in isolation. That said, Heutink et al. (61) showed that 10 weeks of educational training, which included cognitive behavioral therapy (CBT) strategies, improved self-reported ratings of pain intensity and pain-related disability in those with SCI. A similar pain management program was also found to elicit significant improvements across measures of mood and pain interference when compared to those allocated to a usual care group (62). While other between group measures in this study failed to reach statistical significance, the authors identified additional improvements in pain catastrophizing, ratings of usual pain intensity, and measures of anxiety in those assigned to the pain management group alone. Conversely, Budh et al. (33) showed that a similar pain education and CBT program, failed to improve self-reported measures of pain intensity and pain unpleasantness, but lead to selective improvements across measures of anxiety and depression. One potential reason for the disparity across studies may be due to the nature of neuropathic pain as a multifaceted condition that often bears variable health-related repercussions (4, 5, 63).

It also seems to be the case that some may benefit from standardized treatment approaches, while others will require approaches contingent on their idiosyncratic symptoms. From such considerations, psychosocial variables, including pain-related beliefs or cognitions and the implementation of various coping strategies, do appear to impact how individuals adjust to living with their pain (22, 64). Catastrophic thinking and pain-related beliefs, such as “pain signals mean damage,” are known to be associated with increases in pain interference and poorer mental health (64). Indeed, those with chronic pain and externally perceived health-related locus of control have been identified as suffering from greater psychological distress and feelings of helplessness when viewing their own pain management skills (65). Supporting this claim, Conant (66) found an inverse relationship between internal locus of control measures and reported pain perceptions among those with chronic SCI, stating that those with less confidence in their ability to influence their health would likely benefit from interventions that develop awareness over certain aspects of their condition that they may have some control. It has also been proposed that educating significant others about SCI-related complications (including chronic pain) may improve quality of life and help to mitigate collateral health consequences in those taking care of their significant others full time (67). Information outlining various pain scenarios and effective self-management techniques can thus do little-to-no additional harm and bolster any existing support system that those living with SCI may require. An additional caveat that emerged from the HCP interviews in the current study focused on the need to temper patient expectations. For example, individual’s living with SCI associated neuropathic pain have been known to be exceedingly optimistic about the physician’s abilities to treat their condition. As a consequence, providers repeatedly encouraged the addition of information about setting expectations for patient recovery following SCI.

### 4.2. Future recommendations

Considering that few studies have examined the effectiveness of education as a means to reduce neuropathic pain symptoms in those with SCI, future research should examine the usefulness of diverse education materials in this population. For example, an online interactive tool may be better suited for those who prefer more participatory-based learning. That said, the medium and other presentation considerations may impact how the included information is engaged with and ultimately understood. There may also be latent symptom characteristics such as pain intensity or pain unpleasantness that impose a ceiling effect on the value of educational tools. Therefore, the effectiveness of such resources should be partitioned based on patient needs. Researchers should also consider the length of such resources to better tailor the volume of information to the educational level of the individual. Future large-scale studies are required to better understand these potentially important concerns. We also believe that the *SeePain* will provide an excellent complement to other SCI pain related resources such as the ones developed by the Model Systems Knowledge Translation Center.^1^ More importantly, because the *SeePain* was developed using scientific evidence integrated with the specific needs and perspectives voiced by individuals with SCI and chronic pain, their significant others, and healthcare providers, it represents a unique and personalized reference for those dealing with is complex condition.

### 4.3. Limitations

Even though we collected information from those with SCI, their significant others, and HCP perspectives regarding the content, relevance, comprehensiveness, and format of the *SeePain*, it is possible that we did not capture all possible input due to methodological constraints. Especially among people with SCI living with chronic pain who do not have a significant other or other support sources. Another limitation of the *SeePain* is that it will need to be updated continuously based on the input from stakeholders, as evidence-based therapies and overall treatment approaches change over time. Additionally, we acknowledge that the two modules are rather lengthy and not everybody will be interested in every section; however, it was important to receive feedback on the full version before creating alternative formats. We have also not tested the validity or effectiveness of the *SeePain* to improve health literacy or quality of life in this population. These easily testable hypotheses may be explored in future studies.

## 5. Conclusion

The *SeePain* represents a unique pain education resource specifically designed through a structured process that incorporated feedback provided by SCI consumers living with neuropathic pain, their significant others, and pain clinicians with expertise in the field of SCI rehabilitation. This resource thus serves as a brief and easily understandable bridge between primary stakeholders living and treating SCI-associated neuropathic pain. We anticipate that making such information available will help to mitigate perceived barriers and facilitate good faith communication between SCI consumers and their HCP.

## Data availability statement

The raw data supporting the conclusions of this article will be made available by the authors, without undue reservation.

## Ethics statement

The studies involving human participants were reviewed and approved by the Institutional Review Board of the University of Miami Miller School of Medicine. The patients/participants provided their written informed consent to participate in this study. Written informed consent was obtained from the individual(s), and minor(s)’ legal guardian/next of kin, for the publication of any potentially identifiable images or data included in this article.

## Author contributions

EW-N and KDA designed the study, conducted the qualitative interviews, and thematic analysis. EW-N, KDA, LER, SP, AM-A, LC-C, NC, and GF conducted the *SeePain* revisions and edits. EW-N, LER, NC, and GF prepared the manuscript draft. All authors substantially contributed to the interpretation of data and manuscript revision.

## Funding

This work was supported by the Craig H. Nielsen Foundation (592408) and The Miami Project to Cure Paralysis. However, these institutions were not involved in the study design, collection, analysis, and interpretation of data, neither in writing the report nor in the decision to submit the article for publication.

## Conflict of interest

The authors declare that the research was conducted in the absence of any commercial or financial relationships that could be construed as a potential conflict of interest.

## Publisher’s note

All claims expressed in this article are solely those of the authors and do not necessarily represent those of their affiliated organizations, or those of the publisher, the editors and the reviewers. Any product that may be evaluated in this article, or claim that may be made by its manufacturer, is not guaranteed or endorsed by the publisher.

## Footnotes

^1^ msktc.org
