# Supplementary material for: Development of a pain education resource for people with spinal cord injury
Source: Front Public Health. 2023 Jul 24;11:1197944. doi: 10.3389/fpubh.2023.1197944 (PMC10406314; doi:10.3389/fpubh.2023.1197944)
Supplement: Supplementary file 1 [file Table_1.DOCX]

Supplementary Material

Article Title

**Widerström-Noga E*, Anderson KD, Robayo LE, Perez S, Martinez-Arizala A, Calle-Coule L, Cherup NP & Fernandez G**

*** Correspondence:** Corresponding Author: ewiderstrom-noga@med.miami.edu

# Supplementary Table 1. SCI/SO participants

| **Code** | | **Quotes** | | **Files** | | **Ref** |
| --- | --- | --- | --- | --- | --- | --- |
| **Content** | | | | | | |
| *Adding a word list with definitions and phonetics* | | *Especially for the medical terms, you know, it's good to add what they mean so people knows, because if somebody was dealing with it, they might under- ... Even me, I deal, I deal with it. There's a lot of terms that I didn't quite understand at first, you know?* | | 22 | | 27 |
| *Adding more information about pain medication* | | *I like it, especially the how it works. I like that. I like the explanations on that, what it attacks, where it ... One other thing is maybe what organ it affects when you're taking it. So, some pills affect your liver if you take too much of it. Some of it, your kidneys, so on and so forth. I think that would help too there.* | | 14 | | 17 |
| *Adding more healthcare providers perspectives.* | | *“The thing is, the healthcare providers I think it was very minimal, minimum, because it wasn't very ...they didn't have, really, much to say about it”* [Interviewer: “*Do you think we need more?*”] *“I think so, because we rely on them, you know?”* | | 5 | | 6 |
| *Adding more information about self-management and pain aggravation* | | *“You didn't mention in the document if some pains are related to some postures on the body, and I don't know if that is important to mention it, because for me particularly, I'm quadriplegic. I have some sensation, and one of my pains are in the butt, and it's because I stay in my wheelchair for a long time all day, and the pain's telling me that you have to rest.”*  *“we could add a section that says, okay what makes you feel more, but like, what triggers the pain more?”* | | 22 | | 31 |
| *Adding more information about cannabis and opioids* | | *“That they should understand that if the tolerance goes high and you can't get it, what are you going to do about it? Are you going to result to go to the street and take the chance of dying? Do you phone a friend that you may think that has it? I mean, what do you do? I mean, maybe you should say what can you do and then bam. Call your doctor immediately.”*  *“I think she hit on it earlier, an updated information on the marijuana piece. Because it's out there by itself, and the way it's presented, it's out there by itself. But it doesn't drill down, or have the reminder table or anything else in there.”* | | 22 | | 31 |
| *Adding more information about pain* | | *“For the real cases of pain, I can imagine there are a lot of examples. So if you want to go into details of cases of pain, it can be an ocean or you can make a whole book of it, if you want. I would say, maybe choose a couple of them to try to put into context what is the most cases. In that case, also in the real cases of pain, I would like to say also, if you found useful information that that patients can also use to share to other people, even in the words, how to put these cases. Because, in these real cases is where you see how it's actually happening. I wouldn't mind if you do more real cases.*  *“And, the below-level neuropathic pain, which is, yeah. It increases. And I think it's important for people to know too. That after one year you don't want to see a further increase. Because if you see a further increase there maybe other reasons for that pain. So usually it's within the first year, it devolves. It may change in milder or more severe over the years. But it shouldn't develop new pain in that category after a year.”* | | 16 | | 22 |
| *Adding more information about the role of psychological factors* | | *“Because like I say, like me, when I'm in pain I become so like, oh come on. Sometimes I tell my kids…Mommy is in pain, and try not to be not so close to mommy because mommy is arr, arr, arr, arr, arr, grumpy…. But at the same time, I talk to myself, I'm my own psychology. I say, "Okay, me crying, I won't resolve no problems. So move away. Okay, you in pain. But you have two kids. You have two kids you have to raise." You get it? So like it's depending on people mentality.”* | | 9 | | 9 |
| *Adding more relevant resources* | | *“I think actually that a good resource would just be good, and it's not an article. It would be to find a group. Tell them to find a group that you have down here at pain management networks. But yeah, networking with a group of people that can sympathize with what you're going through.”*  *“Something will be nice to have it there, just to know where to go if you have chronic pain. Here are the best.”* | | 35 | | 48 |
| *Adding perspectives and support for significant others* | | *“I think that would be helpful because just as the person with the injury isn't they prepared, neither is the caregiver or the person in the home. With the injured person and the impact is on all of us, everyone involved. So yeah, I do think that would be helpful.”*  *“How we can support our family members with this type of injury. What is it that we could do? Because sometimes my brother is in pain and I don't know what to do and I want to do more. But that way, if I understand more, then maybe I could do more.”* | | 21 | | 32 |
| *Useful and relevant information* | | *“The pain, it's a very personal, um, experience, and when you get to, to have other people reaction, i-i-it's better. You relate to it better because the, what the people say really, it's, it's like, yeah, it's true, you're not lying because I'm, I'm, uh, finding myself in the same predicament.”*  *“I saw a few question that I have myself all the time like its only me the one who feels this. I know that it is not me anymore. Just me, I guess its everybody, different areas maybe. But, it is a lot. I didn't know the type of different pains, I thought it was just pain.”*  *“It was very informational because ... See, one thing I'm going to tell you guys is I wish I would've gathered this when I first landed in the hospital and it would've helped me understand. Because all the doctors would come in there and be like, "See this? See that?" And, "Oh this, you're this." And I'll be like, "Huh?" So I never knew nothing while I was in the hospital because they just kept saying all this stuff and I'm like, "What is all that?" But then this helped me understand a lot of it, what they've been talking about.”* | | 61 | | 311 |
| **Comprehensibility** | | | | | | |
| *Clarification of concepts (pain, pain mechanisms)* | | *“Like, in that little picture that shows the normal inhibition and reduce, I will say like just explain like... You know, when people don't know about medical terminology and things like that, it's good to give them like a little... How can I say? Like, if you have two apple and you eat one, you got one left. You get it? Like show with examples.”*  *“Now that you're explaining it to me, I understand it. When I read it, I can't tell ... I did get that the pain is processed in different part of the brain, but I just got now that your brain, you feel the type of a pain, you feel how if it's stabbing, or if it's burning, or if it's ... I just learned that from you … now that you're mentioning it. Not from the module.”* | | 41 | | 84 |
| *Clarification of figures, tables and text* | | *“I didn't understand very well this one. Like I understand the concept of the cells, like they change and they activate and all the factors and substance that are released can cause hypersensitivity. But the graphic, I'm not sure about it. I mean, think I can see it here but ... I can understand it but when I see the picture ... If I only can see the picture, I cannot understand.”*  *“The figure I had to analyze more. I understood what it was trying to say. The figure is not very helpful. It's not very attractive. It's not very ... I don't know how you guys ... It's not easy to understand.”*  *“You could still leave the name, but then draw a arrow that says, "Okay, this is going to impact your legs," or "This will impact your stomach," or something like that, that shows the impact areas that are associated with these names. It's still good to know the technical terms, but you just have to know what it's linked to.”* | | 51 | | 124 |
| *Clarification of terminology* | | *“Especially for the medical terms, you know, it's good to add what they mean so people knows, because if somebody was dealing with it, they might under- ... Even me, I deal, I deal with it. There's a lot of terms that I didn't quite understand at first, you know?”*  *“I was getting a little frustrated with some of the terminology because I was like, "I don't understand this."* | | 40 | | 67 |
| *Understandable* | | *“I think that they're talking their truth….. I may not feel all of them like some of them because it's not maybe the same knee injury, not the same level, not the same type, but I can see how they're ... Everything's true... And it's understandable. They're trying to explain the experience that they're going through.”*  *“I found to include the patients’ thoughts and whatever they felt at a certain moments with how they describe the pain and how they handle it. It was astonishing. It was extremely, extremely helpful to understand. I mean, things that we don't actually.. don't understand the terms and how the pain actually works. And it was very easy to understand, and it was extremely helpful and very interesting”* | | 32 | | 93 |
| **Format** | | | | | | |
| *Change text or order of sections* | *“Planning, learning, anticipating, big thing. As a matter of fact, I would've put this way earlier in the book than putting it there. I would've put it way earlier in module two then I would've put it later.”*  *“I think we have here some value information about depression and suicidal thoughts that can be more segmented or can be presented in a more dynamic way. Now, just a big paragraph that sometimes people when seeing such a huge paragraph, kind of read between lines and skip some part of it, and it's very important, all the feelings and all the things you mentioned in there.”*  *“I mean, or probably put the first paragraph, then the table, and then about 60% of people with spinal cord injury. So you may want to try to accommodate a table in one page, or accelerate.”* | | 23 | | 47 | |
| *Format preferences* | *“For me, personally, because I just said it, I don't read a lot, for me, yeah, it would be a lot easier maybe watching that in a video. Yeah, but that's my personal opinion, yeah. I mean, sometimes I have to read twice something to understand it, and if you're telling me I don't have to get it twice, you know what I mean?”*  *“So if it's online, somebody goes to look at it and they click on section D, and it just brings them straight to page 20 instead of having to scroll through the first part of the document. I don't know.”*  *“Definitely paper in hands. For work, I'm on the internet all day. So if I can stay away from a computer to learn something, or if I can read it in bed later, that's better for me.”*  *“And someone that is in traction and can't read a book, maybe able to listen to some of this and be able to understand what's happening with their body, before they're able to start moving again.”* | | 50 | | 98 | |
| *Improve graphics* | *“The one thing I did question was its difference in colors, because I interpret the color red as pain. So I'm seeing blue, and that would be to me a more cooling feeling, more soothing. Like green, it doesn't look like pain to me. Red is pain.”*  *"It didn't make sense to me at first, but then I had to really analyze it, get into it, and then I could understand what it was trying to say. But at first it didn't make sense to me. I expected to see the treatments, where it says very good, good, rather good, insufficient, I expected to see the treatments in that area, and then the bars to be the good or not very good or.”* | | 36 | | 73 | |
| *Short version vs long version* | *“Maybe you could make a more condensed version of this that would be more high level for people that are into reading a lot.”*  *“Probably in a brochure, you can skip the quotes, and maybe shorter. Like the quantity of the ... I mean for me, it's great, but thinking of ... I have been working ... I work for a nonprofit, and we think about all the time on things that are the best for people to understand and has to be shorter and it has to be this way and this, so thinking of that, probably 20 pages will be too much.”* | | 10 | | 16 | |

# Supplementary Table 2. HCP participants

| **Code** | **Quotes** | **Files** | **Ref** |
| --- | --- | --- | --- |
| **Comprehensibility** | | | |
| **Adding clarification** | *“pain that's associated with new fevers, infectious signs, or pain that's associated with a change in your neurologic function, …. maybe just a small section on times to definitely have a conversation with your provider.”*  *“I think it is a mistake to call placebo fake treatment”.*  *"Because the pain experience is different, not every medication works for every patient. And even if it's the 'best' and it doesn't work for you, it's likely that something else will."*  *Since this is a patient education thing for both the Lyrica and the Gabapentin, that I would put in, and I might put it in bold letters that, "If you're on this drug, you cannot stop abruptly because both of those drugs have a high risk of seizures if you stop them abruptly."*  *“One important part of managing your pain is to figure out what can make your pain worse." And then you can just say, "These factors do not have the same effect on all people with SCI."*  *“these are quotations from their personal experiences…just… at the very beginning, “items in purple font represent people's personal experiences. Yours may be different.”* | 8 | 51 |
| **Opioids** | *“like tolerance develops over time, it did for that patient, it's not always true. What's true for one person may not be true for everybody.”*  *“Especially those that go to an emergency for example, ER doctor, he will give you tons of opioids. Are you educating that doctor to tell him, you know what, this is what I know about my pain. This is one is gonna work or, or you just take it, you know?”*  *"Do this, do that, plus that, plus that, plus that, plus that, everything." You can get sometimes 10% improvement here, 10% here, 10% there, and then makes 40% improvement, which is amazing.”* | 9 | 29 |
| **Modify or add figures.** | *“I think for nociceptive pain, just a small thing, the example using kind of like a burn as the example of pain, I just sort of thought maybe something more relevant to somebody with spinal cord injury, like shoulder injury or something***.”**  *“And maybe we need to make a distinction between acute and chronic pain because obviously, that's completely different. Even if it's nociceptive pain. So I think in this, we'll probably focus more on the chronic pains rather than... Even though the picture here is showing an acute nociceptive pain. So maybe we need to make a distinction there because it's obviously not the same thing.”* | 6 | 15 |
| **Manage patient expectations.** | *“I almost wonder if there could be some sort of summary page about like, almost questions to ask your provider about your pain, or maybe a guide, a discussion guide, how to discuss your pain with your provider in terms of having them really have an explicit conversation with them about what are your expectations as my provider, or what should I expect about what's going to happen with this pain, or how we're going to treat this pain, or where do you think it's coming from?”* | 3 | 5 |
| **Content** | | | |
| **Mentioning other treatments** | **used clinically but without strong evidence**  *“Sometimes we use medications in combination too, so if a patient's getting maybe 80% relief from Lyrica but we're not quite there, then sometimes I might overlay it with the Nortriptyline or something else that will kind of take that last little edge off for them.”*  *“Just getting in for example a standing frame or tilt tables or a FES spike, so I don't know if that's something you want to incorporate...”* | 4 | 12 |
| **Mention that some treatments may not be available.** | **Have evidence but may not be available to every patient.**  *“I have never in my practice, I've read of course about transcranial stimulation and how it can help with spasticity as well and neuropathic pain, but I don't see this as something that's commonly available or done.”*  *“If I were to present this to someone, depending on the patient, I might really want to give it to them and say, "Okay, would you want to review this? Let's talk about it afterwards if you have specific questions," or go over it with them …just to be able to kind of guide them through”* | 6 | 11 |
| **Adding self-management and exercise adaptations** | *“I'm thinking, because just now, and we've looked at this for so long, but a lot of people told us about changing positions and stretching, maybe we'll put that as exercise options here for people that may not be able to do much weight lifting…”* | 3 | 7 |
| **Other resources** | *“I normally send them to the University of Alabama EatRight program. Have you seen this? The EatRight program have a 12-week program for weight management, where one of the two or three weeks they show spinal cord injury patients performing those exercises. I think this would be very interesting, very educational. One thing is, describe the exercise. Another thing is also, show the exercise, the one performing the exercise.”* | 2 | 3 |
| **Adding medication side effects** | *“rather than saying these are the side effects, maybe saying these are potential side effects because you wouldn't want to give people the impression that they always have side effects”* | 2 | 2 |
| **Format** | | | |
| **Modify text or layout** | ***“****I thought it was a little bit wordy, the background information.”*  *“I think another thing for me was just the layout of things, like we said before, not only this page, just a lot of them, it was just a bit overwhelming to see at first. So even if we need to separate it at another page, we don't have to try to squeeze everything into one page. So it's less overwhelming.”*  *“So…. first page "Why is pain education important?" I might go so cheesy as to take out the first two things, "Makes it easier to manage." And, "Know what you can do to manage your pain." And just say something like, "Knowledge is power. Education makes it easier to manage the pain."*  *“one of my suggestions, was you have something a summary at the end of each section”* | 10 | 66 |
| **Format** | *“I think this would probably be easy to change into presentation slides. That could then either be voiceover as a video presentation or used in kind of a community.”*  *“I think that the quotes are powerful, but because they are clearly quoted verbal language, as opposed to quoted written language, I think it would be more powerful if it was spoken.”* | 5 | 11 |
| **Modify order of sections** | *“the avoiding triggers page, and I almost wondered whether it should have been in the first module about the causes of pain.”*  *“the purpose of SeePain is to help people better understand. I thought maybe that should have been written from the beginning, this is the purpose of it, not in the middle of the page there.”*  *“there's a wealth of psychosocial ways to modulate your pain. And those occur in parallel with more traditional pharmacological and interventions like TENS and things like that. And I would actually move those to the beginning of this, to talk about those things, which everybody can do, and have been shown to be very, very helpful in modulating the pain.”* | 4 | 11 |
| **Adding questions about pain and space for notes** | *“if there could be …questions to ask your provider about your pain, or maybe a guide, a discussion guide, how to discuss your pain with your provider in terms of having them really have an explicit conversation with them about what are your expectations as my provider, or what should I expect about what's going to happen with this pain, or how we're going to treat this pain, or where do you think it's coming from?”* | 3 | 6 |
| **Shorter version** | **Shorter version for e.g., cognitively impaired, older, lower education level**  *“a condensed version of the first section and then more of a menu kind of summary of the interventions. And then if they want to learn more about what's in this kind of ... what's the evidence of pregabalin or what have you, they can go to the longer version.”*  *“I think it would be helpful to maybe... A bullet point at some part of this to... So for people who scan, because it could be intimidating. It certainly was not what I was expecting. It's very well-written, thought out and it's long. And I thought it was going to be something quick for, I guess, our consumption, the way we look at things these days.”* | 3 | 5 |

For the SeePain:

If it's not causing problems, then I mean, I'll offer people alternatives and say you could try this if you wanted to and see if you could decrease the amount of opiates you're taking, but I don't really try to push really hard to get people off their opiates if they've been on them for a while.
