# Supplementary material for: Development of a pain education resource for people with spinal cord injury
Source: Front Public Health. 2023 Jul 24;11:1197944. doi: 10.3389/fpubh.2023.1197944 (PMC10406314; doi:10.3389/fpubh.2023.1197944)
Supplement: Supplementary file 2 [file Data_Sheet_1.PDF]

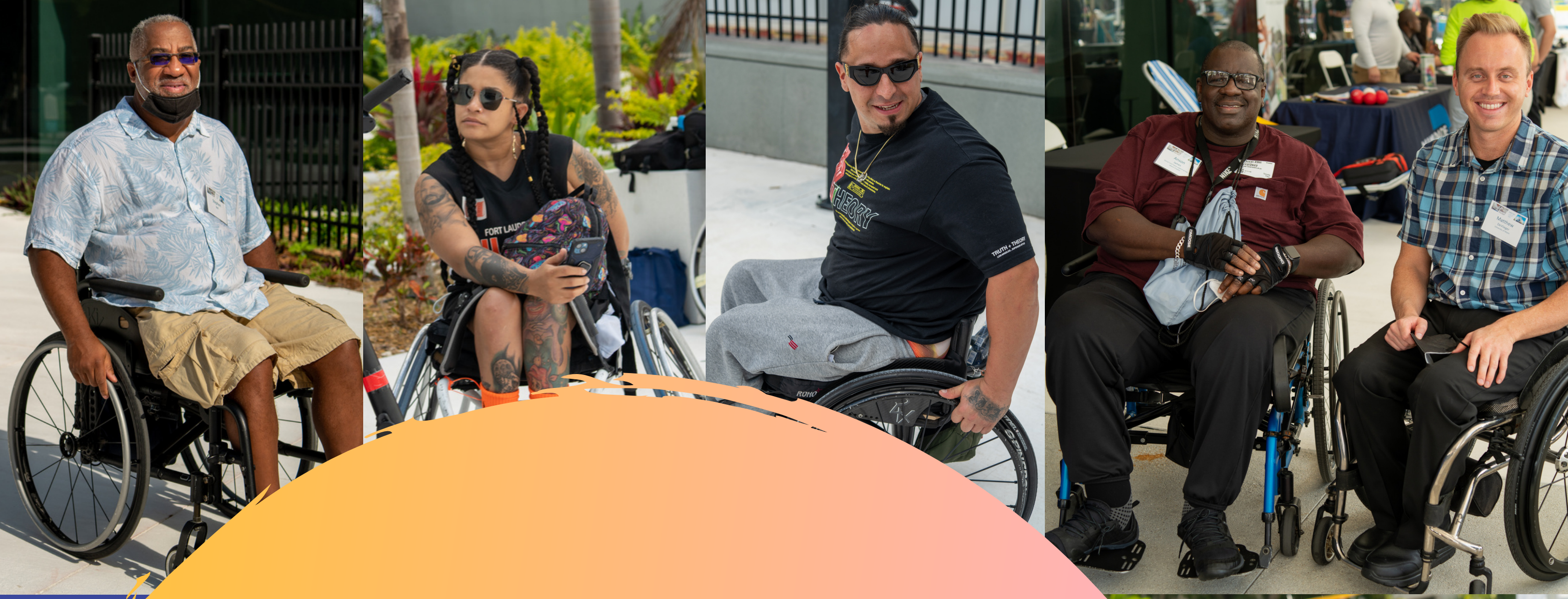

Clinical Pain Research Laboratory

SeePain

Information about pain... for you with a spinal cord injury, and for your significant others, family members, and caregivers.

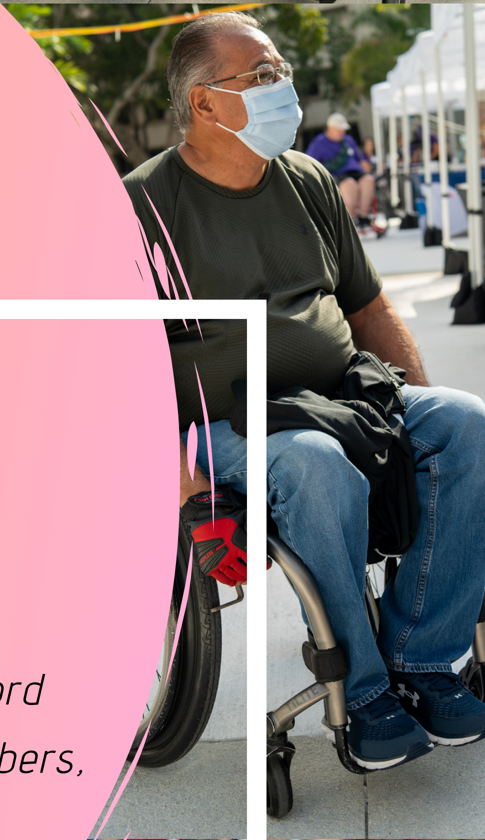

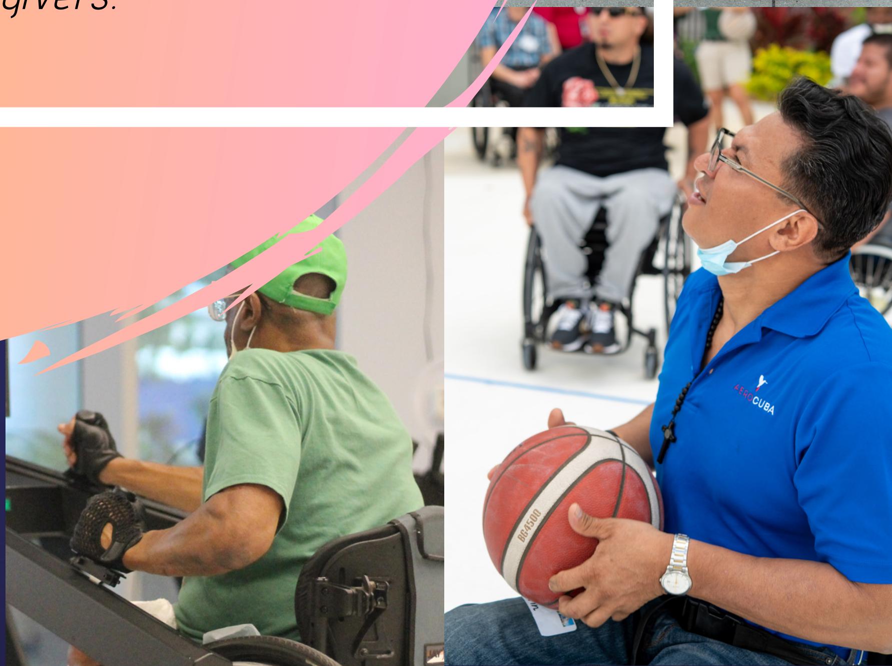

THE MIAMI PROJECT TO CURE PARALYSIS
est. 1985

THE AUTHORS

The SeePain is a team effort including our research team and our research participants.

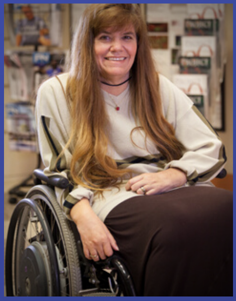
Kim Anderson

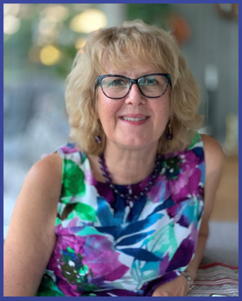
Eva Widerstrom-Noga

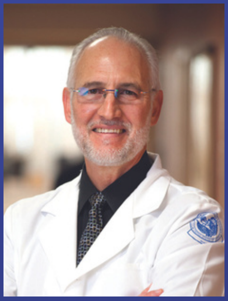
Alberto Martinez-Arizala

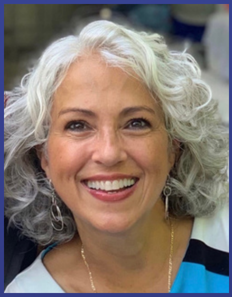
Salome Perez

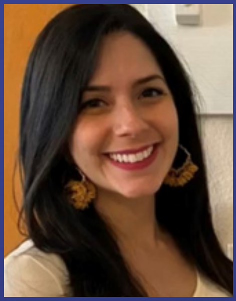
Lindsey Calle-Coule

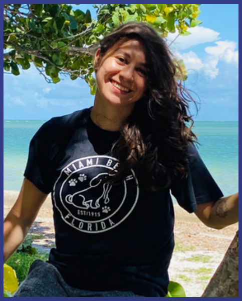
Linda E Robayo

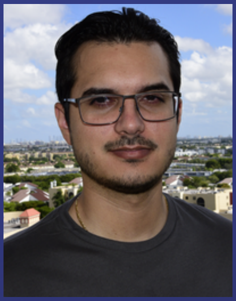
Gabriel Fernandez

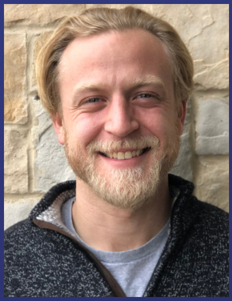
Nicholas Cherup

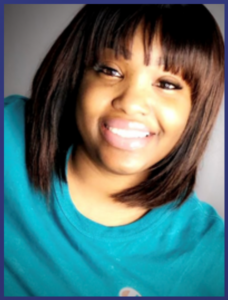
Loriann Fleming

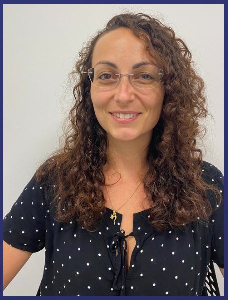
Roberta Vastano

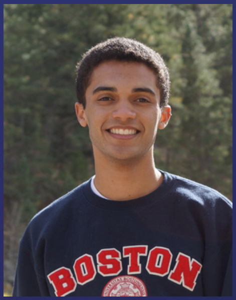

Tyler Phillips

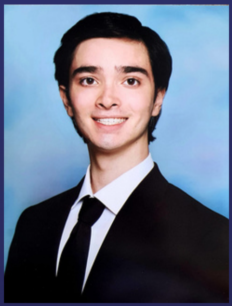
Raphael Briceno

Additional contributors: Jim Adcock, Maydelis Escalona, Jessica Cambridge, and Jaqueline Tibbett.

This work would not have been possible without the generous support of the Craig Neilsen Foundation, the Department of Defense, and the Miami Project to Cure Paralysis.

U.S. Department *of* Defense

CRAIG H NEILSEN FOUNDATION

BACKGROUND

Up to 80% of people develop some pain within their first year after a Spinal Cord Injury (SCI). Pain can develop while you are still in the hospital, or it can develop when you get back home. Pain is very common and is felt differently by everyone. After SCI, people have many important questions about their pain. The SeePain provides information in two educational modules. The first module focuses on the pain itself, and the second module focuses on different ways to manage pain.

The SeePain includes a lot of information; depending on your background and interest you may find some parts of the SeePain to be more interesting than others. If that is the case, you can focus more on the parts you like better or can relate to the most.

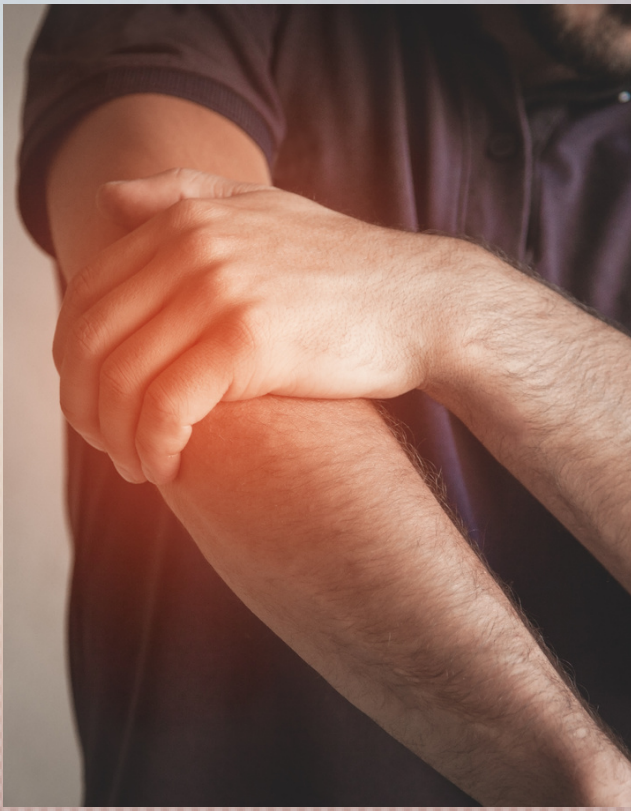

The main purpose of the SeePain is to help you understand your own pain and treatment options to make your chronic pain more manageable. After each module, there is space for you to make notes about what your pain is like, what makes it worse or better, what you expect, what treatments you are interested in, and other thoughts or questions that you may have that you want to discuss with your doctor.

“I read the whole thing and... it helps you understand it ..... And all the stuff that I learned on my own without nobody telling me, everything is here.”

“It almost had me in tears because I had no way to explain this to anyone. And when I explain it to them, I know they don't get it...But the quotes that you have down here, they send chills up and down.”

“It was interesting...the other side of the story, to hear the health care providers, the doctors... And it helps ... me see how to better try to communicate with them so they can understand. Because for them it's also like they don't understand your pain.”

THE SEEPAIN

We chose the name SeePain because pain is a personal experience that cannot be "seen" by others. Many people do not tell others that they are in pain even when their pain is severe, and they just carry on with their daily life. No one, not even your significant other, family member, or healthcare provider can fully understand what your pain feels like. That is why it is so important to discuss your pain and treatment options with your healthcare provider.

The SeePain was developed with the help of people living with an SCI and chronic pain, their significant others and family members, SCI healthcare providers, and scientific literature. The SeePain includes people's personal opinions and experiences (in purple) on how different pains feel, how pain can affect daily life, how people self-manage and cope with their pain, and what their opinions and experiences are with different treatment options. Please note that the quotes in purple are personal opinions that may not apply to you. We have also included information about the mechanisms of different kinds of pain that people may experience after SCI, information about pharmacological and non-pharmacological treatment options, and what happens with pain over time. We hope that the information provided here will help you when you discuss your pain and treatments options with your doctor, your family, and significant others.

In the two modules of the SeePain, you will learn about:

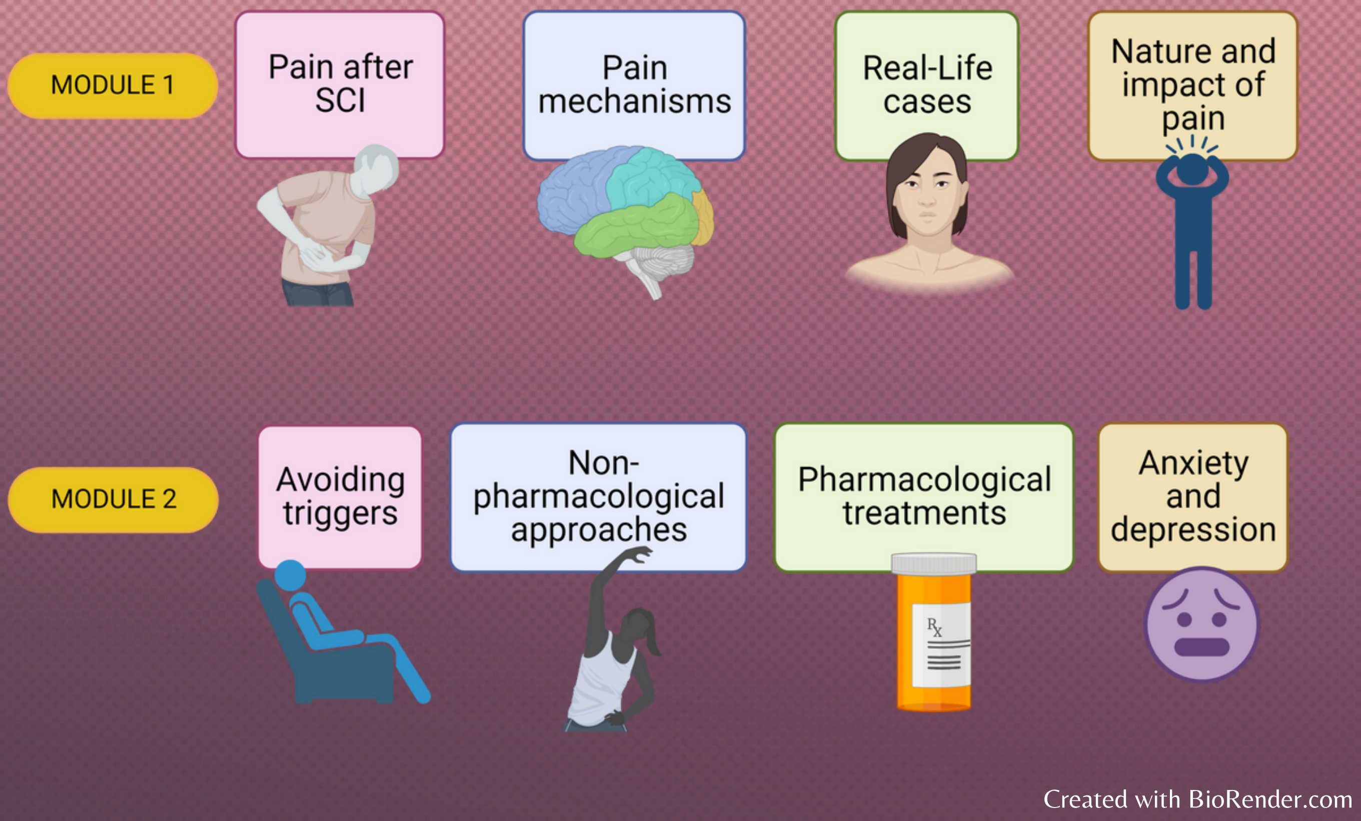

TABLE OF CONTENTS

MODULE 1

What is pain?	3
Pain after SCI	4
SCI classification	5
Types of pain after SCI	6
Real-cases of Pain	14
Chronic nature of pain after SCI	17
Impact of pain	18

WHAT IS PAIN?

“Pain is defined by the International Association for the Study of Pain as “An unpleasant sensory and emotional experience associated with, or resembling that associated with, actual or potential tissue damage.” This means that your pain experience is dependent on several different factors including what type of pain you have, how pain affects your life, and how you think about your pain.

“The pains that occur after SCI vary widely in where they are felt, what they feel like, how they respond to treatments, and how they affect your quality of life.”

What type of pain do you have? What does your pain feel like? How constant is your pain?

Do you feel depressed, anxious, worried, happy, calm...?

Your Pain Experience is Personal

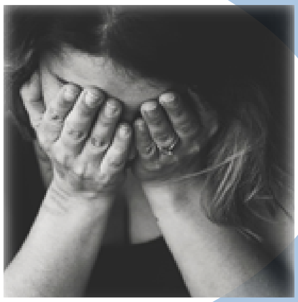

Does your pain negatively influence your activities, independence, mood, or sleep?

Is your pain manageable? Can it be reduced? Does it take multiple approaches?

“It’s really hard ... first to deal with the spinal cord injury ...in everyday life, you have to deal with the pain....The pain is... unbearable sometimes and sometimes you get...immune to the pain...but there are days you can’t deal with the pain and you have to find ways to make it less for you...”

Pain after SCI

The nervous system

The central nervous system consists of the spinal cord and the brain, and the peripheral nervous system consists of all the peripheral nerves (see Figure 1). The central nervous system merges all the information from our senses and coordinates all body activities.

The nervous system is like a computer where the brain is the central processor and the mouse and keyboard are the limbs that sense external stimuli and send electrical signals to the central processor.

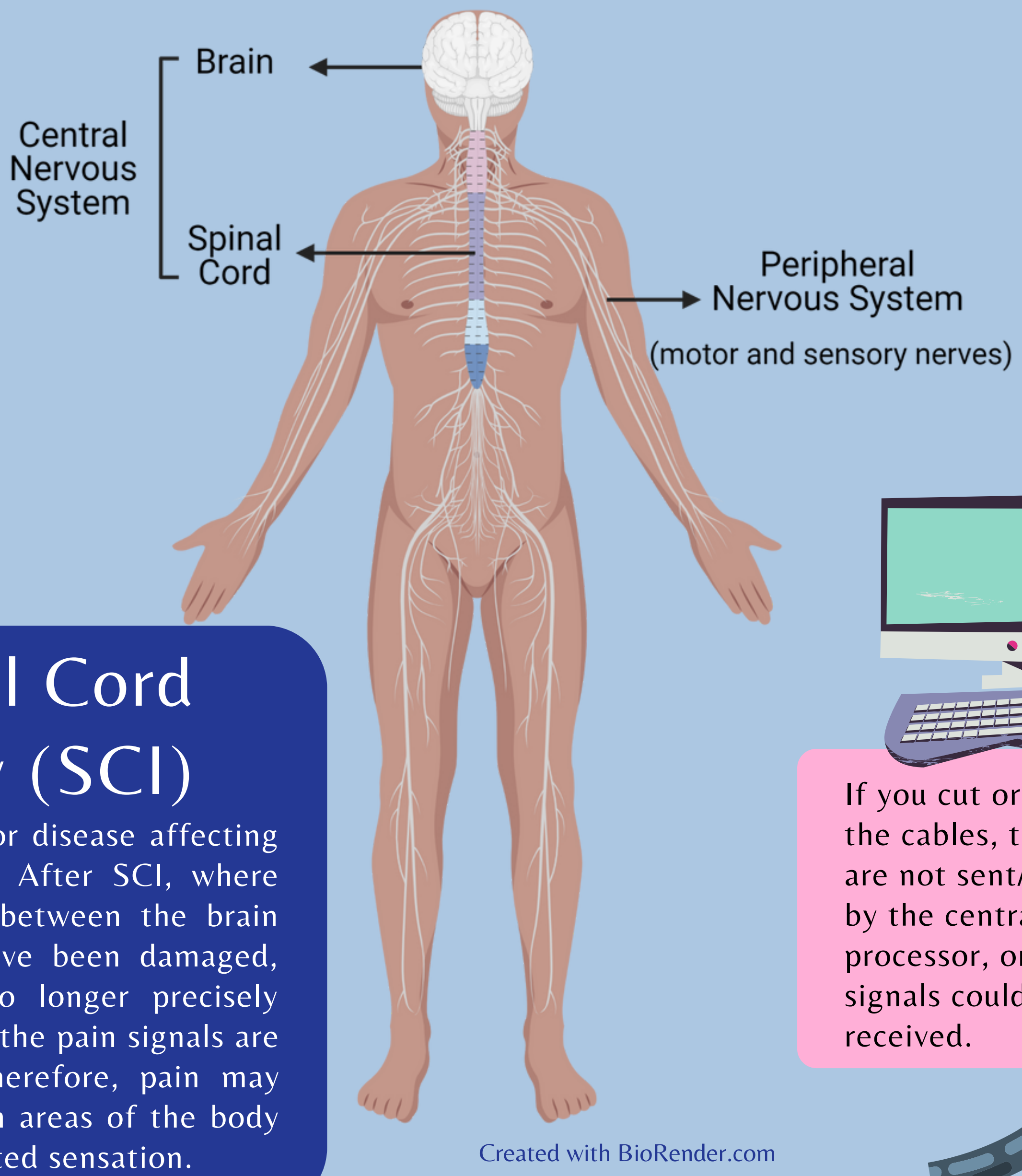

Figure 1: The nervous system

Spinal Cord Injury (SCI)

SCI is a trauma or disease affecting your spinal cord. After SCI, where the connections between the brain and the body have been damaged, the brain can no longer precisely determine where the pain signals are coming from. Therefore, pain may be experienced in areas of the body with none or limited sensation.

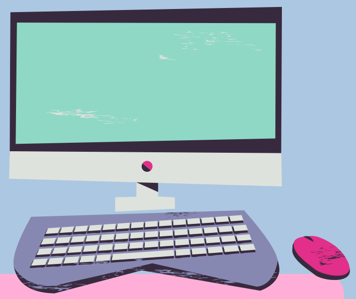

If you cut or damage the cables, the signals are not sent/received by the central processor, or wrong signals could be received.

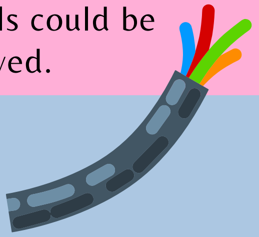

Spinal Cord Injury Classification

To better understand pain in general, it is important to know some basic details about the spinal cord. Sensory nerves enter the spinal cord between each vertebra and provide sensation for a specific skin area or "dermatome" of the body. Similarly, motor nerves exit the spinal cord at each level and connect to muscles. The “neurological level of SCI” is the section of the spinal cord where the injury occurred (See Figure 2).

The ASIA Impairment Scale is the most common method to determine the severity of SCI. When the SCI is classified as complete, there is a complete loss of sensory and motor function below the level of injury with some exceptions. In contrast, when the SCI is incomplete, some sensation and/or movement is present below the level of injury. The determination of the level of injury is not only important for determining how severe an injury is but also for determining the pain type. For example, the nerves that exit the spinal cord at the C6 level supply a part of the hands so an injury at the neurological level of C6 may affect the sensation and movement of the hands and the skin areas below. We will discuss this later in this text.

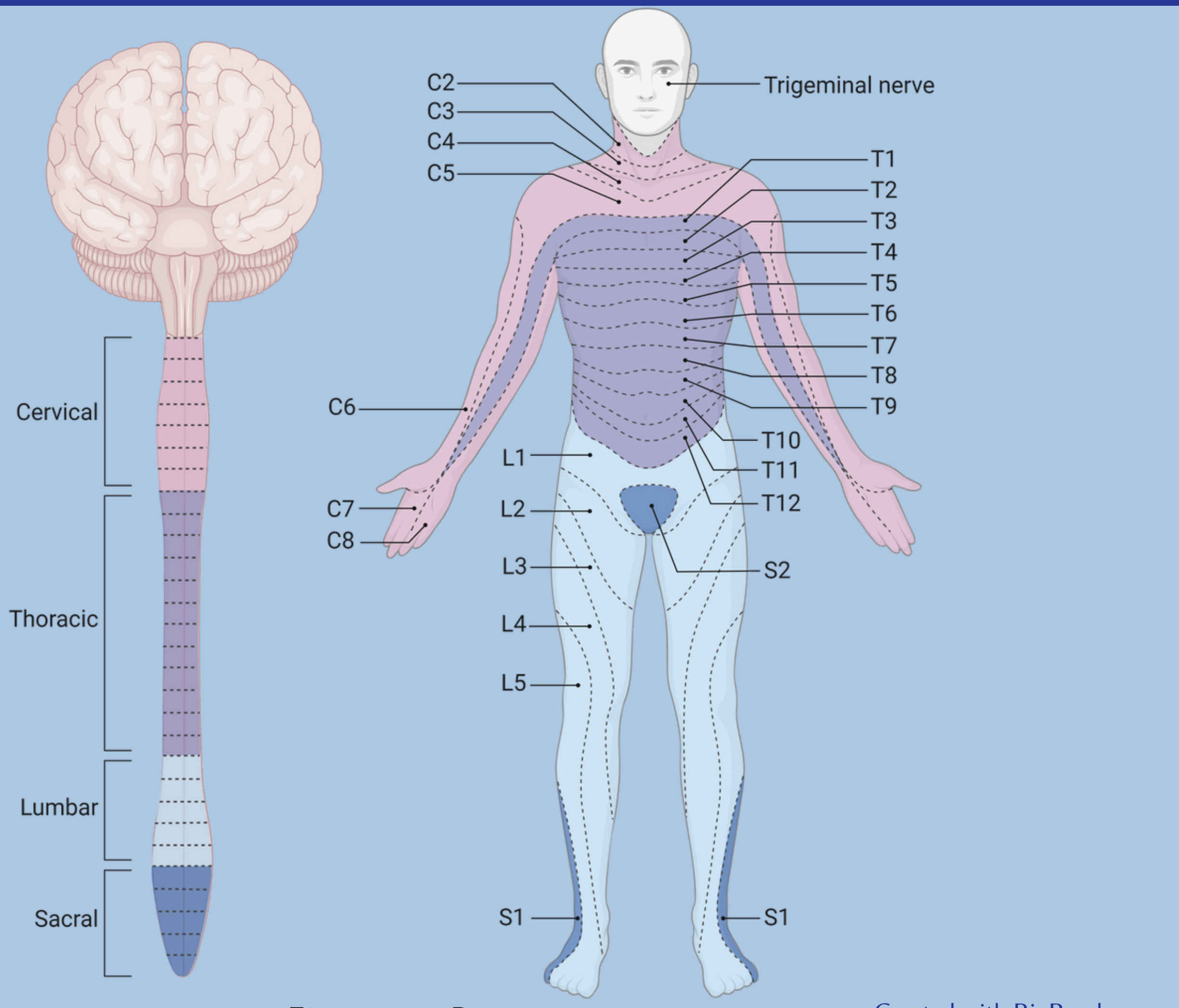

Figure 2: Dermatomes

Types of Pain after Spinal Cord Injury

Most people experience more than one type of pain after their SCI. There are two major types of pain after SCI: 1. Nociceptive pain (no-ci-cep-tive), is a pain that a person without an SCI can also experience, such as muscle pain, and 2. Neuropathic pain (nyoor-oh-path-ik), which is pain directly caused by the injury to the spinal cord.

Nociceptive Pain

Nociceptive pain happens when nociceptors (sensory neurons that respond to noxious stimulus, located throughout the body, including on the skin, in muscle, or internal organs) are activated by specific stimuli including strong mechanical pressure, inflammation, or hot or cold temperature. Similarly, the over-extension of a muscle or joint can also activate nociceptors and produce pain (see Figure 3). When nociceptors are activated, they send signals to the spinal cord and to the brain. Then, your brain processes these signals and can generate pain.

Nociceptive pain can be acute (short-lasting), or it can become chronic. When pain has lasted more than 3 months it is considered chronic. There are many reasons why pain becomes chronic including re-injury and inflammation which can happen when a muscle is used a lot. After an SCI, a nociceptor can only be activated in an area with full or partial sensation. That means that nociceptive pains are most common above the level of injury but can also happen below the level of injury if the SCI is incomplete.

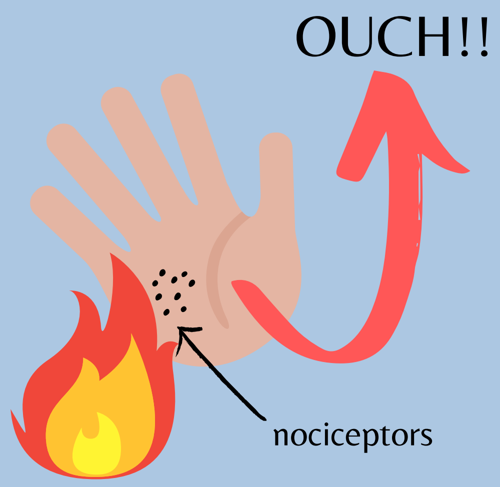

Figure 3: Simple representation of nociceptive pain

The Nociceptive Pain Signal

From the skin to the brain.

The figure below shows an example of how a stimulus activates the nociceptors, which then generate an electrical signal that travels to the spinal cord and then to the brain, reaching brain regions that analyze the stimulus and process the signal. However, the spinal cord can modulate the signal before it reaches the brain.

Different aspects of pain are processed by several brain regions that communicate as a network. Some brain regions are more involved in localizing the pain, determining how intense it is, and what it feels like; whereas other regions are more involved in how one responds to pain with respect to emotions and actions. Therefore, it is common that people who experience chronic pain also feel anxious or depressed. In addition, some brain regions can also modulate the sensory and emotional aspects of pain.

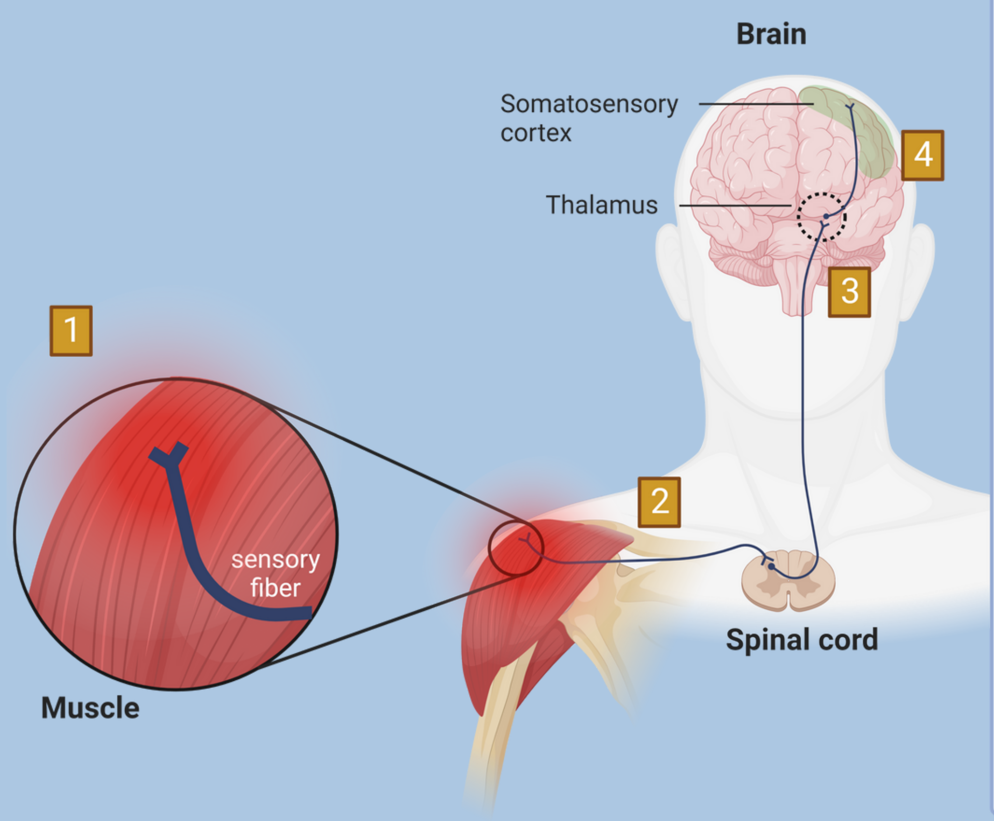

After SCI, shoulder muscles are often overused, which can cause inflammation in that area. Inflammation can make the nociceptors more sensitive than normal.

1. Mechanical stimulation such as movement of the shoulder or pressure on that area can activate the nociceptors (sensory neurons) that convert the stimulus to an electrical signal.
2. The signal travels along the sensory fibers to the spinal cord and then to the brain.
3. The thalamus receives the signal and then activates other sensory, cognitive and emotional brain regions.
4. The pain is generated as an output from multiple brain regions that interact with each other.

Figure 4: The pain signal and pathway from the muscle to the brain

Created with BioRender.com

Nociceptive pain after SCI: What does it feel like?

The most common type of pain after SCI is nociceptive musculoskeletal pain. This type of pain involves nociceptors in muscles, joints, and bones. For example, shoulder injury from daily wheelchair use is a common cause of musculoskeletal nociceptive pain (figure 5). Musculoskeletal back and neck pain are also common and may be caused by sitting for long periods in a wheelchair, or because of muscle spasms. Another type of pain is nociceptive visceral pain (figure 6). This type of pain is caused by the activation of nociceptors in internal organs. It may be caused by constipation or a kidney stone but may not be felt the same way in those who have an SCI compared to uninjured people. It is important to consult with your doctor if you experience new pain in the abdominal area because there could be many reasons for this type of pain.

" I still can't sleep on my left side because of the shoulder pain".. "twenty-thirty minutes is fine then the shoulder will start throbbing and I... roll around and get the weight off of it."

"When it's really bad ...my stomach.. hurts a lot and I can't eat.. maybe I'll eat less, I will try to rest, Sometimes getting out of the chair just allows me to relax the stomach, the pressure on the area."

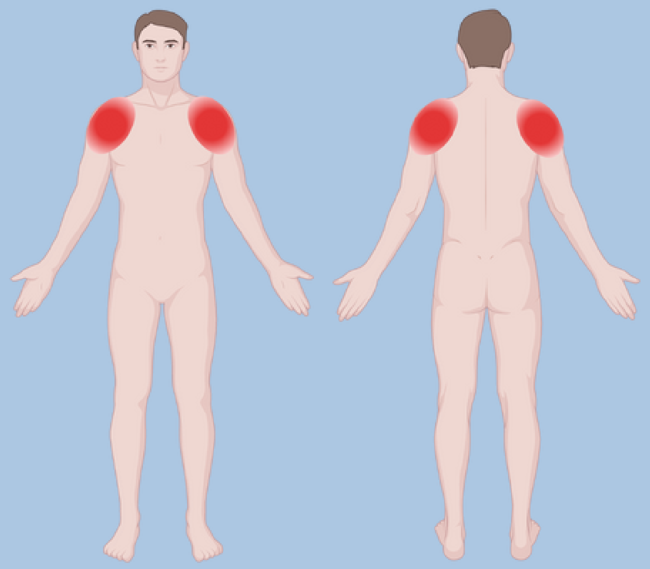

Figure 5: Example of musculoskeletal pain

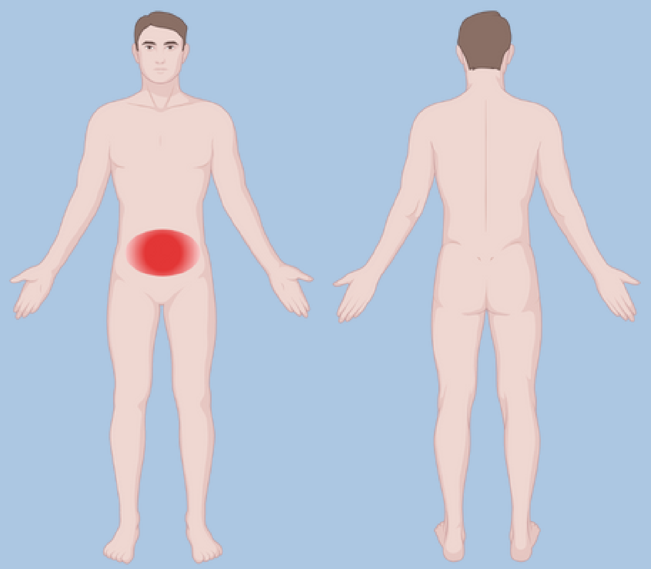

Figure 6: Example of visceral pain

Created with BioRender.com

Neuropathic Pain

Neuropathic pain can develop weeks to months after an injury to the brain, spinal cord, or peripheral nerves. This type of pain is associated with multiple molecular and cellular changes in the spinal cord and brain. Neuropathic pain can last long after the initial injury has healed and occurs in about 60% of people who have had an SCI.

Neuropathic pain after SCI is divided into "at-level" and "below-level": At-level neuropathic pain occurs around the level of SCI and can feel like a corset or belt, or something squeezing around the abdomen. This pain usually starts early after SCI. Below-level neuropathic pain occurs below the level of spinal cord injury and usually develops over the first year of injury. If below level neuropathic pain develops after the first year you need to consult with you doctor to determine why this is happening. The figure below shows two different examples for these two different types of pain in an individual with an injury around Thoracic level 4 (T4) which is marked with an "X".

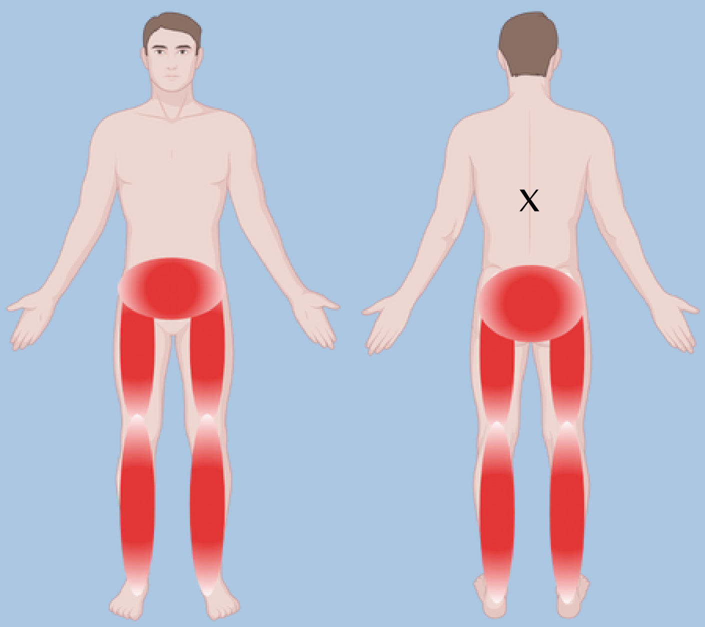

Figure 7: Example of below-level neuropathic pain

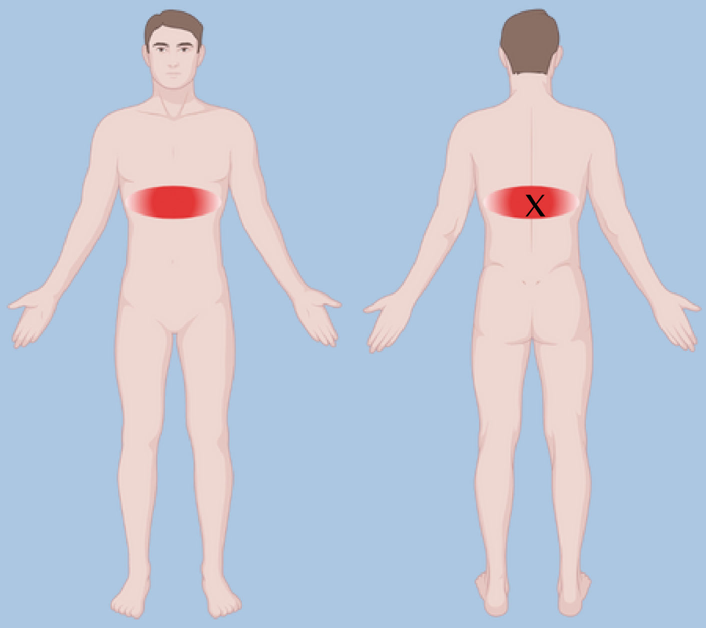

Figure 8: Example of at-level neuropathic pain

Created with BioRender.com

Why does neuropathic pain happen??

There are multiple mechanisms that can contribute to neuropathic pain after SCI.

Unfortunately, the mechanisms that cause and this pain are not completely understood, and they can also vary a lot between individuals. This makes it very difficult to determine the cause of pain and the best treatment for each patient. Some of the common causes of neuropathic pain are discussed briefly below:

1. Central sensitization

After an injury, the neurons that carry pain signals in the spinal cord and brain can become very sensitive (easily activated). Thus pain can be experienced more easily in response to a stimulus or even without any stimulus (spontaneous pain). Central sensitization can cause hyperalgesia and allodynia. Hyperalgesia occurs when a stimulus that normally causes mild pain (like pinprick) produces severe pain. Allodynia happens when a stimulus that is not normally painful (such as light touch or cool temperature) triggers pain. A common example that many people have experienced is a skin burn.

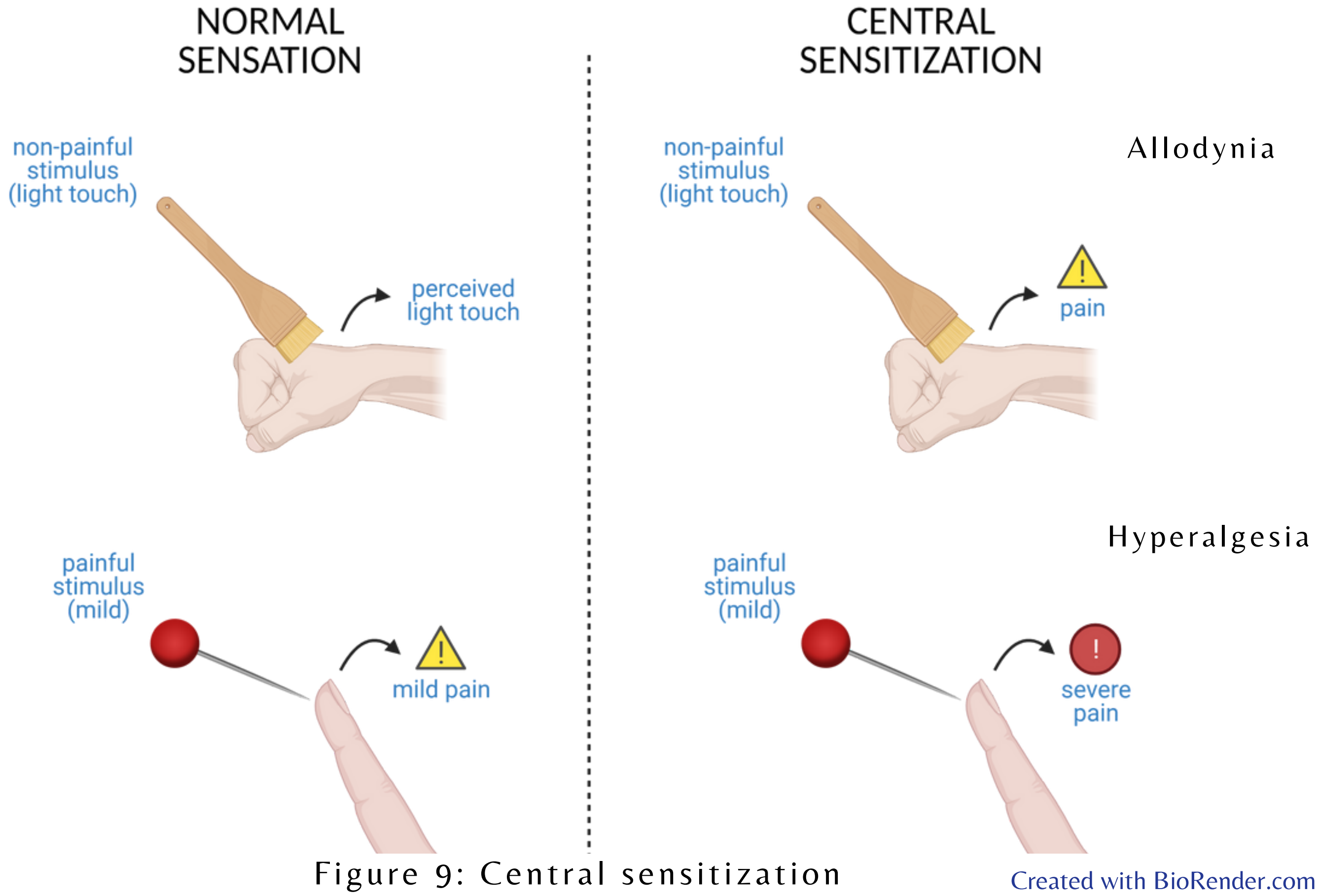

Figure 9: Central sensitization

2. Reduced inhibition of pain signals

In an uninjured individual, there are systems in the brain and the spinal cord that naturally reduce pain when we are presented with a painful stimulus. When the spinal cord is injured these systems are not as effective and as a result, neuropathic pain can develop. You can think of this mechanism as a "brake" and when the brake is not working the pain signals reach the brain at a greater strength than they would do if you did not have an SCI.

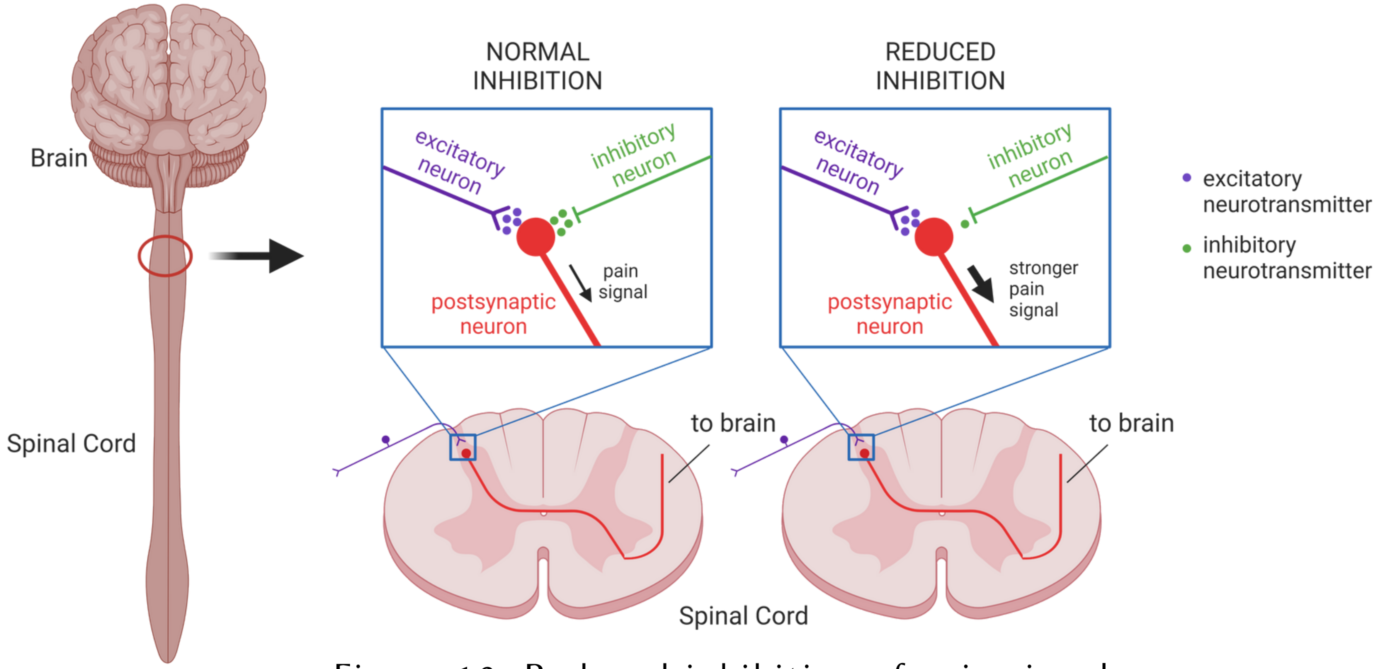

Figure 10: Reduced inhibition of pain signals

Created with BioRender.com

3. Microglial activation: changes to "support cells"

Cells that surround and support the neurons in the spinal cord and in the brain, called microglia can change after SCI and become "activated". This activation causes inflammatory factors and other substances to be released which causes neurons to become hypersensitive to stimuli.

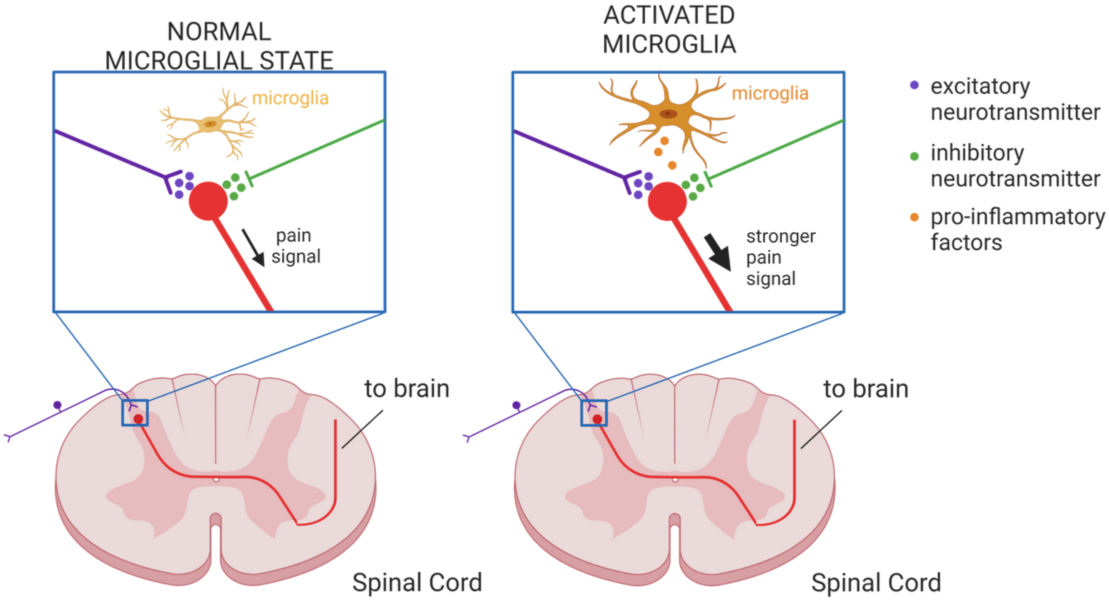

Figure 11: Microglial activation

Created with BioRender.com

4. Neuroplasticity: Reorganization of the nervous system

Neuroplasticity is a very important process for the recovery of function after SCI. However, during this process, changes to the structure of neurons and/or their connections may occur. For example, neurons that normally provide pain signals can make new connections in the spinal cord and brain, which may lead to the development of neuropathic pain. Neurons can also grow new axonal endings which increases the possibility for the development of neuropathic pain.

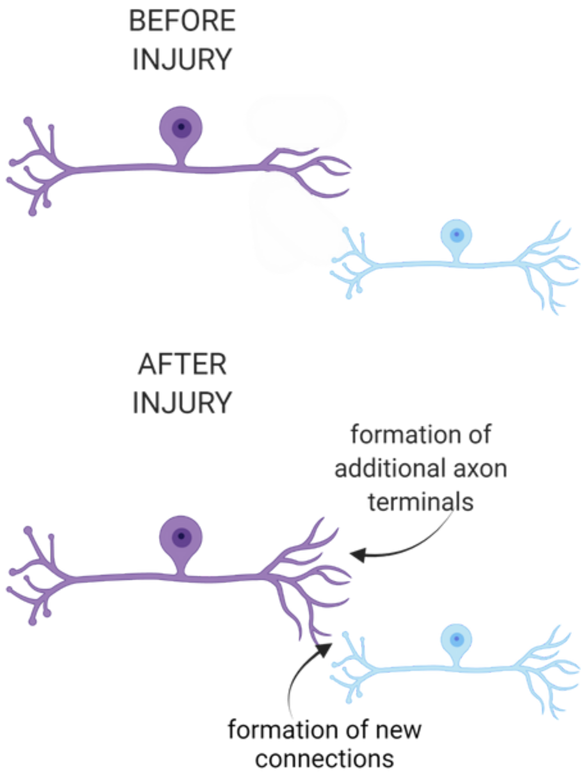

Figure 12: Neuroplasticity

Created with BioRender.com

As you can see from the brief explanations of these four mechanisms, both the spinal cord and brain can become hypersensitive, hyperactive, and reorganized because of the spinal cord injury and all these changes can result in chronic pain. Therefore, most of the medications that are used to treat neuropathic pain target one or several of these mechanisms. This will be discussed further in the next module.

Examples of neuropathic pain

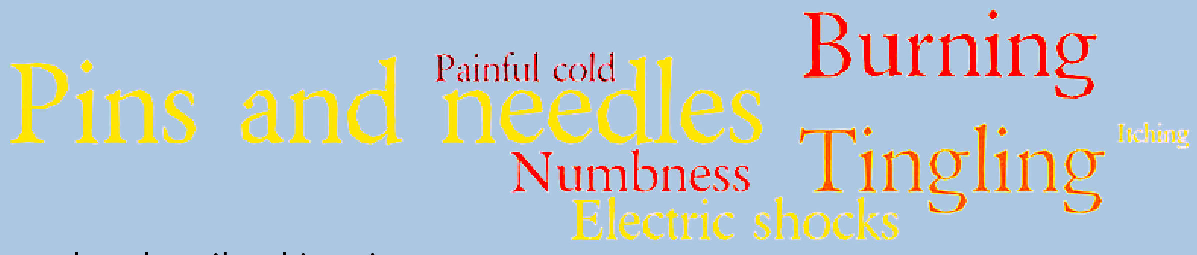

Other words used to describe this pain are "pricking", "sharp", "stabbing", "shooting", "lacerating", "squeezing", and "aching."

<u>People may describe this pain as a deep pain:</u>
"Pressure.. not brushing, not poking, it's deep inside... not skin surface pain".

<u>Neuropathic pains may be constant or intermittent:</u>
"as if you were to have your legs put into a massive vice grip and you're constantly squeezing. It's a very hard throbbing, squeezing pain constantly all day, all night."

"It's, intense um it's not necessarily constant; it does stop.... just like suddenly I got stabbed and I'm like whoa."

<u>Pain can be unpredictable:</u>
"The pain just kind of comes.... it's very unexpected."

"It's like a total 'I'm free' and I'm thinking 'oh yes the sensation is coming back the nerves are regenerating' and the next day I'm in excruciating pain."

<u>People may feel pain in response to something that is normally not painful:</u>
"If somebody were to just hold my hand, the light touch kills me.. it's too much, like even now ...if I keep my hands here they're like a burning fiery so, yeah I don't do touch."

"Temperature really affects the way I'm feeling. The colder I am the more miserable I am cause everything gets really tight, the hyper-sensitivity gets worse..."

<u>Pain may worsen over time:</u>
"I had pain right away but every year it's been getting worse."

<u>Pain can be confusing or strange:</u>
"I feel like I have another body connected to my body when I feel the pain."

"This pain is kinda tricky... when I try to do one thing to combat it, it comes in another way."

"The pain from the nerve pain... it's like it can do a 180 in like a second"

"I thought I pinpointed it and then the pain.. does what it wants to do, I can't control it.. it comes and goes"

Real-life cases of pain

People with SCI often have several types of pain in different places of their bodies. For example, an individual may have three different pains in different parts of his/her body at the same time and each of these pains can be different and require different treatments. Therefore, it is important that you let your healthcare provider know if you have more than one pain, where you have that pain, and what it feels like. A helpful way to start the discussion with your doctor is to use a pain drawing (figure of a body where you can shade in each pain). Below are 3 examples of real-life cases of pain, each includes a pain drawing and description.

Please note that how pain is felt varies a lot between different people and these examples may not reflect your pain.

Case #1

Incomplete C5 level of injury: This person experiences three different pains. The worst pain is a neuropathic pain in the hands, which is located at the neurological level of injury. The second worst pain is a nociceptive musculoskeletal pain in the neck and shoulders, and the third-worst pain is neuropathic pain below level of injury.

"I have pains in both hands from the wrist down, its mainly.. stinging and numbness and also in the right foot... some pain in the neck but it only comes from sitting.. like in front of a computer or just being in one spot trying to pay attention...then I have pains in the neck and shoulder."

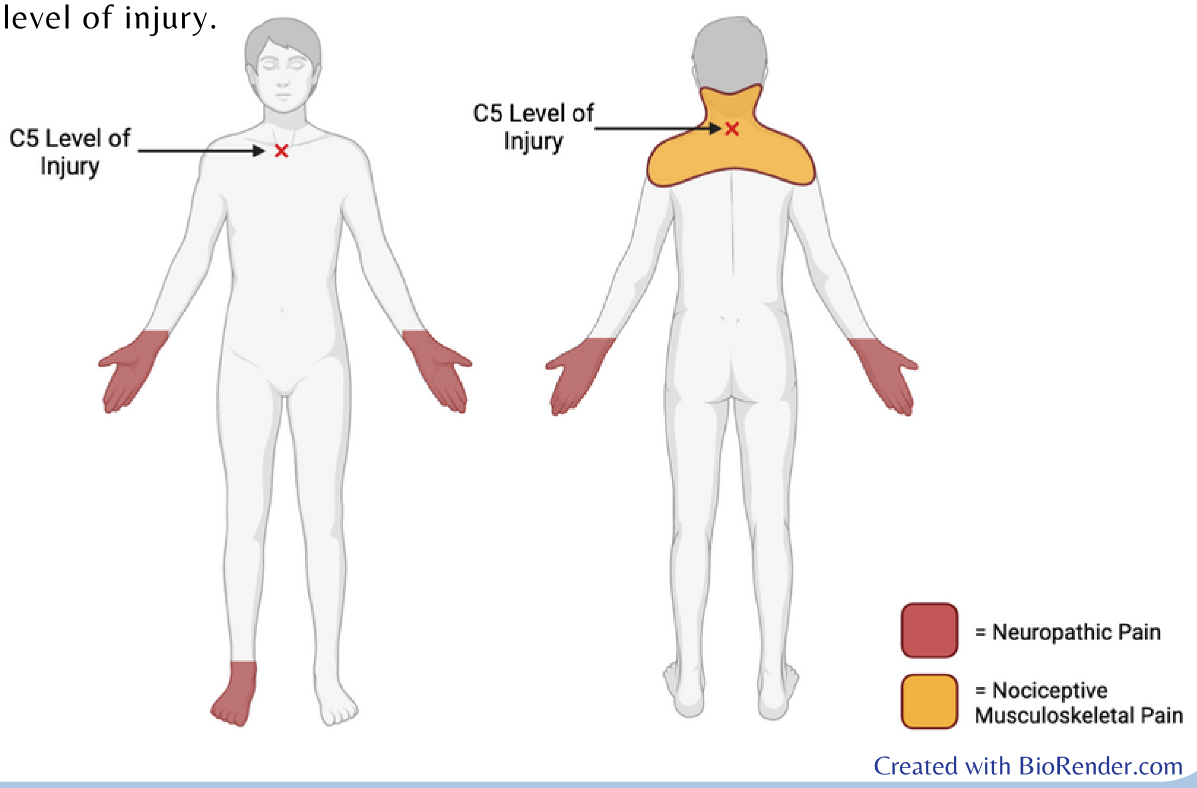

Case #2

Complete T2 level of injury: This person has three pain problems. The worst pain is the below-level neuropathic pain in the lower legs and feet, the second-worst pain is the at-level neuropathic pain with allodynia in the chest area, and the third-worst pain is the nociceptive musculoskeletal pain in the lower back.

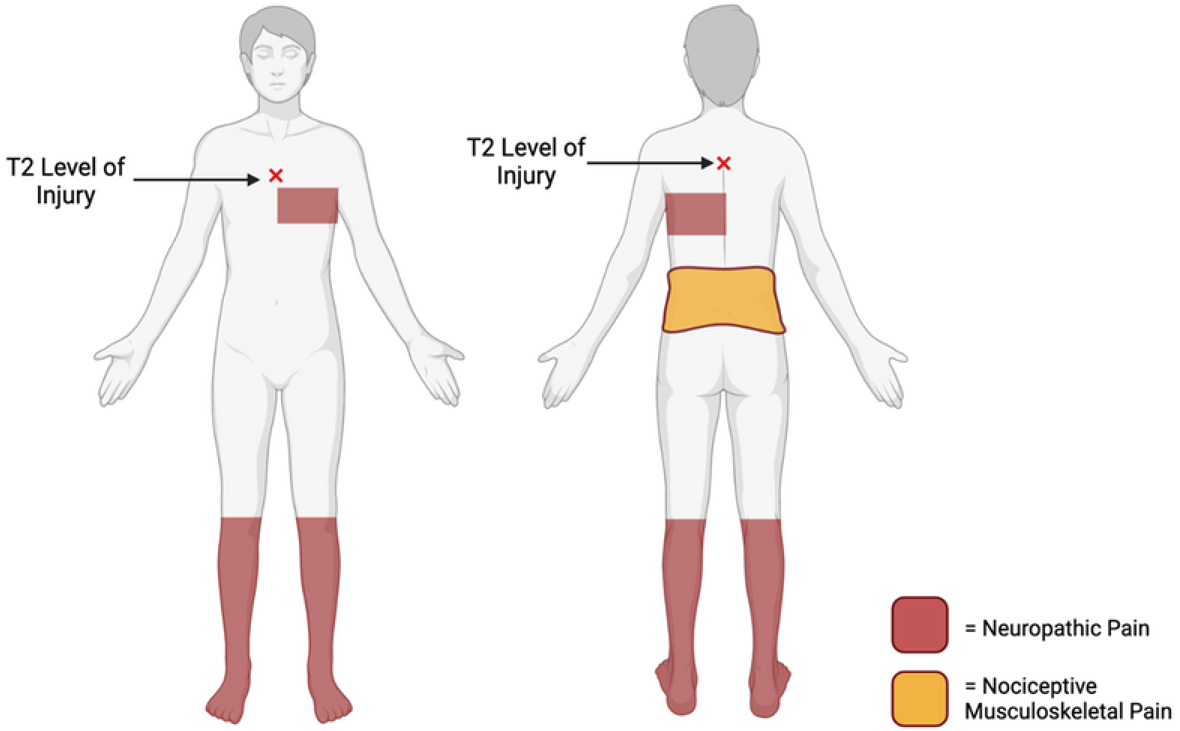

Created with BioRender.com

"My legs... The pain there is as if you were to have your legs put into a massive vice grip and you're constantly squeezing. It's a very hard throbbing, squeezing pain constantly all day, all night. And the .. pain in my back is more of a sharp as if you had a knife or a sharp object sticking someone in their back constantly. The pain in my left shoulder area right around my chest area is very sensitive to the touch ...you can take something as light as a bedsheet and if it touches me it produces pain.... no one can actually touch me in that area and I'm... the only one that touches me in that area because I have grown accustomed to knowing how to, ok now I'm going to touch so I brace myself for the pain"

Case #3

Complete T2 Injury level: This person also has 3 pain problems. The worst pain is the below-level neuropathic pain in the thighs, legs, and feet, the second-worst is the nociceptive musculoskeletal pain in the buttocks area, and the third-worst pain is the nociceptive visceral pain in the sphincter area.

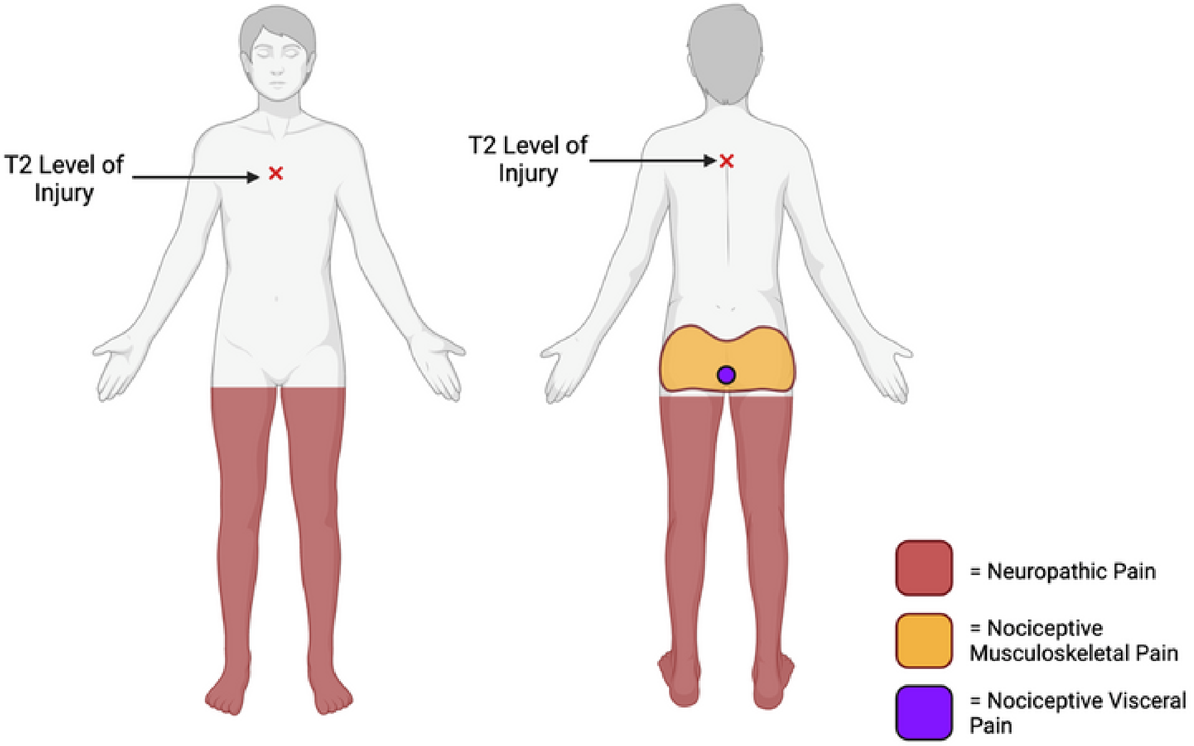

Created with BioRender.com

“I’ve had this spinal cord injury almost fourteen years now and I’ve had the pain since pretty much day one. Kept thinking this is good pain, it’s all about healing...But then when I knew it wasn’t going away it was an everyday thing..it took a while to find the right medication to take for the pain that I was experiencing. Once I got on that it was more controllable so I could live with it more. It took months and that first three months were horrible."
"The legs .... the only thing that aggravates my legs is the cold. It’s like little electric shocks going on, and ..I look at them like they’re good feelings.. it’s like nerves shooting up..”

Chronic Nature of Pain after SCI

While pain's normal role is to let you know when your body is in danger, this may not be the case for chronic pain. After an SCI, neuropathic pain can last long after the initial injury and does not necessarily indicate any current danger or that something must be treated. It is very important to have an accurate pain diagnosis from a doctor who has experience with people with SCI. Getting to know your pain is also very important because worsening of pain may indicate a new medical problem. If you have been injured for more than one year and you develop new pain or your pain suddenly worsens, you should consult a doctor.

The figures below show the percentages of different pain types emerging at 1 month, 6 months, and 12 months after injury, over a one-year period in a sample of people with SCI. It should be noted that in about 25% of people neuropathic pain is present within days after SCI. Neuropathic pain is usually persistent both with respect to where it is located and the quality it has. However, the intensity of this pain may be reduced over time. As you can see in the figure below (blue arrows), most people with SCI (about 80%) develop some pain after their injury and about 60% develop neuropathic pain within the first year after injury. Nociceptive musculoskeletal pain like shoulder pain can develop at any time after SCI and is usually more treatable.

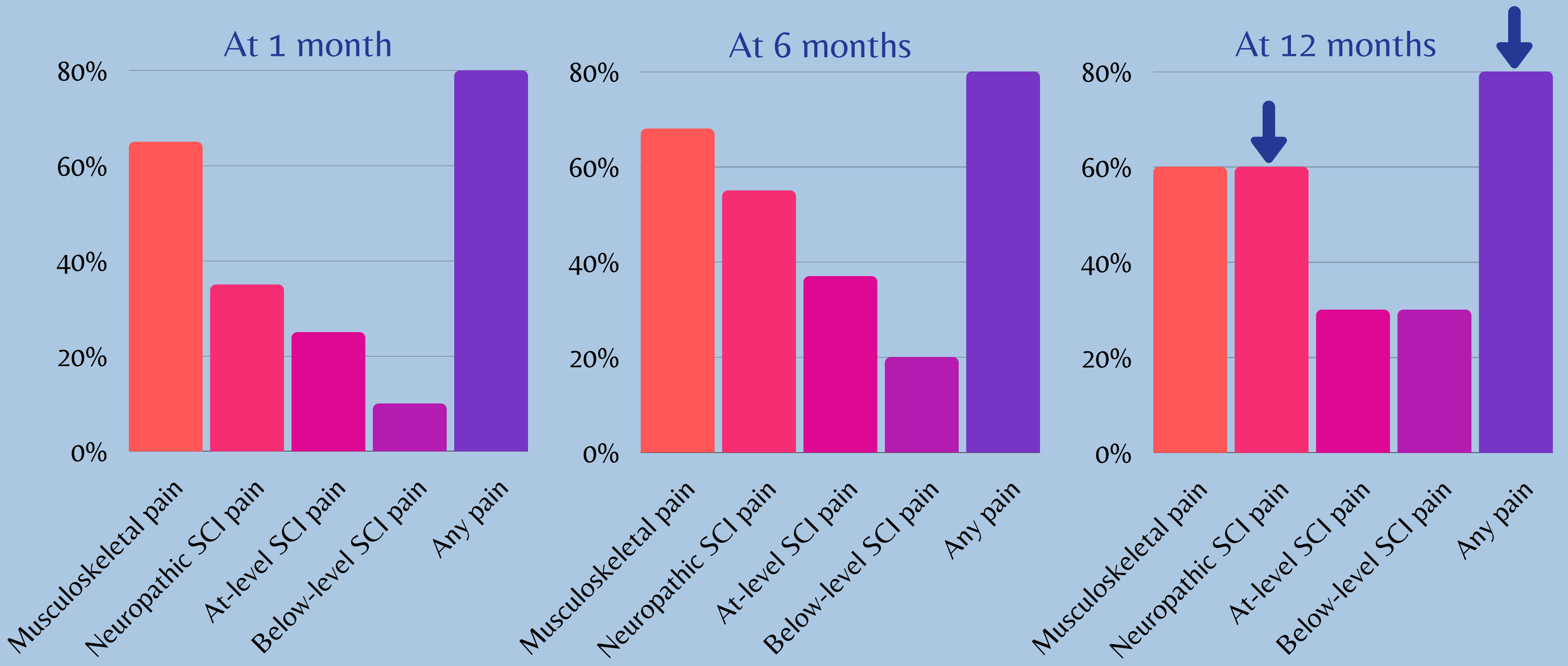

Figure 13: Prevalence of any pain, musculoskeletal pain, and neuropathic SCI pain at 1, 6, and 12 months after SCI

Impact of Pain after SCI

Persistent pain negatively affects quality of life and independent living by interfering with sleep, mood, and daily activities. Below are some perspectives provided by people who experience neuropathic pain on how pain impacts their lives. This does not apply to everyone.

Pain impacts life:

"Pain changes everything even if you get good at ignoring it."

"I think the big thing with spinal cord injury... in general and with pain is just not being able to control it."

"Yes, it affects everything I do. There have been days I wake up and I have to cancel everything"

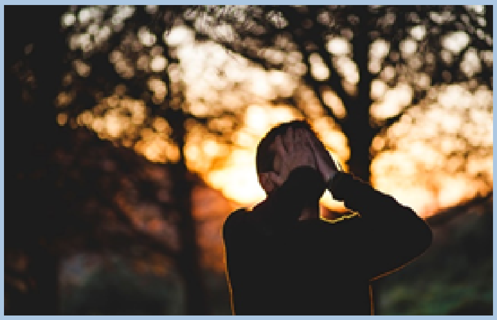

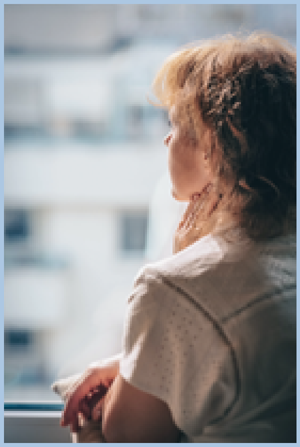

Pain may impact activities and work:

Many people living with SCI and chronic pain report that pain interferes with exercise, household chores, work, and other daily activities.

"I can't.. because if I have the pain I can't. I like to read, I like to watch movies but if I have the pain it's just ... I get very tense and like I don't want to, I want my medicine I don't want anything."

"Folding laundry is a really big pain... just the motion of leaning in to grab something out of the dryer and then trying to balance myself and fold the clothes...it can really irritate my neck and my upper back."

"There are days where I just can't leave the house for hours.., sometimes I don't even leave at all. So yeah I have to reschedule meetings and client visits and stuff."

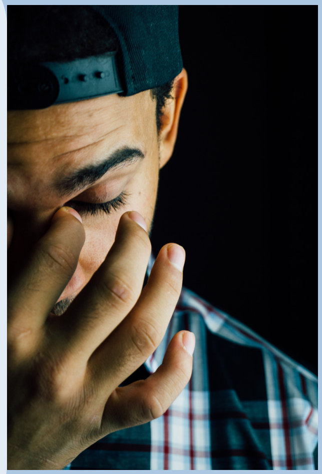

Pain may impact sleep:

Pain can interfere with falling asleep, as well as staying asleep. Therefore, some people may feel very fatigued or tired during the day.

"So... sometimes I only get 2 hours or 1 hour. It's hard for me to go to sleep even if I take amitriptyline that's supposed to knock me right out."

"I even have problems I mean going to sleep because the pain is too much. At the beginning I could sleep all night.. but right now the spasticity and the pain wakes me up."

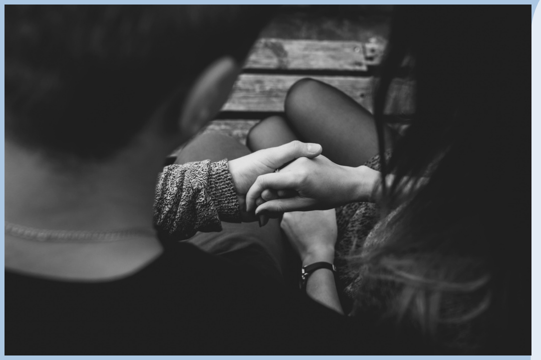

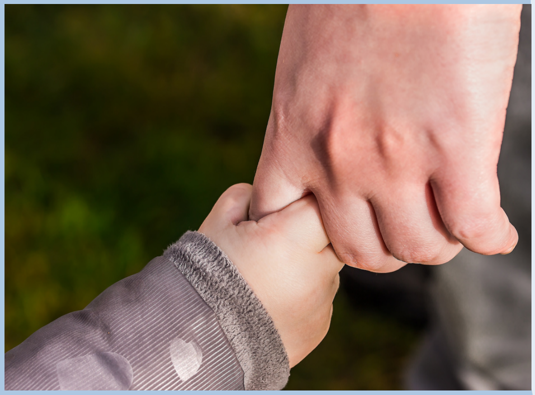

Pain may impact relationships:

Pain and its impact on activities, mood, and sleep can also have a negative influence on relationships and social activities.

"It worries my significant other.... My kids cry when they see me hurting."

"I don't do anything, I don't go out, I think it cost me my marriage cause I don't... like going out, I don't... like getting touched cause it hurts"

"in the evening it escalates more so if one of my kids has a baseball game or a volleyball game or so I would go and it's like I have to push myself to go. Cause by that time I'm not going to be active so I can't ignore it like I do in the day, I'm active, I'm ignoring I have to sit there and I have to feel the pain at the same time so it affects a lot, it really does."

NOTES ABOUT MY PAIN

HOW DOES MY PAIN FEEL?

HOW DOES PAIN IMPACT MY LIFE?

ANY QUESTIONS I HAVE
