# Supplementary material for: Development of a pain education resource for people with spinal cord injury
Source: Front Public Health. 2023 Jul 24;11:1197944. doi: 10.3389/fpubh.2023.1197944 (PMC10406314; doi:10.3389/fpubh.2023.1197944)
Supplement: Supplementary file 3 [file Data_Sheet_2.PDF]

TABLE OF CONTENTS

MODULE 2

Ways to manage your pain	2
Avoiding triggers	5
Evidence-based treatments	6
Non-pharmacological approaches	6
Recommended treatments	24
Cannabinoids	28
Thoughts about opioid medications	30
Thoughts about pain medications	33
Anxiety and depression	35
Significant others/family/caregivers	37
Final message	40
Resources	41
Wordlist with definitions	42
References	43

WAYS TO MANAGE YOUR PAIN

Many of the pain types that occur after an SCI are long-lasting and can be difficult to treat. However, all pain can improve over time either by itself or because of pain treatment or self-management. Often a combination of different ways to manage pain works best. In this module, we present multiple options, but based on your pain, your treatment goals, and your discussions with your healthcare provider and your insurance, collectively you'll decide what's best for you. A healthcare provider with experience treating people with SCI will consider pain management in the context of SCI.

"... I use the oven analogy. I tell folks that I can't turn off the oven, I'm just turning down the heat a little bit."

It is important to know your pain and what treatments and other approaches can be used to make it more manageable. However, you also need to know what can potentially make it worse. A good understanding of your pain can help you communicate better with significant others, family members, and healthcare providers. It can also be very helpful to learn how other people with SCI manage their pain and their experiences.

Why is Pain Education so important ?

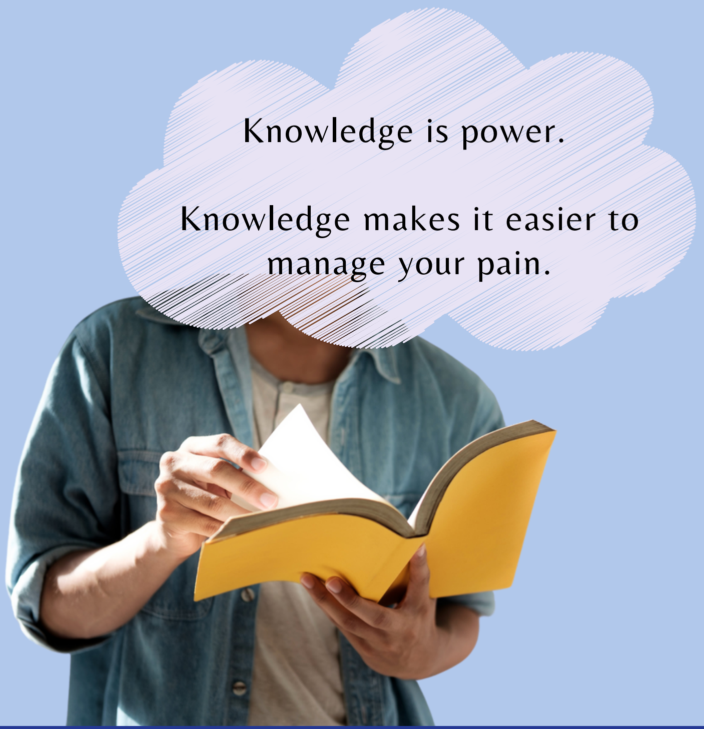

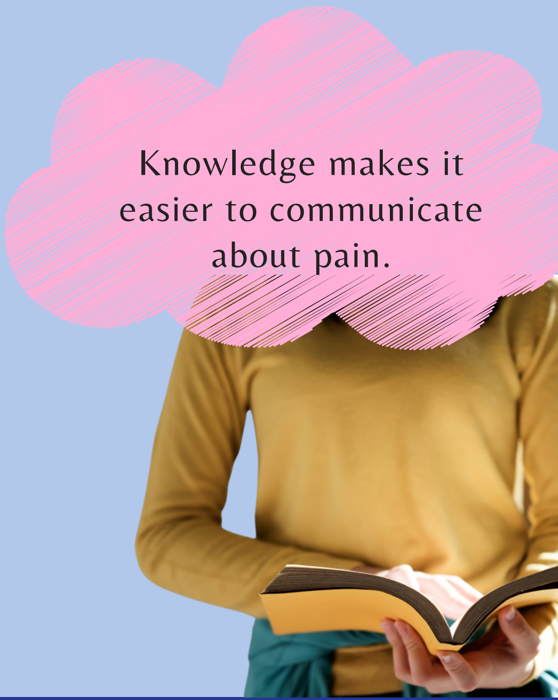

What do people with SCI do to manage their pain?

People who experience persistent pain after their SCI often use different combinations of medications, self-management, and physical and mental strategies to deal with their pain. The graph below shows the results from a 2006 survey asking about which pain treatments people have used and how effective they were in relieving pain. For each treatment, the "average relief" (from 0=no relief to 10=complete relief) was calculated by averaging the pain relief in all participants who had used the treatment. The most common pain medications were over-the-counter medications and opioids. Note that this data predates the use of Pregabalin (Lyrica) and Duloxetine (Cymbalta).

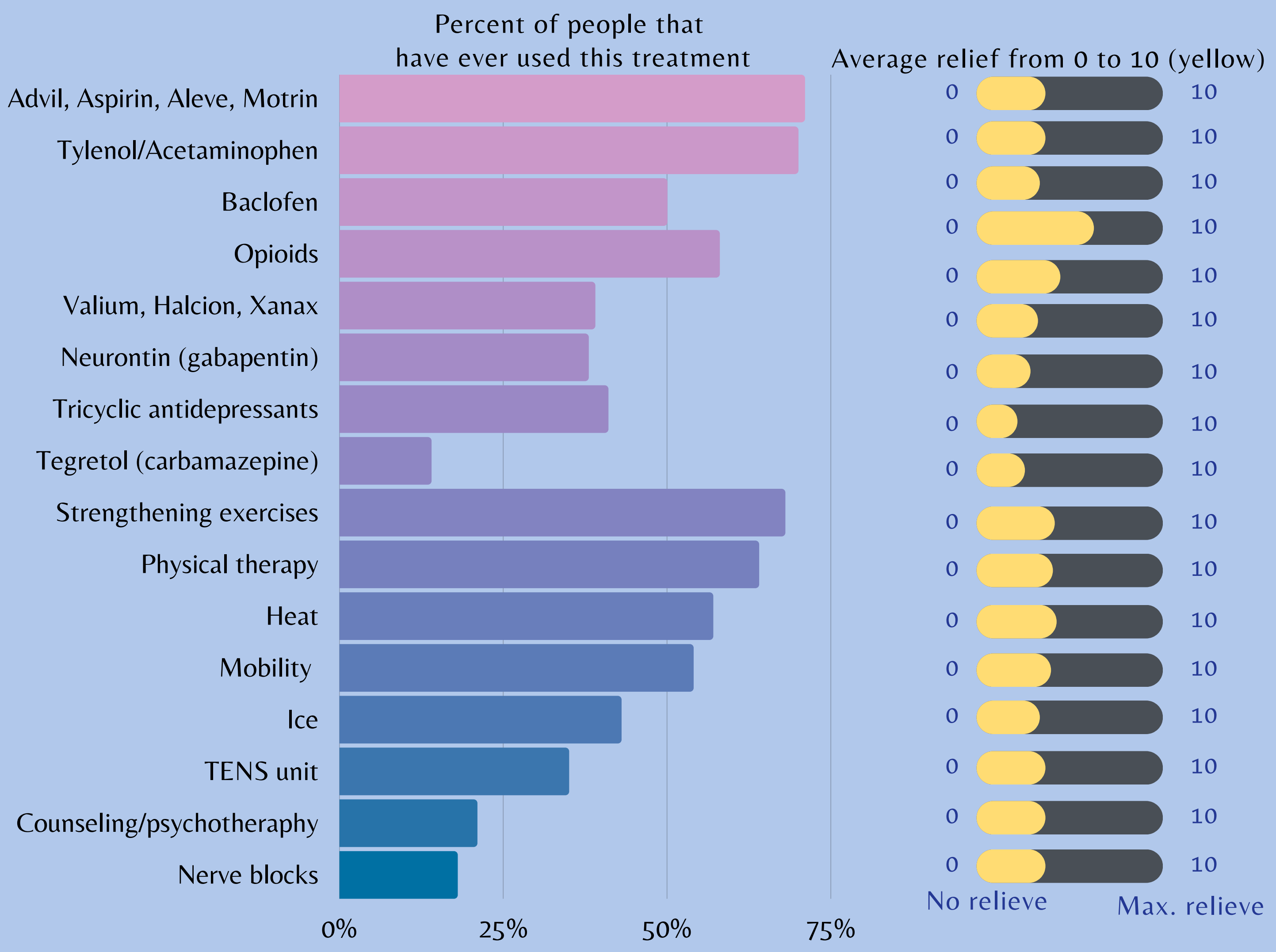

Cardenas, D. D., & Jensen, M. P. (2006). Treatments for chronic pain in persons with spinal cord injury: A survey study. The journal of spinal cord medicine, 29(2), 109–117. https://doi.org/10.1080/10790268.2006.11753864

Treatments perceived to be helpful for neuropathic and non-neuropathic pain

People can experience different pain types, as discussed in Module 1. Two recent studies (Bryce, 2022 and Tsai, 2021) asked people with SCI about the treatments they perceived to be helpful for their neuropathic and non-neuropathic pain in the last 12 months.

Pharmacological treatments that were rated helpful for neuropathic pain included non-tramadol opioids, cannabinoids and anti-epileptics. Non-pharmacological treatments included massage, body position adjustment and relaxation therapy.

Treatments perceived to be helpful for non-neuropathic pain are summarized in the figure below.

A recent study (2021), asked 391 people with SCI about the pharmacological and non-pharmacological treatment they use for their non-neuropathic pain (nonNeuP).

Conclusion:
Analgesic medications, such as NSAIDs, acetaminophen, and opioids are perceived as helpful pharmacological treatments across nonNeuP locations.

Reference: Tsai, CY., Bryce, T.N., Delgado, A.D. et al. Treatments that are perceived to be helpful for non-neuropathic pain after traumatic spinal cord injury: a multicenter cross-sectional survey. Spinal Cord (2021).

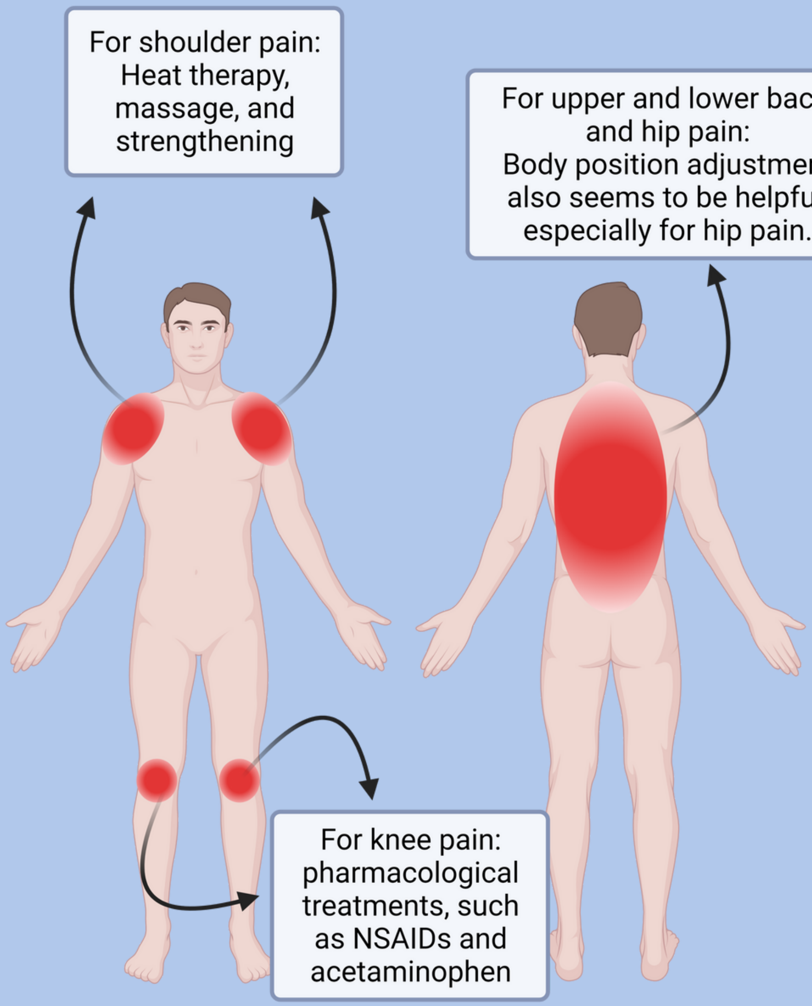

Created with BioRender.com

Avoiding triggers

One very important part of managing your pain is to figure out what factors and situations that can worsen pain.

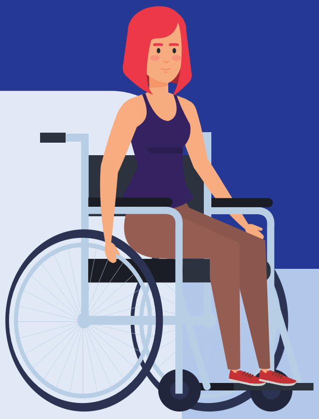

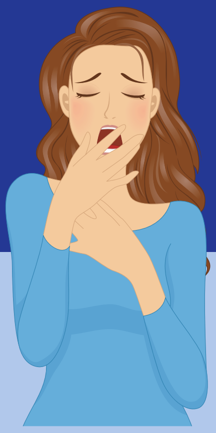

Common situations that worsen pain include:

- Prolonged sitting without changing position
- Infections (e.g., UTI)
- Fatigue
- Muscle spasms
- Cold weather
- Sudden movements
- Negative mood (e.g., feeling sad or anxious)
- Constipation
- Full bladder
- Physical activity. Some people feel better when they exercise, and some people feel worse. Since exercise has so many health benefits, it is important to figure out what type of exercise works best for you.

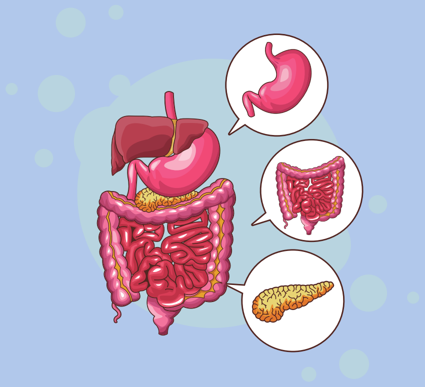

Please note that these factors do not have the same effect on all people with SCI. It can be very helpful for you and your doctor to know what factors make your pain worse because some of these factors can be prevented or treated.

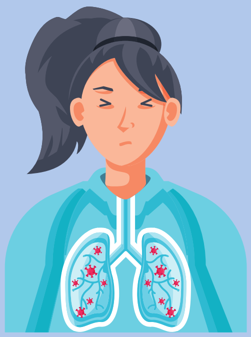

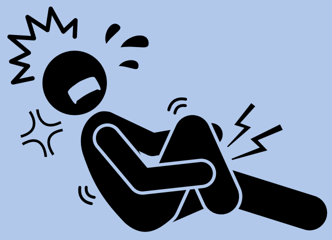

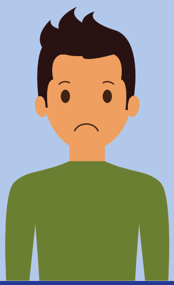

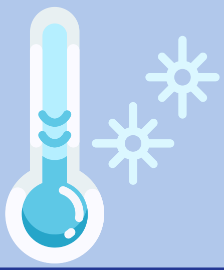

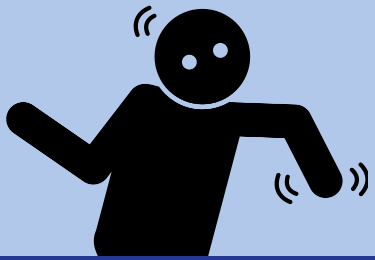

Evidence-based treatment

Pain research studies investigate how effective a treatment is in decreasing pain intensity compared to a placebo (non-effective) treatment. If the treatment is more effective than the placebo, it provides "evidence" that it works. Some studies also measure other important factors that are common in those who experience pain, for example, levels of depression, activity, or sleep.
When a specific treatment is not evidence-based, it means that no study has shown that the treatment "works" (e.g., reduces pain intensity, increases activity levels) better than a placebo. Even when the treatment is evidence-based and has worked for others, there is no guarantee that it will work for you.

First, we will discuss the common treatments that are often available and approaches that you can do yourself. Because there are still very few studies that have investigated treatments for neuropathic pain after SCI, there are treatments that do not have much evidence but have minimal or no risk or side effects. This includes many non-pharmacological treatments and is something you can discuss with your doctor.
After those sections, we will discuss treatments with an evidence base and the recommendations regarding those treatments.

Non-Pharmacological Approaches

Recent recommendations for optimal management of chronic pain after SCI often combine non-pharmacologic and pharmacologic methods. These combined pain management strategies may include patient education, cognitive behavioral therapy, self-management strategies, physical or relaxation exercises. The overall goal is to reduce pain, improve coping skills, and reduce pain interference with activity, sleep, and mood. In the following section we will discuss these options.

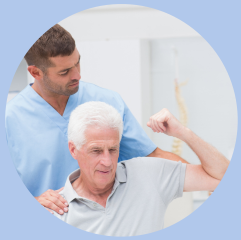
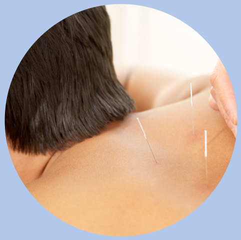
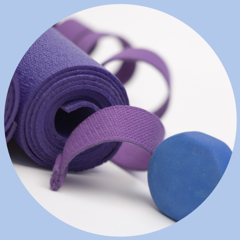
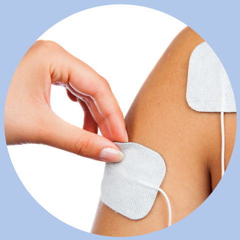
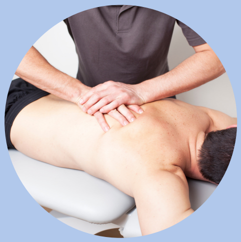
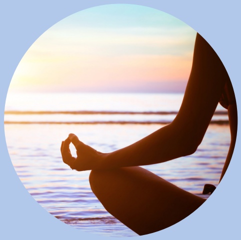
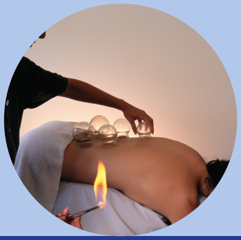
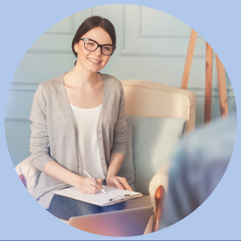

Electrical Stimulation/TENS

Transcutaneous Electrical Nerve Stimulation (TENS) is a device with electrode adhesive pads that sends low-voltage electrical currents to the nerves. TENS is often felt as a pricking/tingling non-painful sensation around the electrodes. TENS can be performed by a healthcare professional or at home after instructions with affordable kits that can be purchased or leased.

In several research studies, TENS has been shown to decrease pain in some people with SCI. For example, a 2013 study on the effect of low-frequency TENS treatment found a significant reduction in neuropathic pain compared to the placebo control group. This study enrolled 33 SCI patients who underwent 30 minutes of TENS daily for ten days.

Benefits

- Minimal side-effects
- No prescription needed
- Affordable
- Non-invasive
- Non-painful

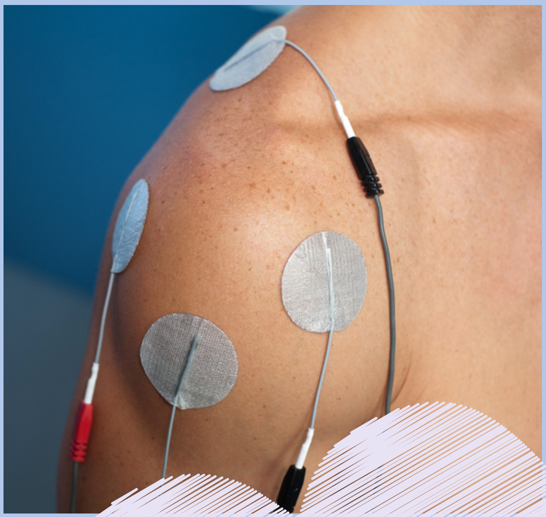

Healthcare provider: “ (TENS) may be underutilized... I can't say that it's extremely effective, but for some patients, it's been effective and it's probably worth trying."

A person with SCI: "using .. e-stim at my biceps area, that actually helped slightly...it.. kind of ... helps while you're doing it, but then after you're doing it, the pain returns.”

Transcutaneous electrical nerve stimulation (TENS). (n.d.). https://my.clevelandclinic.org/health/treatments/15840-transcutaneous-electrical-nerve-stimulation-tens

Exercise

The International Scientific SCI Exercise Guidelines (ISSEG) recommends 20 minutes of aerobic exercise 2 times per week (moderate to vigorous intensity) and 3 sets of strength exercises for each major muscle group 2 times per week (moderate to vigorous intensity).

Aerobic Exercises

- Hand cycling (arm ergometer)
- Swimming
- Rowing
- Cycling
- Wheelchair pushing
- Walking

*Try these exercises with free weights, resistance bands, or weighted cuffs to increase muscle resistance!

Strength Exercises

- Arms
  - Shoulder press: With the arms straight out making a T-shape, bend the elbows up to a 90° angle and have the palms facing forward. Straighten the elbows to lift the hands to the sky and bring them back down to 90°.
  - Arm Curls: With the arms at the sides and palms facing forward, bend the elbow to bring the hands toward the shoulder, then lower the hands back down.
  - Lateral Arm Raise: With the hands at the sides and palms facing the body, raise the arms straight out to make a T-shape, then lower the arms back down.
  - Shoulder shrugs
- Legs
  - Seated knee flexion: With the legs bent at a 90° angle, kick your foot out and try to straighten the knee as much as possible.
  - Hip flexion:
    - Can be performed standing or seated
    - Alternate the legs marching in place, try to lift the knee as high as you can.
  - Seated hip adduction:
    - With a ball placed between the knees, squeeze the ball by bringing the legs together.

Consult with your healthcare provider or physical therapist if you have any concerns or medical conditions

Full-Body home exercise program for spinal cord injury. (2021, March 02). https://www.flintrehab.com/home-exercise-program-for-spinal-cord-injury/

Some examples

Posterior rows

Start with comfortable hand placement and a secure position. When ready, pull resistance back, focusing on drawing the shoulder blades together and bringing the elbows slightly above 90°. Arms will stay alongside the torso to control the movement. Slowly lower the resistance and repeat the exercise with control.

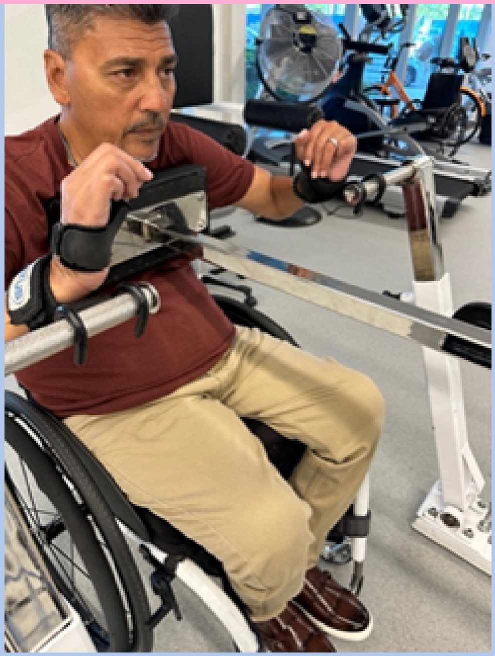

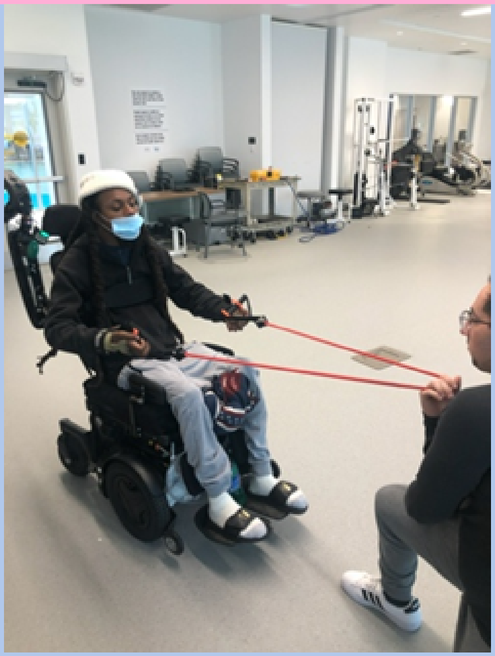

While some “traditional” workouts or movement patterns may not be appropriate for everyone based on their level of injury or individual skill level, there are many ways to modify or adapt each exercise to meet the needs of everyone. The use of resistance bands and/or adaptive gloves and other assistive equipment is a great way to learn and determine what exercises work best for you. Below are just a few options with potential modifications.

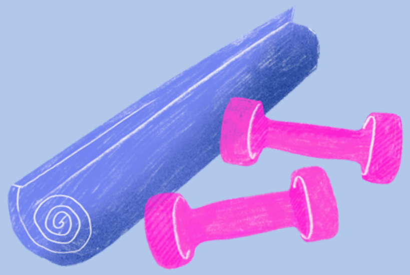

Bicep Curl

Starting in a secure position, hold a dumbbell in each arm along the sides of the body. With control and arms at near full extension, flex the elbows and bring the hands upward towards the shoulders. Lower with control and repeat.

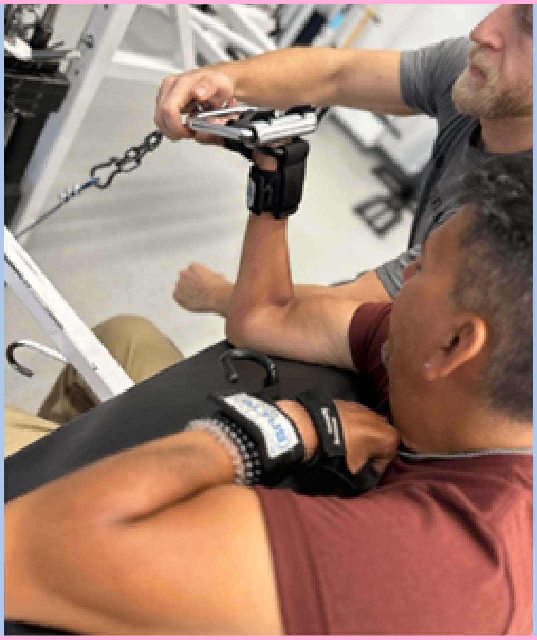

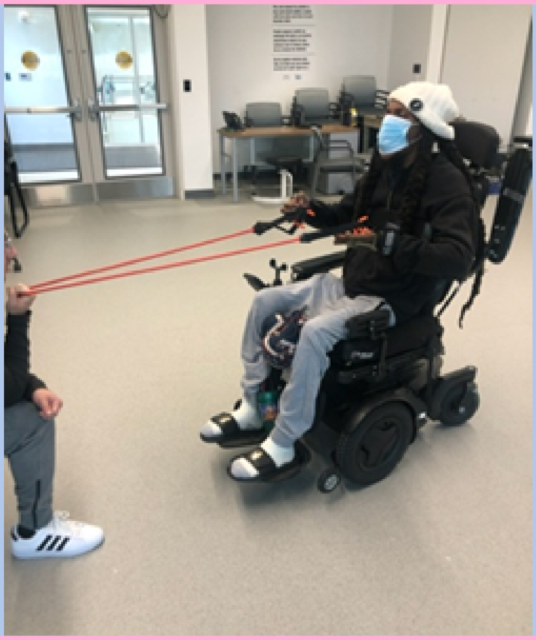

Lateral Raises

While holding the appropriate resistance in each hand, slowly raise both arms away from the midline to the level of the shoulders. The end position should resemble a “capital T” with the arms at either side.

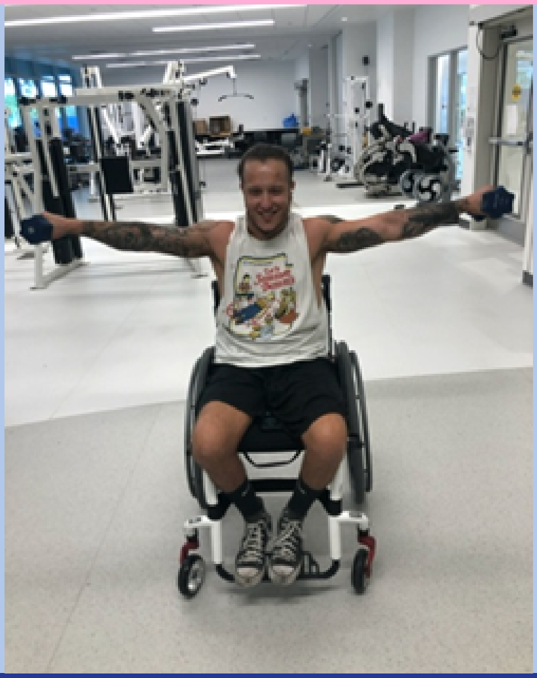

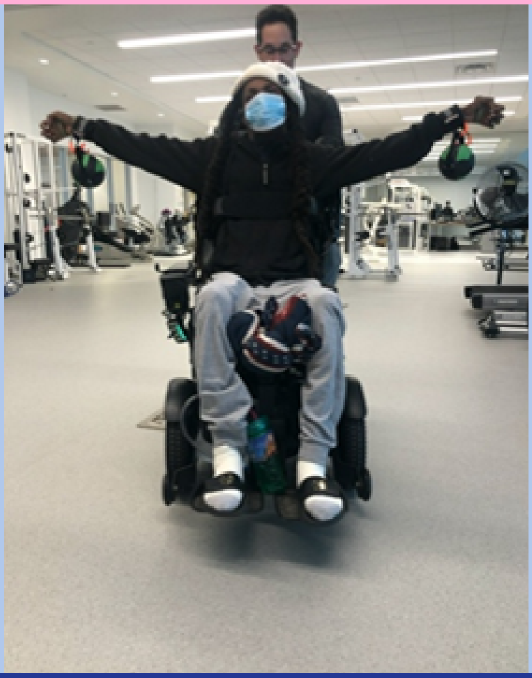

Arm Ergometer

The use of arm ergometers or gliders is a great way to build strength and cardiovascular fitness. If available, start in a secured position and place hands on the ergometer/glider handlebars. Initiate and continue the movements through the optimal range of motion to achieve the desired intensity.

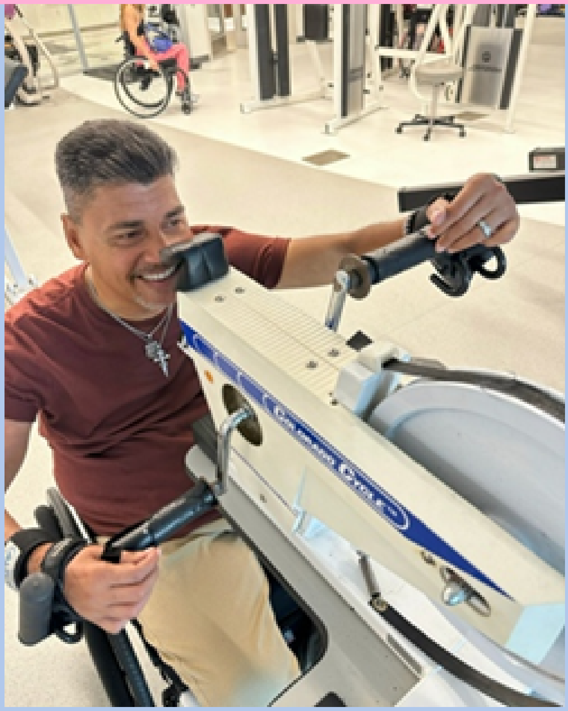

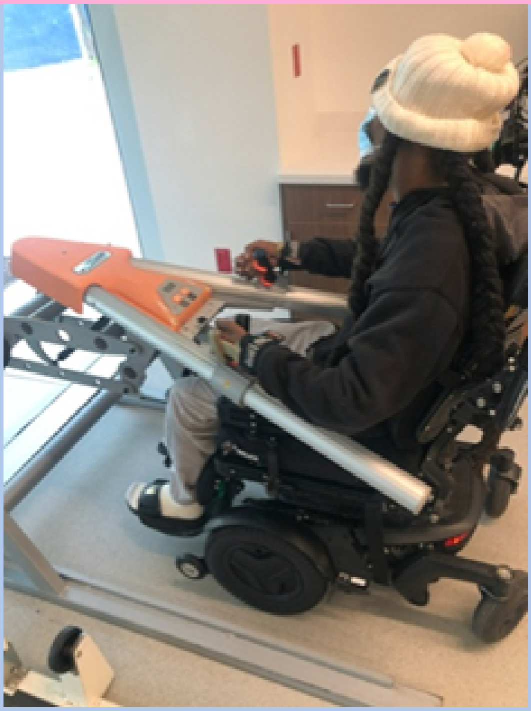

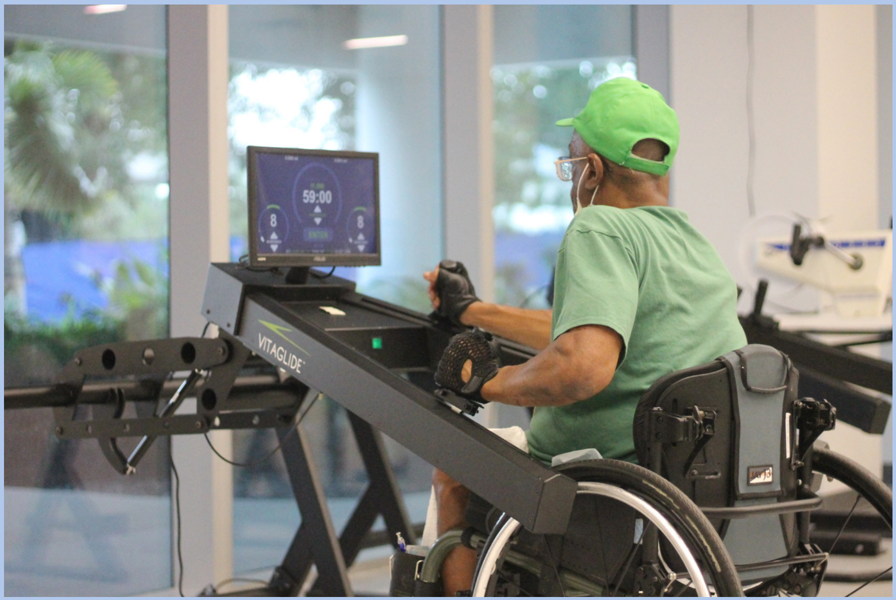

Some voices about exercise

“A lot of it has to do with exercise. I exercise every day."

"The physical exercise has a lot to do with how I deal with the pain. And I think I couldn't talk to people enough about that. You got to stay active. You got to exercise. And you got to be outside. Don't sit home and feel sorry for yourself. Just go out there and stay active.”

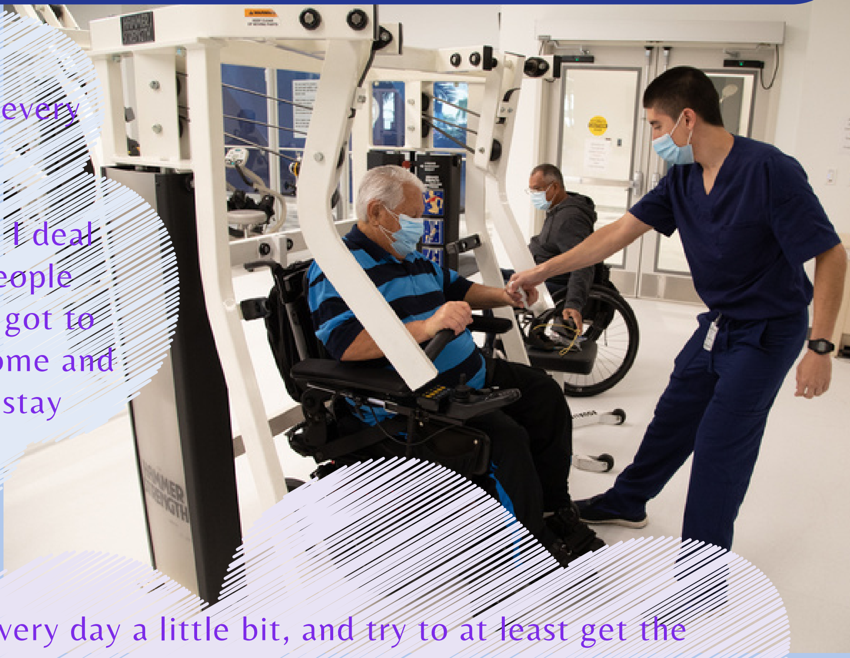

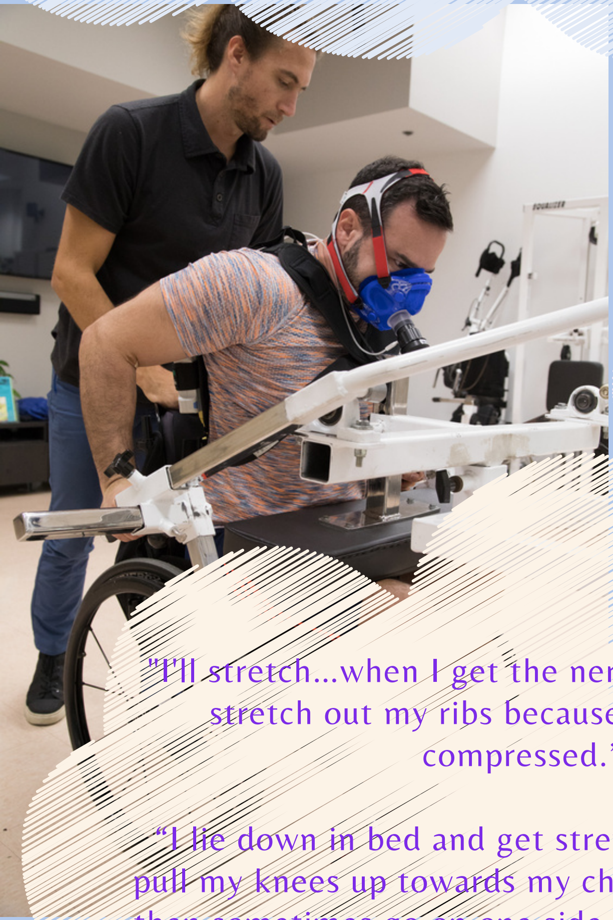

“every day a little bit, and try to at least get the pressure off of you. Uh, sometimes it can hurt worse, it depends on what exercise you do, and can hurt worse depending on what you do. Um, I've learned exercises that don't make it hurt worse, that actually makes it more comfortable.”

“Actually after using the standing table, both my legs would feel swollen...but.. the neuropathy would be gone.”

"I'll stretch...when I get the nerve at the pain... I'll stretch out my ribs because they feel so... compressed.”

“I lie down in bed and get stretched out and I can pull my knees up towards my chest and stretch, and then sometimes go on one side and go on the other side and I seem to get better.”

“I stretch myself... I change positions to... try to deal with it.”

Some voicecs about being active

"Stay busy, or try to stay busy."

"I like to go everywhere. So the pain, that's the last thing I try to let hinder me from being involved."

"You have to really push yourself to go out the door and even if you have nothing planned that day go for a roll..."

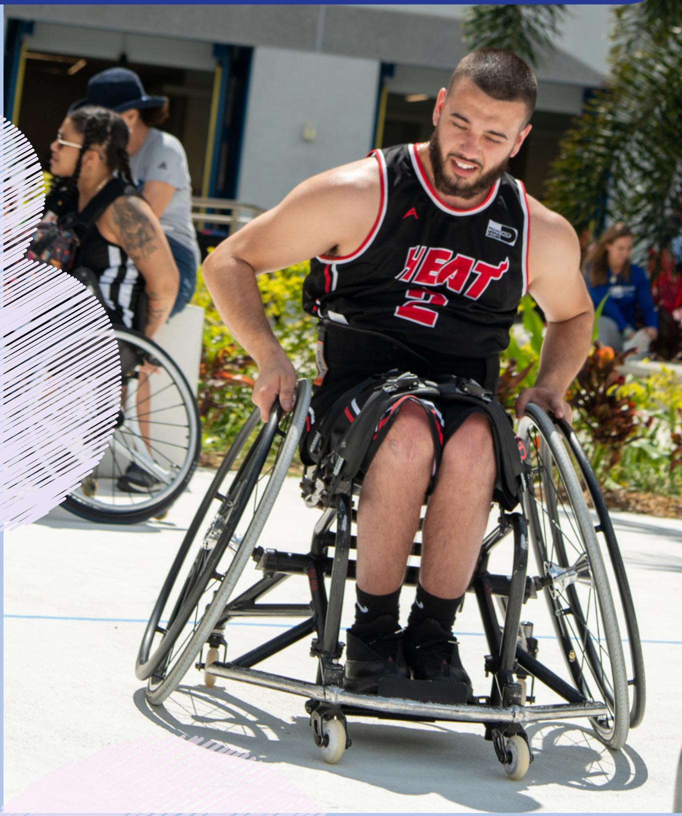

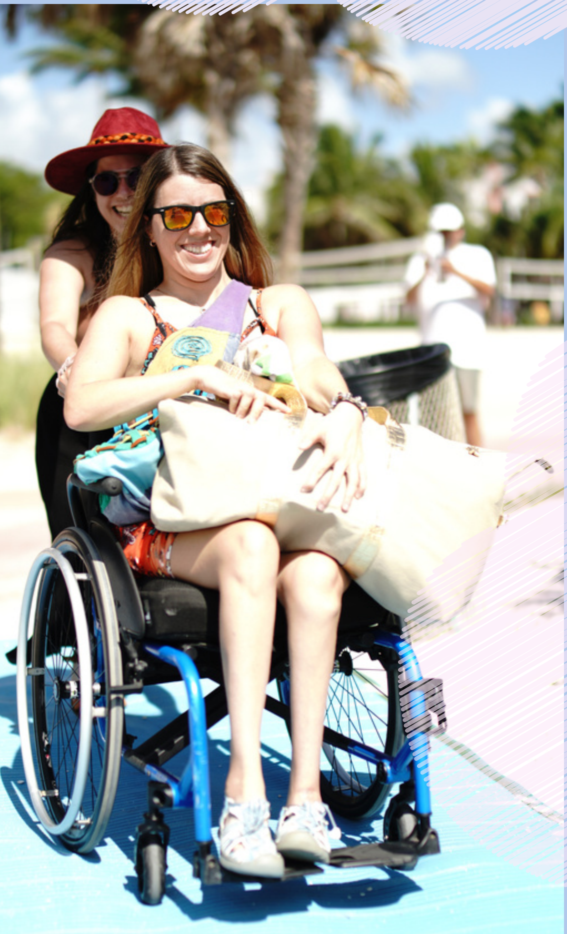

"...physically, it does help to be active... it's hard to do when you're already in a large amount of pain but,... kind of forcing yourself to just do things."

"And I stay very active, very visible, in the public eye and reach out and exchange ideas, and that's what's so important."

"Get out there and do things...stay active...don't let the pain dictate what you're going to do. You be proactive and go out and try to live a normal life."

Some voices about rest and physical therapy

Sometimes resting can help

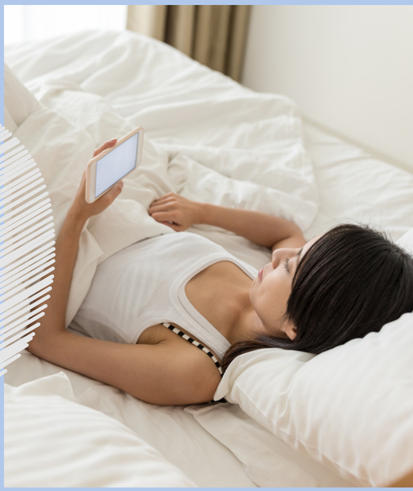

"The best pain relief is just to lie down and I lie on my side... for some reason, I've developed that habit. With all else fails, I just have to go lie down and then I get some relief."

"I think a lot of my pain comes from.. being in a wheelchair...all the time... and my legs get tight. I lie down in bed and stretch out, and cross my legs, either long way or both ways, left and right. That seems, to take, to take a lot of the pain away. Sometimes when I lie on my stomach flat on the bed, that seems to help"

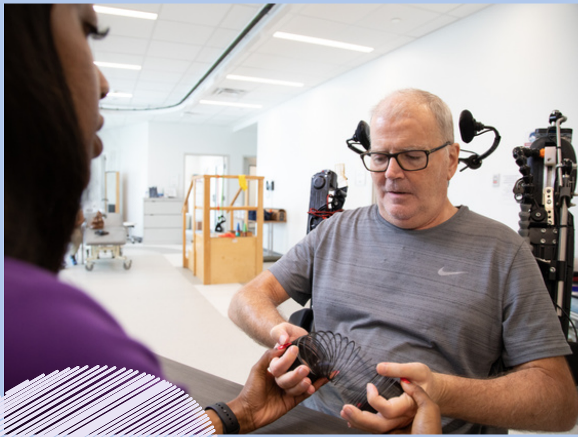

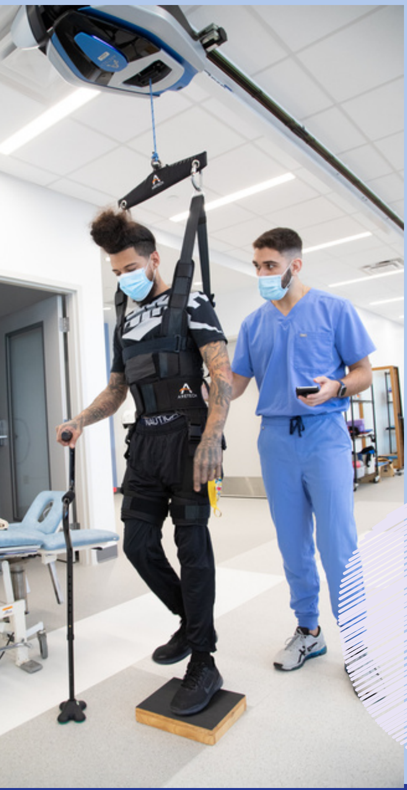

Physical Therapy

Healthcare providers can work closely with a physiotherapist or occupational therapist with the goal of improving the limitations of pain:

Healthcare provider: "So physical or occupational therapy... sometimes depending on ..... if a person is a higher level tetraplegic and their neuropathic pain is around a limb ... like their hand, and they're trying to use it for something it's, a lot of neuropathic pain in that area then...... I will work with the therapist to either do some type of mirror therapy, or desensitization therapy to allow the ... focus on function over just "get rid of the pain."

Some voices about massage and acupuncture

Massage and Acupuncture

"I gotta tell you a good massage makes a big difference."

"I have an automated massage chair, and the massages, they help relax the muscles a little bit. It reclines, and it does your legs, and it does your buttocks, and your back. And if I sit in that chair and recline for a while, then it would generally easy off"

"I've done acupuncture; that helps for about an hour..... the cold laser therapy, same thing. That'll help for an hour or so....."

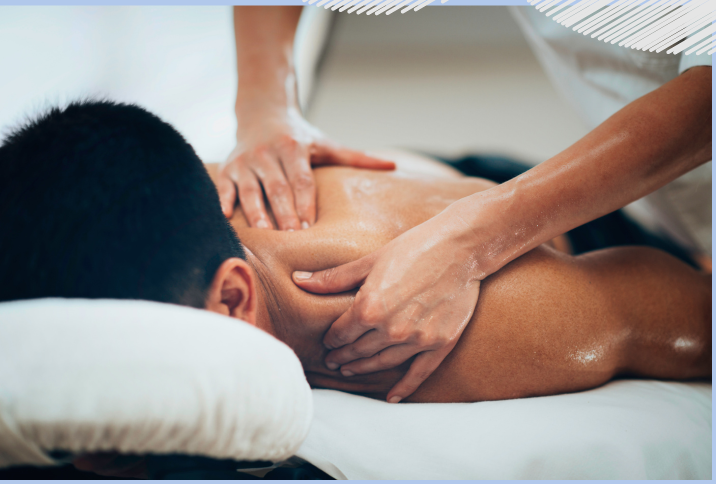

Healthcare provider: "People say they've had really dramatic relief with... massage, but it is a lot more expensive than the medications that we try."

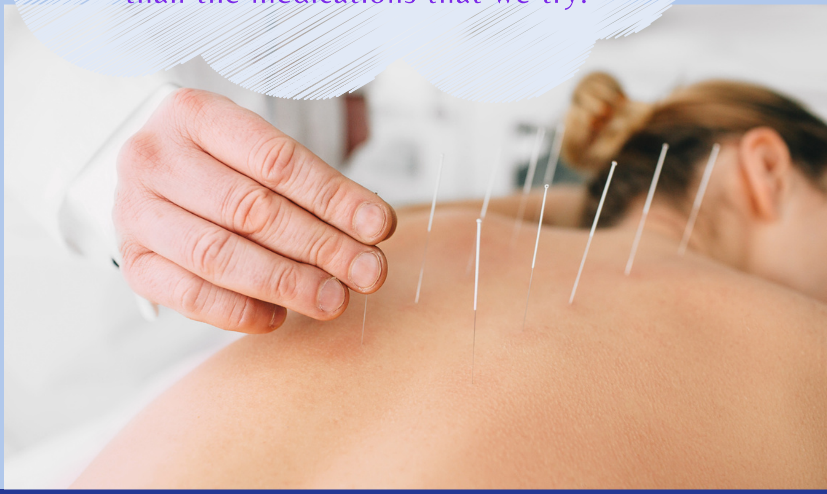

Mindfulness and meditation

Mindfulness is the art of being present. It involves going from one moment to the next with disciplined awareness. As John Kabat-Zinn (1994) describes, it is the "complete owning of each moment of your experience, good, bad, or ugly." In other words, mindfulness means being aware of your own thoughts, feelings, and bodily sensations, while also feeling a sense of calm and peace. This sense of calm and peace will in turn help you to achieve a clearer lens through which you can improve reactions, increase your ability to make conscious decisions and ultimately enhance your quality of life.

There are several ways you can become aware of such thoughts and feelings in order to shift your perspective. Meditation can assist in the mindfulness process by helping you to become more aware of your own thoughts, feelings, and physical sensations. This in turn may help to increase your sense of calm, allowing you to clearly identify your experience, which may result in your ability to make a clearer choice about how to manage your pain. Meditation can also take on several forms of practice, such as learning deep breathing or diaphragmatic breathing, practicing a body scan guided meditation, visualizing yourself being in a safe and comfortable place, or listening to a prerecorded meditation.

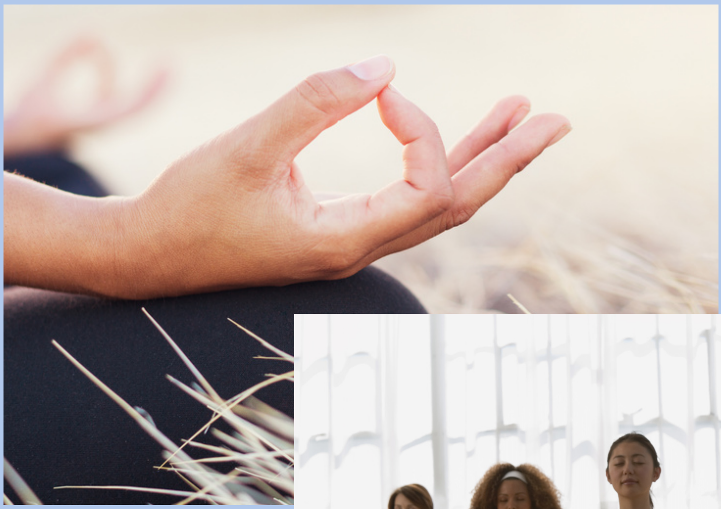

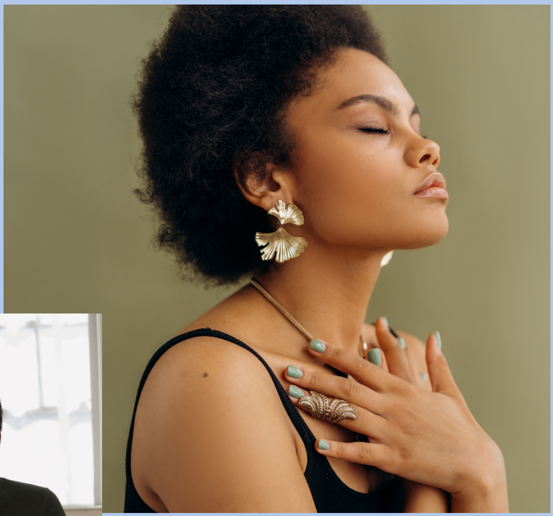

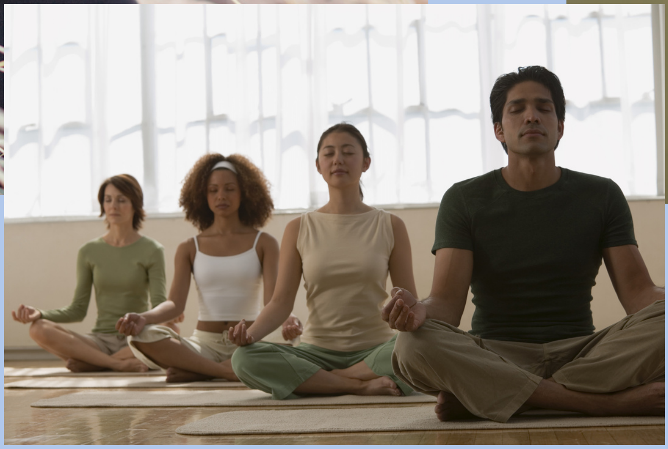

How to begin meditating?

Find a quiet space where you feel safe. You can lie down, be seated, or choose any position where you feel comfortable. Perhaps pick a time of day and place where you can focus on your practice. It may be challenging at first to learn to quiet your mind or even your surroundings and, therefore, the key is to start this practice slowly and to be consistent. You can even start by listening to guided meditations like the ones below:

- Insight Timer (iOS and Google Play, free, or $10/month or $60/year for premium): Insight Timer has over 55,000 free meditation tracks, many of which are geared toward beginners. It also has courses, such as Learn How to Meditate in Seven Days.
- UCLA Mindful (iOS and Google Play, free). Created by the UCLA Mindful Awareness Research Center and featuring recordings by Winston herself, this easy-to-use app has both basic meditations for beginners and wellness meditations geared toward people living with challenging health conditions. If you don't want to download the app, you can listen to a few of their free meditations on their website.
- Headspace (iOS and Google Play, $13/month or $70/year): Headspace comes highly recommended as a beginner app, thanks to its expansive library of guided meditations for just about every mood or goal. Plus, they have a lot of their own educational resources too, like this guide to different types of meditation.
- Calm (iOS and Google Play, $70/year): Calm is an all-around good starting point for guided meditation. You might like Calm if you prefer background nature sounds to silence.
- Healthy Minds Program (iOS and Google Play, free): Created by a nonprofit affiliated with the Center for Healthy Minds at the University of Wisconsin-Madison, the Healthy Minds Program app has meditations, exercises, and podcast-style lessons designed to build foundational mindfulness skills.
- Youtube.com: Search for guided meditations and many videos will appear. Choose the one that feels most calming. Some videos may be longer and others shorter. Begin slowly and work your way up to longer versions when possible.

Some voices about mind-body approaches

Yoga, relaxation and meditation!

"So I think ...meditation or relaxation.. it does help with the pain."

"I best handle my pain, I go into a lot of deep meditations. I exercise, do a little bit of yoga, and meditation together is a good combination. When I meditate, I get into my mind really good. It takes me out to a different world where I don't feel pain."

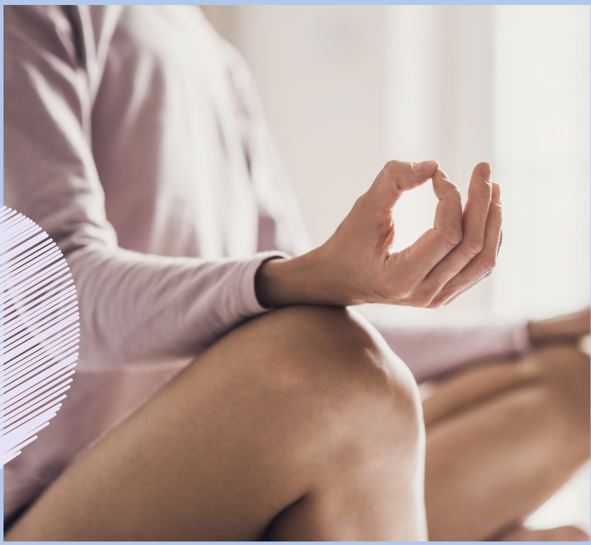

Mindfulness!

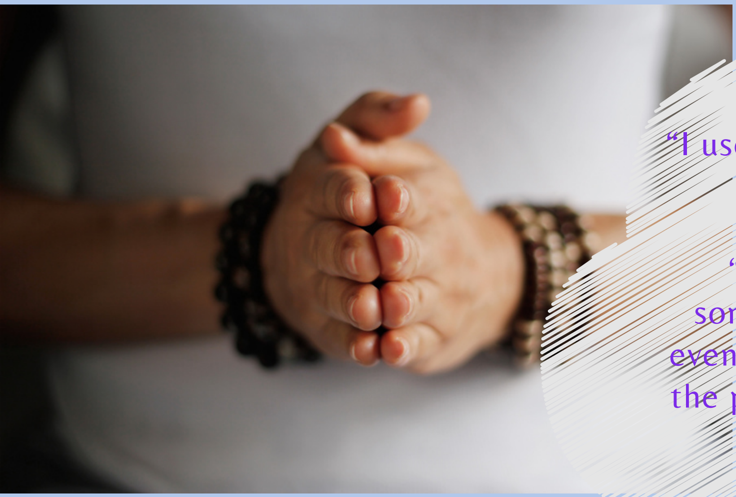

"I use my mind as a way to deter pain from breaking me down"

"...I'll place myself somewhere else, think about something else, take my mind off of the pain.... And even when I focus on the pain, move somewhat above... the pain... you're separating yourself from it, you're not right there, right there with it."

Some voices about mental strength

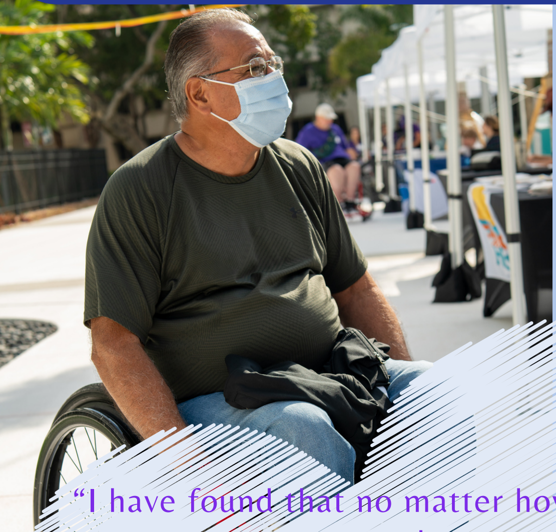

Mental Strength!

“You're in pain, sure, but if you look at it as a challenge to get through it... like a goal... it's your mission.. to not let it get to you.”

“And if you always have an attitude of gratitude and say to yourself when you start going down that bad path... Stop, take a deep breath... You need an attitude adjustment."

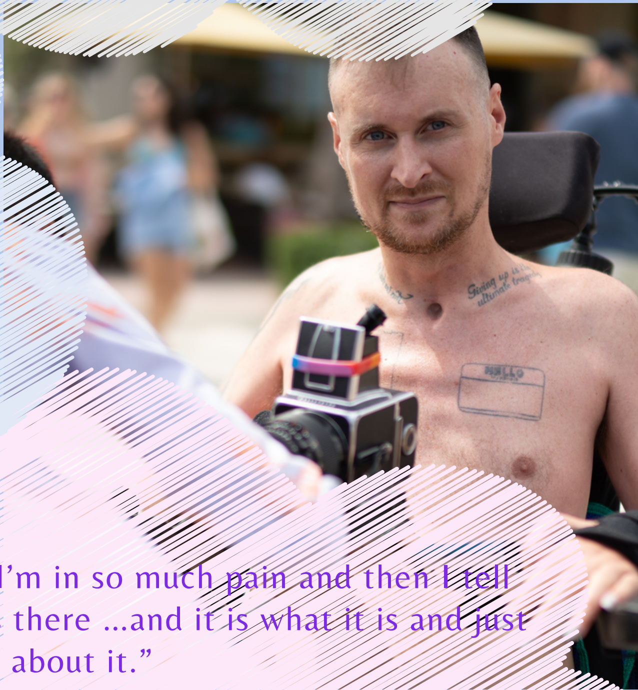

“I have found that no matter how much you hurt, you got to keep moving”

“I deal with it, I think, very well. In the 19 years, it's never stopped me from doing anything. I was an athlete, and I still am, prior to my injury, and I think that had a lot to do with my attitude towards the pain.”

“I try to live a normal life and don't let the pain, even though it's with me a lot, I don't let the pain uh, dictate how my life is led."

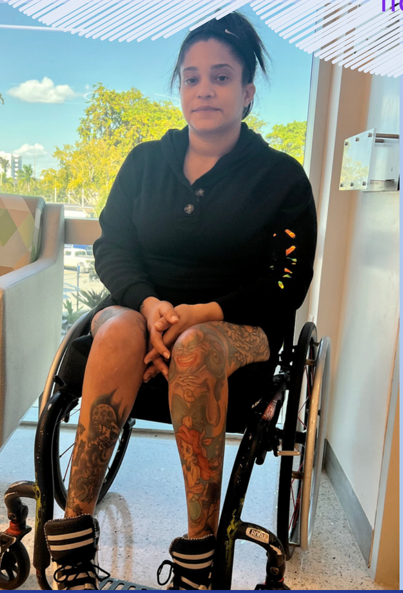

“I wake up and can’t believe I’m in so much pain and then I tell myself just forget about it, it’s there ...and it is what it is and just forget about it.”

“There are days when it’s really easily tolerable but it’s always there I just put it in the back of my mind after you know twenty-something years or so.”

"If ... I sit there and I think about the pain it will get worse.”

“I try to ignore it and move constantly and see if something works.”

Some voices about distraction and companionship

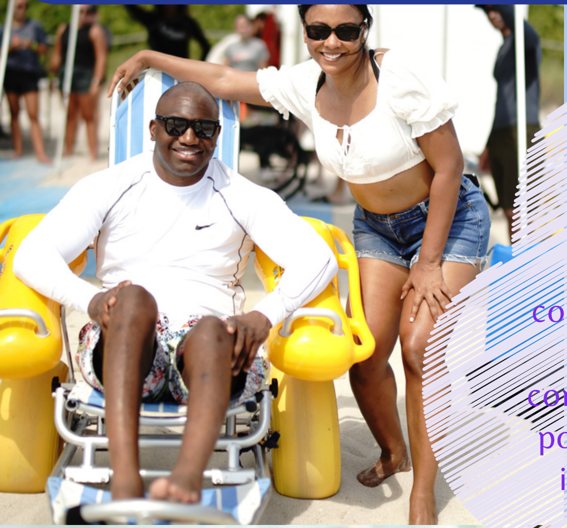

“Anything distracting. I mean, watching TV, sports, anything that gets your mind not on yourself.”

“Cause let's just say... I'm having a really good conversation or I'm watching a really good, like.... IMAX, I'm having a lot of fun at that moment, like, I'm not concentrating on the pain, so it's not that... serious to the point where I'm like, "Oh my gosh. I'm so miserable, I'm in pain." It's up to the point, like, "Okay, I'm having a good time."

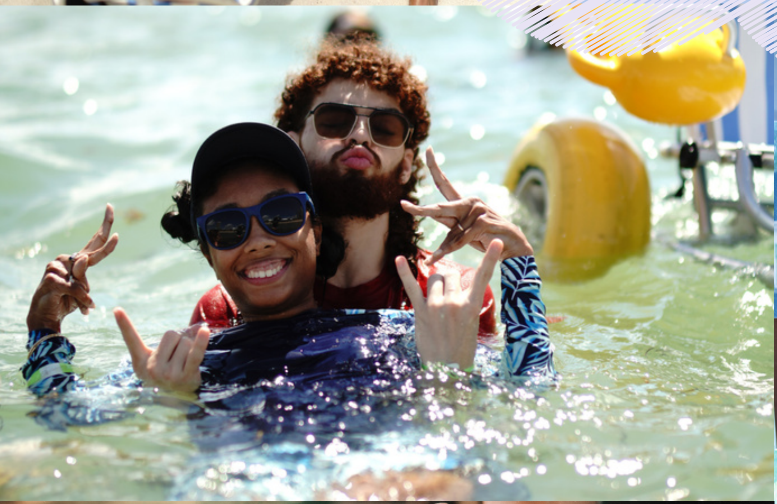

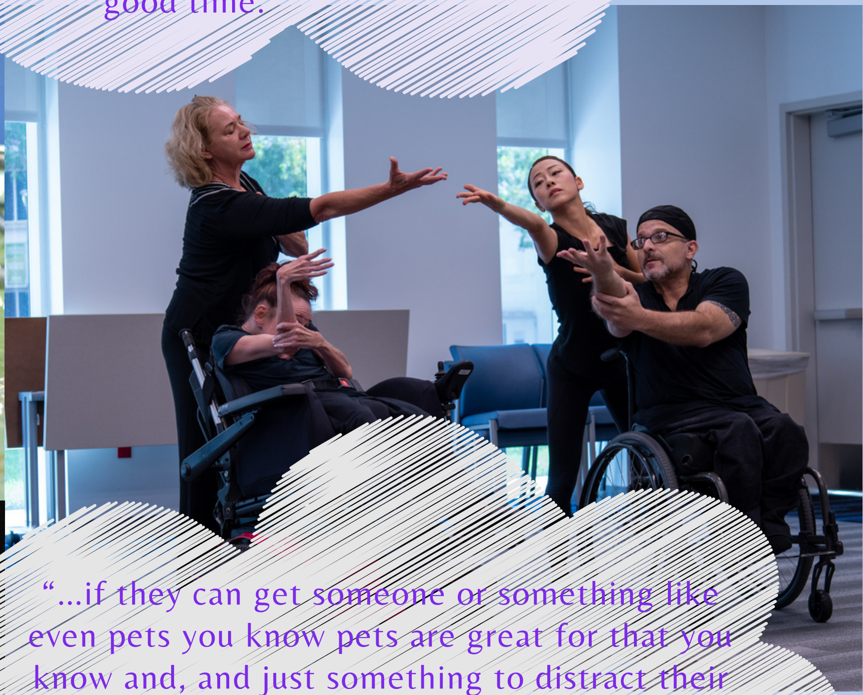

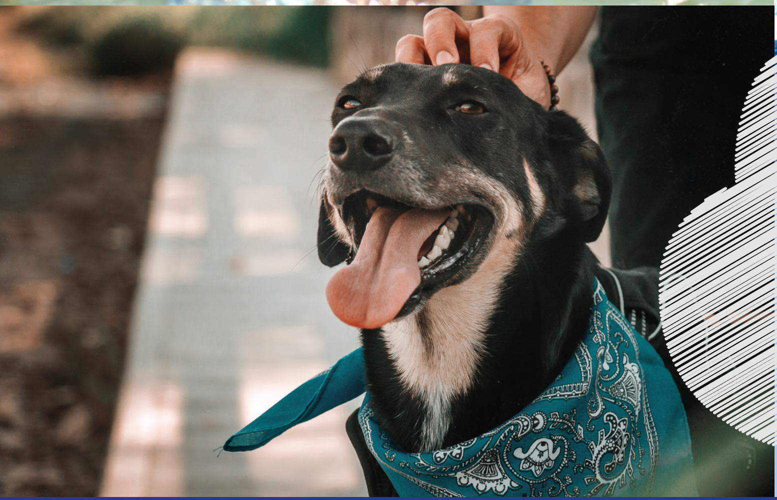

“...if they can get someone or something like even pets you know pets are great for that you know and, and just something to distract their mind from the pain you know whatever it could be spiritual, it could be you know a pet like I said or someone in the family but um that was one of my best um pain medicines was having companionship.”

Some voices about spirituality, hope and optimism

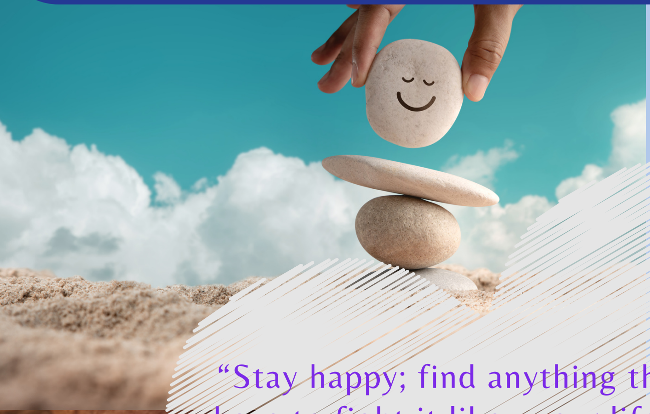

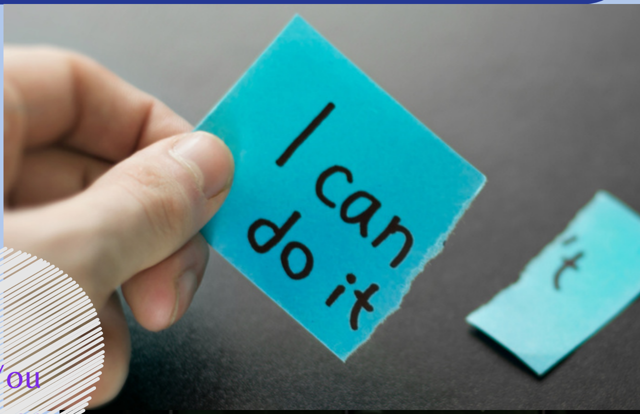

“Stay happy; find anything that makes you happy. You have to fight it like.. your life depends upon it because that pain, it will come and it will knock really hard."

“Pain is a challenge more than a problem.”

“..I try to pray every morning and that helps me get through with the pain... to deal with it.”

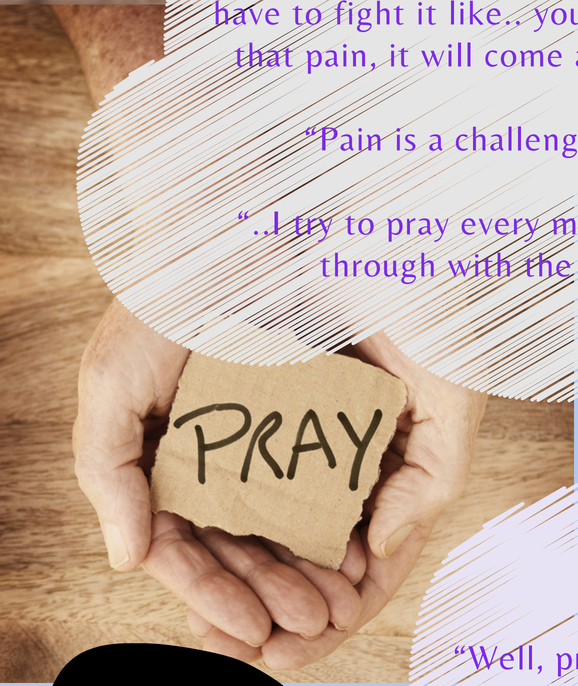

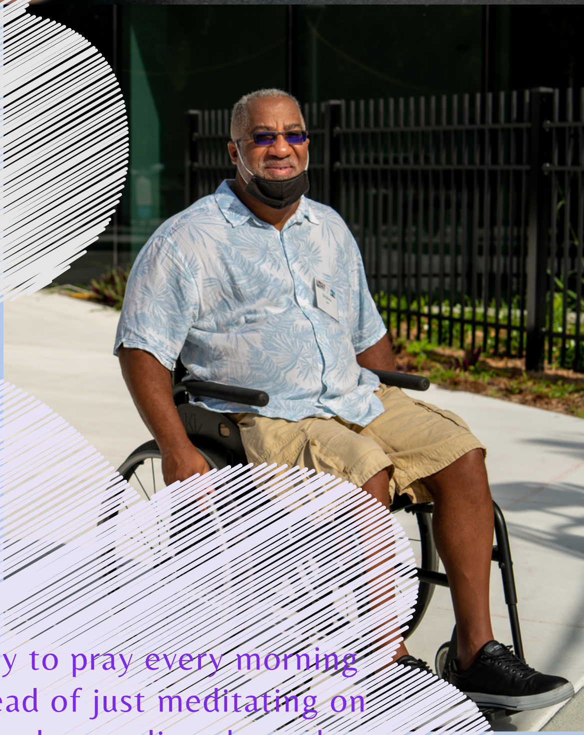

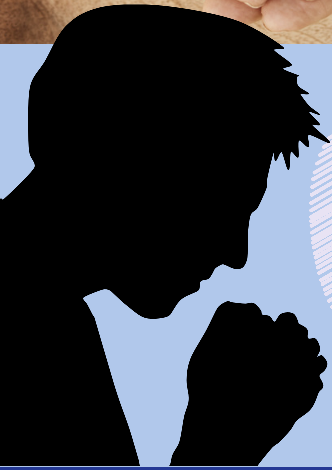

“Well, praying is the best thing. Uh, I try to pray every morning and that helps me... to deal with it. Instead of just meditating on the universe, I'm usually in prayer...and understanding that other people are in a lot worse situation than I am and being thankful for what I do have, bring this perspective to what has happened to me and believing that God has some purpose for this in my life. It's not just some random act of the cosmos. There is some purpose one day that I'll be able to realize.”

Some voices abour learning, Planning, and Anticipating

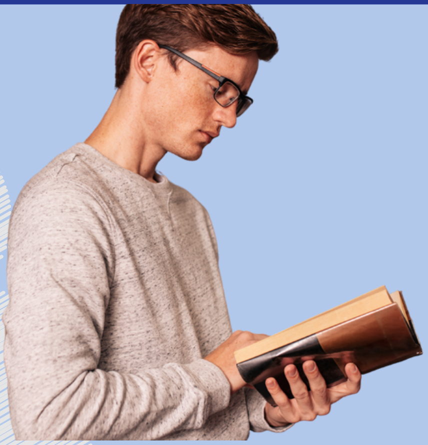

"Pain to me, is... something that an individual has to educate themselves on how to tolerate. And, uh, they can only educate themselves if the material is presented, or if they're allowed access to educational materials in regards to how to cope with their pain."

"Everyday is a challenge... a learning experience, for me. It's been a learning experience. And um, it teaches not to take things for granted."

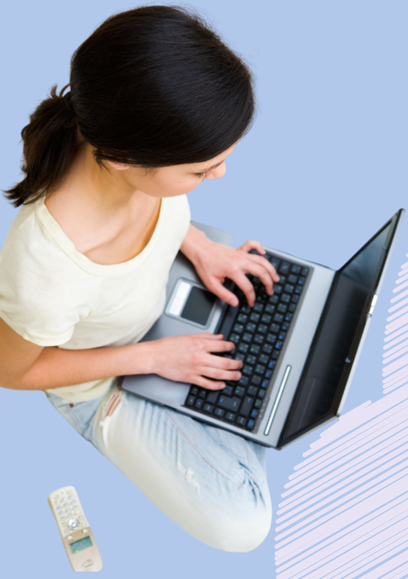

"How do I turn so that this side won't hurt anymore? This side is hurting, let me turn, how do I stretch my foot? Let me see how I can wiggle my toe...I'm always learning and then once my body starts to feel aching or I start to, Okay, let me learn to calm down, relax, give my body a break, go back to ... To square one in a couple minutes, or in an hour, or maybe tomorrow." You learn to have patience and you learn to analyze everything you do And if I can't focus on something, I leave it alone, I don't torment myself with it. I leave it alone and I start another day when I'm more focused."

"Um, just, uh, I don't overdo it. I learn that, not to overdo it, cause that hurts."

Pain Acceptance - not resignation!

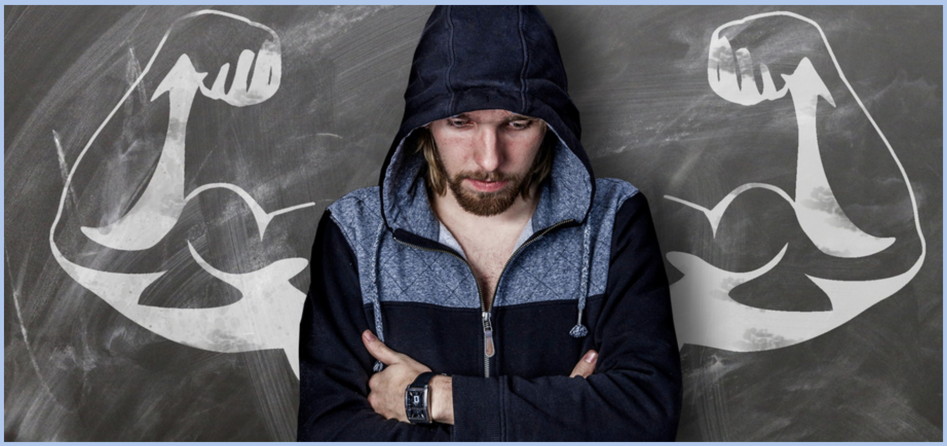

Many individuals living with spinal cord injuries struggle with the idea of accepting their pain and/or disability. The thought of accepting something perceived as negative or unwelcoming may seem counterproductive, or perhaps it may feel as if you are just giving up because you believe there is nothing else that can be done about it, yet this is not what defines acceptance. Unlike resignation, which is when you have convinced yourself that nothing more can be done about your situation, acceptance means you recognize the reality of your situation while still acknowledging your that you have control. Acceptance is not a passive state like resignation, where you simply "throw in the towel," but rather it is a state where you can deliberately commit to acknowledging that your situation has changed. You also acknowledge that your situation will no longer have power over you. Refusing to accept your new situation, you may end up feeling trapped in a state of emotional suffering. Through acceptance, you become capable of creating change and will no longer feel trapped, whereas, through resignation, you remain a passive bystander believing that the odds are always stacked against you.

"Won't let the pain dictate how I'm living my life... it's something I live with. You have to learn how to deal with it or it'll beat you every time."

"If you don't face it, and it comes out years later, you're really gonna be in trouble. I faced mine"

"it's at this point where I feel like it's something I'm going to have to live with for the rest of my life."

"Some of the medications that... I've been prescribed that it hasn't ...alleviated the pain. I kind of feel like, Man, well, I better, just get used to it because it might take a while or they may never actually find something to help cure this pain".

Recommended Treatments

Despite that many medications have been effective in other chronic pain conditions, about 70 - 80% of people with SCI continue to experience pain a long time after their injury. Treatment recommendations have been published to help healthcare providers make the best treatment decisions. However, sometimes your doctor may want try something that does not have a strong evidence base for SCI but has been safe and useful for some other chronic pain populations.

The recommendations below were made by an expert group and based on both scientific evidence, and factors like side-effects, clinical experience, and effectiveness of the treatment in other chronic pain populations. The Figure below summarizes the pharmacological and non-pharmacological treatments that this group recommended in their suggested order. First-line treatments will be recommended first and so on. We will discuss some of these recommendations for these treatments used to treat neuropathic pain after SCI, starting with those that have the strongest evidence base.

Please note that the non-pharmacological approaches that you learned about in the previous sections can be used in combination with medications. The combination of treatments is something that you should discuss with your doctor to see what is best available for you.

First Line

- Pregabalin (Lyrica)
- Gabapentin (Neurontin)
- Amitriptyline (Elavil)
- Duloxetine (Cymbalta)

Other options

- Oxacarbazepine (Trileptal, Oxtellar XR)
- Tramadol (Ultram, Ultracet, Ultradol, ConZip)
- Lamotrigine (Lamictal)

- Botulinum toxin A
- Transcranial direct current stimulation
- Combined visual illusion and transcranial direct current stimulation
- Spinal Cord Stimulator

- Oxycodone (opioid)
- Cannabinoids
- Transcutaneous electrical nerve stimulation (TENS)
- The dorsal root entry zone (DREZ) procedure*

*given its invasiveness as a treatment and significant risk of side effects, should only be considered as a last resort if it is used at all.

Guy SD, Mehta S, Casalino A, Côté I, Kras-Dupuis A, Moulin DE, et al. The CanPain SCI Clinical Practice Guidelines for Rehabilitation Management of Neuropathic Pain after Spinal Cord: Recommendations for treatment. Spinal Cord. 2016;54(S1):S14–23.

First Line treatments

These treatments have the most evidence for treating neuropathic pain after SCI. However, because the pain experience differs for each person, not every medication works for every patient. And even if it is the 'best' treatment and it doesn't work for you, something else likely will. There are also newer drugs than the ones below that have similar effects that may be better for you. When starting a new medication, it is important to start slowly to ensure you tolerate the medication. Because neuropathic pain is often difficult to treat, it is important to use different or multiple methods to deal with it. You can sometimes get a 10% improvement here, 10% here, and 10% there, which then makes a 30% improvement. Try to develop a good relationship with your doctor so that you can discuss different treatments and combinations or if you want to stop taking a drug because stopping too abruptly may cause severe side effects.

Pregabalin (Lyrica)

Will it work? Several research studies that used pregabalin to treat neuropathic pain showed that it significantly reduced the intensity of pain. Most studies used flexible doses between 150-600 mg per day. It is important to keep in mind that even the most effective medication does not work for everyone.

How it works: Pregabalin is an anticonvulsant and can decrease the hypersensitivity of nerve cells by reducing the number of excitatory neurotransmitters that the neurons release.

Potential side effects: Drowsiness, dizziness, dry mouth, limb swelling, blurred vision, weight gain (around 4%), and abnormal thinking (difficulty with concentration or attention).

Amitriptyline (Elavil)

Will it work? Amitriptyline is another less common medication that can be used to treat Neuropathic pain after SCI. Studies have had mixed results, but overall, the studies suggest that amitriptyline could be beneficial. Studies used doses between 25 and 150 mg per day.

How it works: Amitriptyline is a tricyclic antidepressant. It has many effects, but the reduction of neuropathic pain is thought to be due to increasing the effectiveness of the body's own pain inhibitory system by keeping the brain and spinal cord levels of the neurotransmitters noradrenaline and serotonin elevated.

Potential side effects: Drowsiness, dry mouth, constipation, blurred vision, and low blood pressure. There is also a potential of memory impairment in older subjects.

Gabapentin (Neurontin)

Will it work? Gabapentin is used as an alternative to pregabalin. Some studies in people with SCI found no effect while others showed a significant reduction of neuropathic pain using doses between 1800 and 3600 mg per day.

How it works: Like pregabalin, gabapentin is an anticonvulsant and can reduce the number of excitatory neurotransmitters that neurons release.

Potential side effects: Drowsiness, dizziness, tiredness, blurry/ double vision, limb swelling, weight gain (around 4%), nausea, vomiting, trouble speaking, headache, tremors, and difficulty with coordination and concentration.

Duloxetine (Cymbalta)

Will it work? The typical starting dose is 60 mg a day, which may be increased to twice a day.
How it works: Duloxetine is another antidepressant known as a serotonin-norepinephrine reuptake inhibitor (SNRI). Duloxetine has multiple functions, including reducing neuropathic pain by increasing serotonin and norepinephrine concentrations in the synapse of serotonergic and noradrenergic neurons in the descending spinal pathway on the dorsal horn of the body.
Potential side effects: Dry mouth, constipation, drowsiness, dizziness, nausea, vomiting, fatigue, increased sweating, loss of appetite, and sexual dysfunction.

Other pharmacological options

Oxcarbazepine (Trileptal, Oxtellar XR)

Will it work? Oxcarbazepine is an anticonvulsant and can be used to treat neuropathic pain after SCI. A study in people with SCI pain showed that this medication reduced electrical, burning, and pricking pain, numbness, allodynia, and pressure analgesia. In this study, Oxcarbazepine was started at 150 mg twice daily and increased up to 300 mg twice daily depending on the response.
How it works: Carbamazepine is another medication where the precise mechanism is not completely known. However, some studies show they reduce hyperactivity in the brain and the spinal cord.
Potential side effects: Oxcarbazepine are dizziness, drowsiness, tiredness, balance or coordination problems, nausea, vomiting, tremors, double vision, and skin rashes.

Tramadol (Ultram, Ultracet, Ultram ER, Ultradol, ConZip)

Will it work? Tramadol can be used to treat neuropathic pain, meaning that it could be tried after "first-line" medications have not provided satisfactory pain relief. One study found a significant benefit for neuropathic pain after SCI. The maximum dose is 400mg per day.
How it works: Tramadol is an opioid-like medication with many different actions, not all known. One of its actions is to increase pain inhibition.
Potential side effects: Drowsiness, dizziness, tiredness, headache, itching, stomach pain, nausea, vomiting, and constipation. Tramadol is a controlled substance in the US and there is a risk of addiction.

Lamotrigine (Lamictal)

Will it work? Lamotrigine is an anticonvulsant and another option for neuropathic pain in individuals with incomplete SCI. One study found a significant benefit for neuropathic pain in people with incomplete injuries. The maximum dose of lamotrigine in the study was 400 mg per day.
How it works: The primary action of lamotrigine is decreasing hypersensitivity in neurons by reducing the number of open ion channels.
Potential side effects: Dizziness, drowsiness, headache, blurred vision, double vision, skin rash, tremors, loss of coordination, dry mouth, nausea, sore throat, runny nose, back pain, and insomnia.

Prescription opioids are legal in the United States; however, some synthetic opioids such as fentanyl are sometimes used illegally or abused in the US. The potential abuse of opioids was documented from 1999 to 2019, when almost 247,000 people died in the United States from overdoses involving prescription opioids. The number of overdoses and deaths from prescription opioids has gradually increased over the years and quadrupled from 1999 to 2019. This has led several states to enact State Drug prescription Monitoring Programs and the consequence is that narcotic prescriptions and quantities dispensed are now more tightly regulated.

Oxycodone (Oxy, Oxycontin, Oxyfast, Roxycontin)

Will it work?

Oxycodone is an "other option" for neuropathic pain. Even though one study found a significant reduction of neuropathic pain in persons with SCI, the side effects and addiction risks are high.

How it works: Oxycodone is a potent opioid that increases pain inhibition.

Potential side effects: respiratory depression, dizziness, drowsiness, headache, constipation, stomach pain, nausea, vomiting, itching, red eyes, and flushing.

The potential for adverse effects and other problems of opioids resulted in a weak strength of recommendation.

Doctors may prescribe opioids to alleviate both chronic and acute pain. There are several types of prescription opioids that can be used to treat moderate-to-severe pain following a spinal cord injury, surgery, or other health condition requiring pain relief. With opioids, there are risks of addiction, abuse, and overdose, as well as several side effects. Many of the side effects associated with long-term opioid therapy can negatively impact an individual's quality of life. Some side effects include opioid-induced constipation, increased pain sensitivity, lower levels of male sex hormones, sleep disturbance, and physical dependence. Patients may also build a tolerance to their prescribed dosage of opioids, causing the medication to be less effective. Opioids, when used judiciously, can be an effective and important tool for chronic pain treatment, but their use requires close monitoring and close communication between the doctor and the patient.

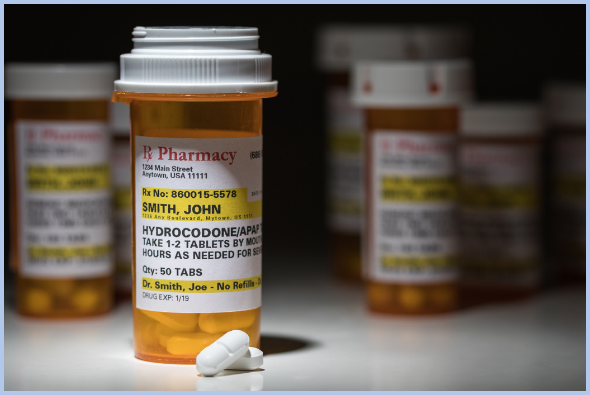

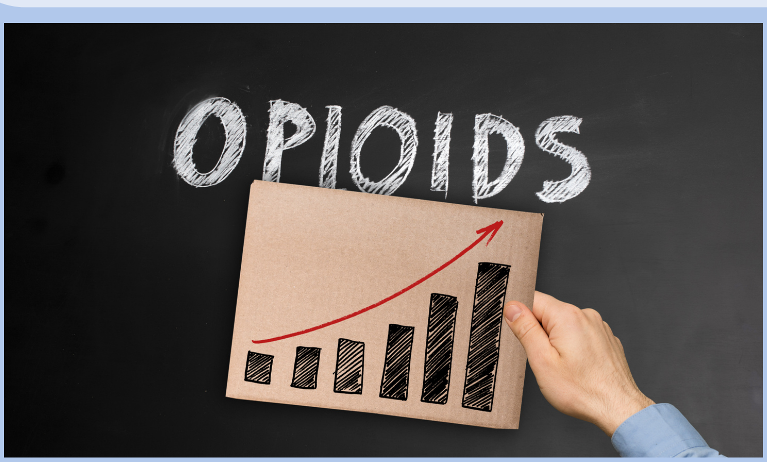

- Wide-ranging online data for epidemiologic research (WONDER). Atlanta, GA: CDC, National Center for Health Statistics; 2020. Available at http://wonder.cdc.gov.
- CDC. CDC Works 24/7 [Internet]. Centers for Disease Control and Prevention. 2019 [cited 2019 Jul 1]. Available from: https://www.cdc.gov/index.htm
- Services USD of H and H. HHS.gov [Internet]. HHS.gov. [cited 2019 Jul 1]. Available from: https://www.hhs.gov/

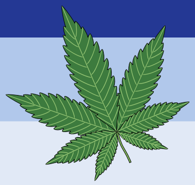

Cannabinoids

Cannabinoids have the potential to affect multiple central nervous system targets, thus more recently it has been of interest for the treatment of pain and other neurological disorders. Studies have found varying results in the treatment of SCI-related neuropathic pain and more investigative research is needed. However, individuals with SCI have reported lower pain levels while using cannabinoids. One study found that the intensity of SCI-related neuropathic pain was significantly lower after vaporized cannabis (2.9%, or 6.7% THC) compared to placebo. Another study did not find differences between synthetic THC and placebo.

If you are being treated with opiates in the U.S., please consult with your Pain Management doctor before you try cannabis. Opiate treatment is done under a contract that stipulates what is and is not allowed, and the use of cannabis may be an issue.

Different versions of cannabis have been used to treat human diseases for a very long time. However, the relatively recent and better understanding of the cannabinoids themselves, in combination with changing laws around cannabis use, have resulted in an increased interest in cannabinoid treatments. These types of treatments are of interest for neurological disorders (including pain) because of the discovery of cannabinoid potential to affect multiple central nervous system targets. However, the exact mechanisms by which they might relieve pain are not known. There is also not much known regarding the long-term side effects of medical cannabis use.

The European Federation consensus paper is an excellent source of information that provides expert recommendations for European healthcare professionals regarding the appropriate use of medical cannabis (plants and plant material) and cannabis-based medicines (cannabis extracts with defined and standardized THC and THC/CBD content) as part of a multidisciplinary approach to pain management. These guidelines may need modification in the US since there are no current guidelines/recommendations besides the FDA-approved: dronabinol (synthetic THC), nabilone (THC analog) to treat AIDS-associated nausea; and CBD to treat severe childhood epilepsy.

This review highlights several important factors to consider when used to manage pain: 1. Patients need to be carefully selected and supervised. 2. Contraindications include, for example, hypersensitivity to cannabis-based medicines, mental disorders, substance abuse, seizures, severe heart problems, and the use of high doses of opioids or sedatives (benzodiazepines). 3. Lower doses are needed in elderly patients and individuals with liver or kidney problems. 4. Caution with respect to work and driving. "There is insufficient evidence as to whether medical cannabis and cannabis-based medicines differ in their efficacy, tolerability, and safety. There is no evidence available that the different formulations of medical cannabis, such as cannabis oil, are more effective or safer than dried medical cannabis”.

There are some non-pharmacological treatments that are supported by some scientific evidence. These options may be considered for reducing neuropathic pain after SCI. Please note that these treatments have primarily been used in research studies and may not be available to you but you can ask about these treatments when you consult with your doctor.

Botulinum toxin A

Botulinum toxin A injections are widely used for reducing spasticity because this compound decreases muscle activation. When people who experience neuropathic pain also have spasticity in the same area, Botulinum toxin A has been used for below-level neuropathic pain with injection in an area with maximal pain.

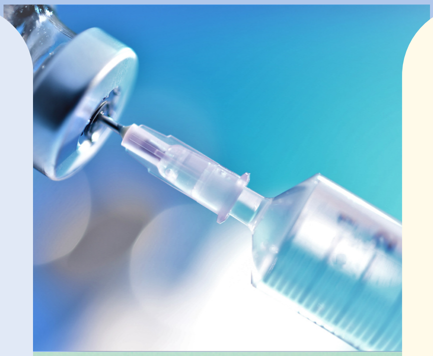

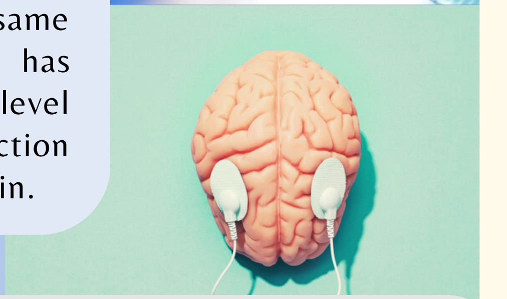

Transcranial direct current stimulation (tDCS)

Transcranial direct current stimulation uses constant, low direct current delivered via electrodes on the head and is a way to influence nerve signals and pain. People with neuropathic pain after SCI may experience a reduction in pain, specifically in pains that are continuous or stabbing. Side effects may include skin irritation and visual perception of a brief flash of light when the electrode is placed near the eye.

Combined visual illusion and transcranial direct current stimulation (tDCS)

Transcranial direct current stimulation has also been combined with visual illusion. Visual illusion is a way to trick the brain into seeing something that is not really there for example by using mirrors. A study found a significant reduction in pain intensity after a combined visual illusion and tDCS in people with neuropathic pain. Side effects include mild headache and fatigue.

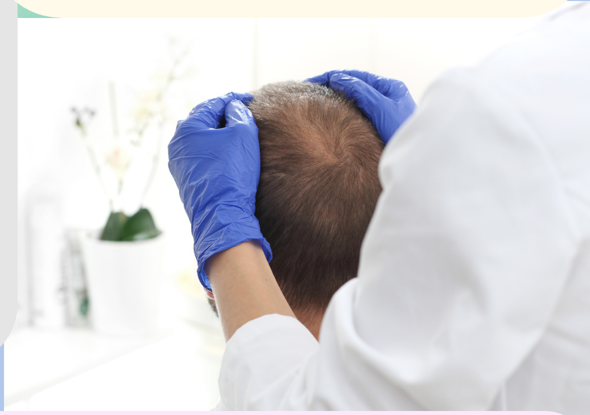

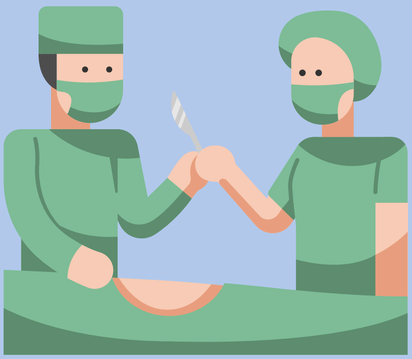

The dorsal root entry zone (DREZ) procedure

This approach is used for exceptional circumstances and as a last resort due to its invasive nature. DREZ procedure involves a neurosurgeon creating an opening in the lamina of the spine to identify an area in the dorsal surface (back) of the spinal cord where it has been damaged to silence hyperactive nerve cells by creating lesions in the dorsum of the spinal cord. The goal is to decrease the number of pain signals. This has been more successful in lower thoracic spinal cord injuries, but carries a risk of losing more sensation.

Thoughts about opioid medications - people with SCI

"I've used medications for the first seven, eight years, then that's when I asked to get different help, because I didn't like what they were doing to me. It felt like I was brain dead. They had me on Fentanyl... Oxycontin, Roxicet. Everything you can think of, I've been on it."

"I've tried many, many... forms of.. pain meds and whatnot; and I'm still on... pain meds. But one of the things that I noticed that everything that, my capabilities were limited and what I could do. And when the pain meds wear off, I become more irritable. I become short tempered. It has affected my social life."

"I used to take half the amount or a quarter the amount before to get some relief now three, four times the same medicine is barely enough. So I don't know if that's because I'm developing more tolerance to the medicine or because my pain actually increased."

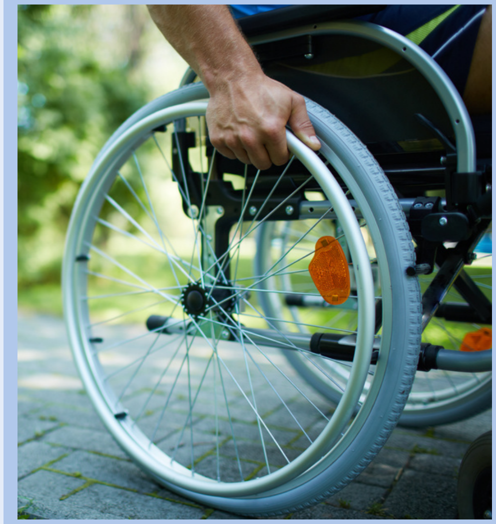

"They put me on methadone and it worked great. In fact, methadone is probably the best thing that I've had for all my pain. It's longer lasting thing, longer acting they're too, too quick to just drug you and continue to drug you."

"I was taking Oxycontin twice a day so I would always be sleeping, I would always be on drugs and it affected my life because my children they didn't even want to see me, I didn't care about anybody just the pain to go away I just want to be, take the pain away so they would give me the drug and that was it."

Thoughts about opioids – significant others and family members

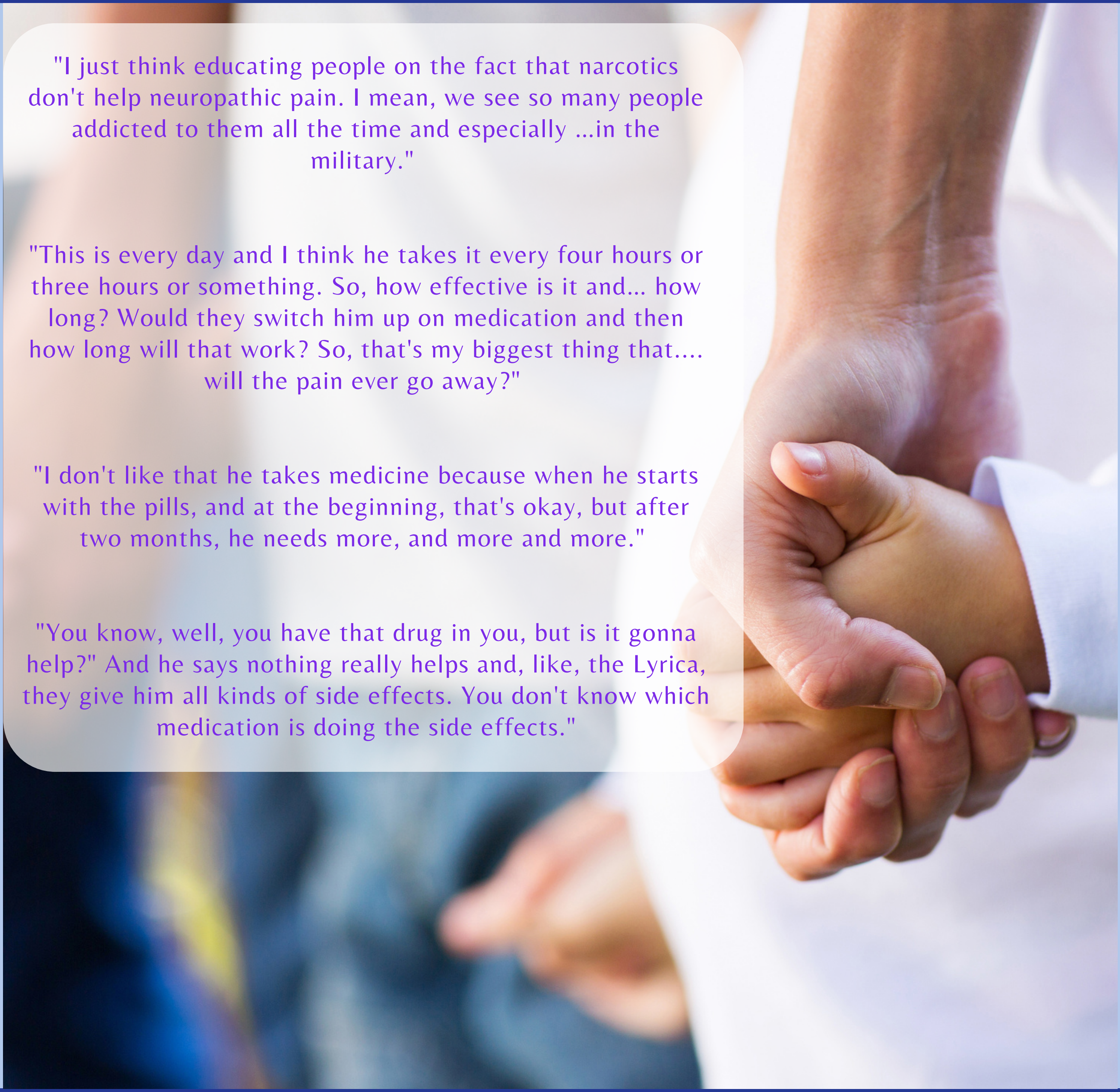

"I just think educating people on the fact that narcotics don't help neuropathic pain. I mean, we see so many people addicted to them all the time and especially ...in the military."

"This is every day and I think he takes it every four hours or three hours or something. So, how effective is it and... how long? Would they switch him up on medication and then how long will that work? So, that's my biggest thing that.... will the pain ever go away?"

"I don't like that he takes medicine because when he starts with the pills, and at the beginning, that's okay, but after two months, he needs more, and more and more."

"You know, well, you have that drug in you, but is it gonna help?" And he says nothing really helps and, like, the Lyrica, they give him all kinds of side effects. You don't know which medication is doing the side effects."

Thoughts about opioids – health care providers

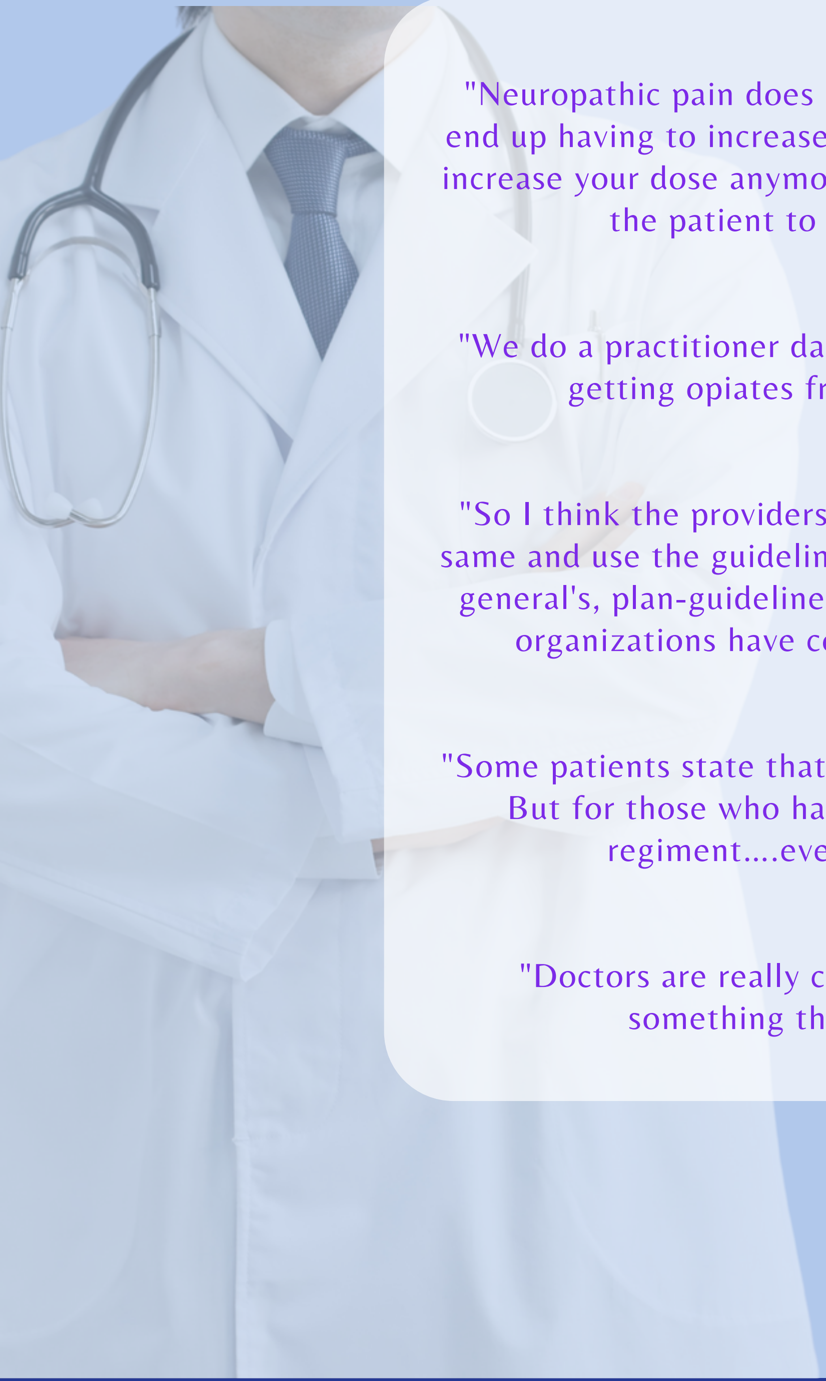

"Neuropathic pain does respond to opiates, for a while. But then you ... end up having to increase your dose and then at a point you don't want to increase your dose anymore and you have to change. You have to convince the patient to change....... and that can be difficult."

"We do a practitioner database query, to look and make sure they're not getting opiates from any outside providers, or the state."

"So I think the providers need to...we need to all to start to practice the same and use the guidelines that are.... coming from the feds, the surgeon general's, plan-guidelines for how we need to manage. And a number of organizations have come out with opiate prescribing guidelines."

"Some patients state that, This is the only medication that seems to work. But for those who have severe pain, ...trying a bunch of different regiment....even the opiates don't necessarily help."

"Doctors are really concerned...they don't want to be accused of something that they thought they were helping."

Thoughts about pain medications - people with SCI

People with SCI have both positive and negative thoughts and experiences regarding the use of pain medication.

Positive

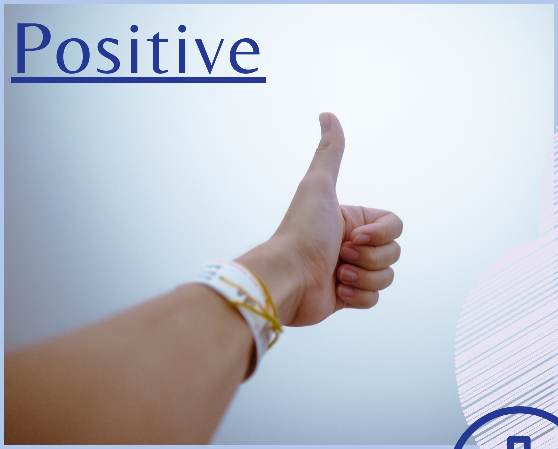

"Lyrica does help me."

"Aleve for me is wonders"

"I take the painkillers when I need to"

"The medicines I use, sometimes work, sometimes don't"

"It doesn't take it all away, but it does help"

"I take different pain medications"

Negative

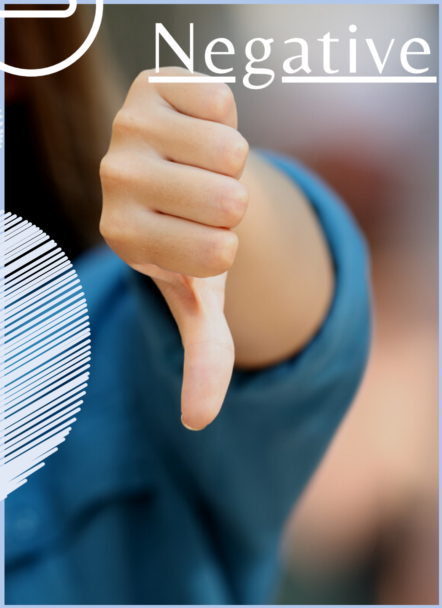

"It didn't really help me."

"The way they tell you to deal with pain is through medication."

"They just throw some drugs at you and.. say, try this and see how you do"

"The doctor that I see at the clinic, he doesn't really talk to me about pain. I tell him what's going on and he usually just throws another pill at me"

Thoughts about pain medications – healthcare providers

Healthcare providers who have extensive experience with SCI patients generally focus on medication, but also encourage combinations with non-pharmacological treatments and self-management.

Treatment

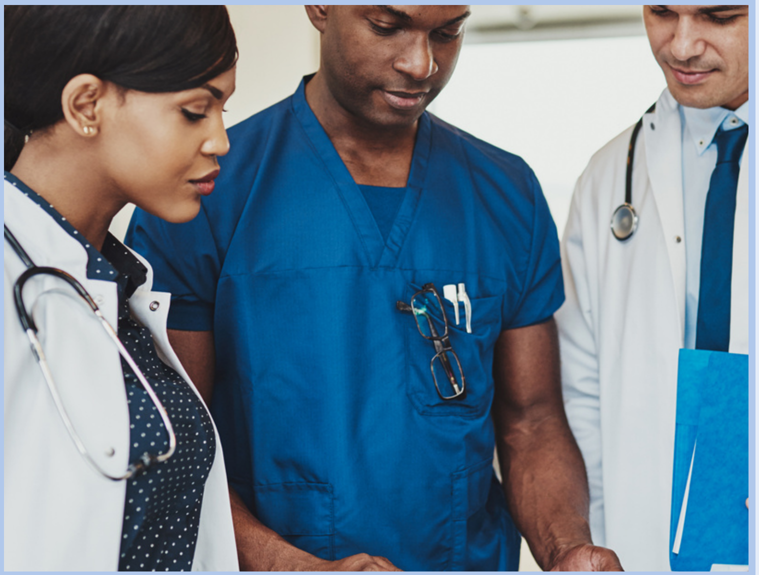

“There's clearly an ability of the medicines to actually reduce pain and .... the physical therapies, and the tens units, all of these things together to reduce pain”

"Because the nerves are smarter than the drugs, tolerance develops over time and so we're increasing doses and then we end up having to switch"

"Gabapentin, Pregabalin, and those are the best in efficacy by far. The anti-depressant class of medications is the next class and they're okay"

"We need more treatment options"

Side Effects

"Every medication will carry a side effect, if not multiple side effects"

"Quite a few patients do not want to be on the medications"

"Find a good balance between medication and side effects can be tough at times"

Normalizing the experience of Anxiety and Depression

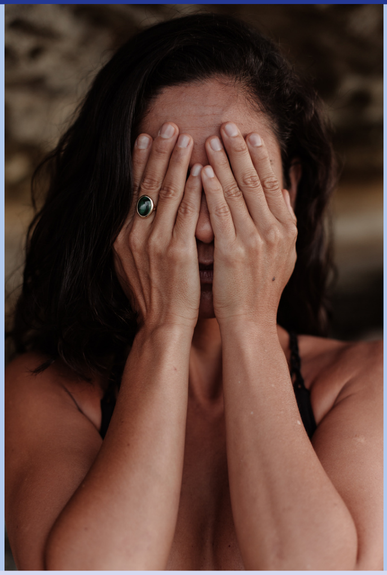
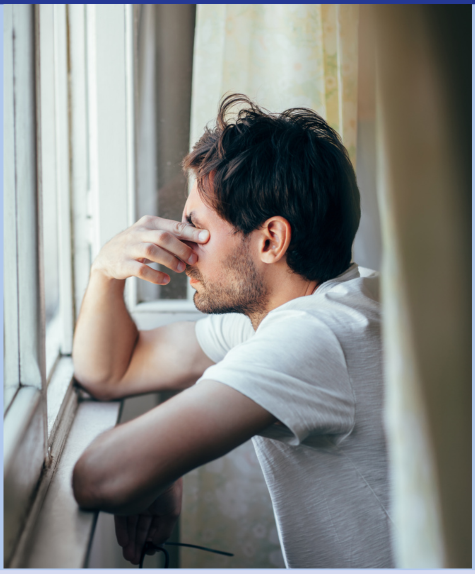
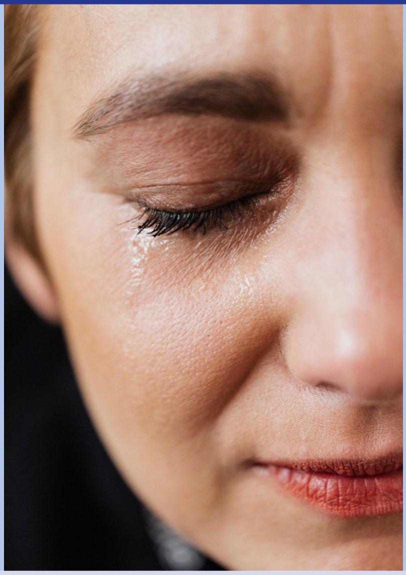

Being told you must learn to live with pain should not be the end of the road, but the beginning. Many individuals living with SCI and chronic pain feel discouraged and experience depression or anxiety because of chronic pain that feels like it is unmanageable. These feelings may also worsen pain. Occasionally, these feelings may even be accompanied by suicidal thoughts as individuals may view ending their lives as the only way of ending their pain and experiencing relief. These feelings are very commonly experienced by individuals who experience chronic pain and disability, and they are also very treatable mental health conditions.

Anxiety and depression are common, and both are highly treatable.Nearly all of us experience some form of anxiety or depression throughout our lifetime, and most clients seen by a mental health professional experiences some form of anxiety or depression.Sometimes people experience anxiety or depression as a side-effect of other clinical issues, such as chronic pain, illness, or loss. Anxiety disorders range from generalized anxiety to post-traumatic stress disorders, among others. Symptoms of anxiety may include, but are not limited to, excessive worrying, irritability, fatigue, problems concentrating, muscle tension, nausea, racing heart, shortness of breath, and/or insomnia. Depression may include symptoms of fatigue, feelings of guilt or worthlessness, loss of interest in things, loss of motivation, loss of sexual drive, changes in appetite, thoughts of suicide, and poor self-esteem.

Recommendations for treating psychological conditions

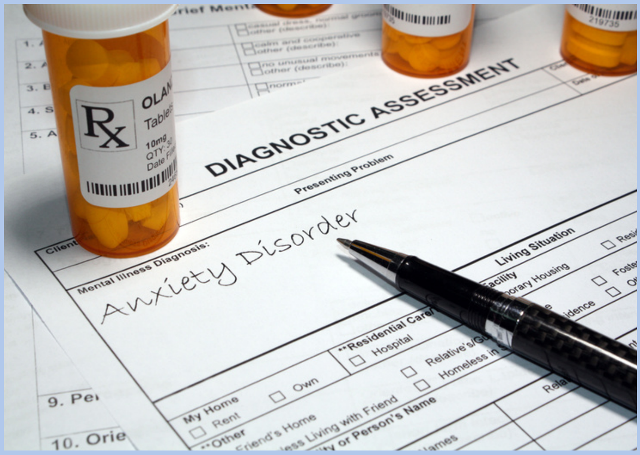

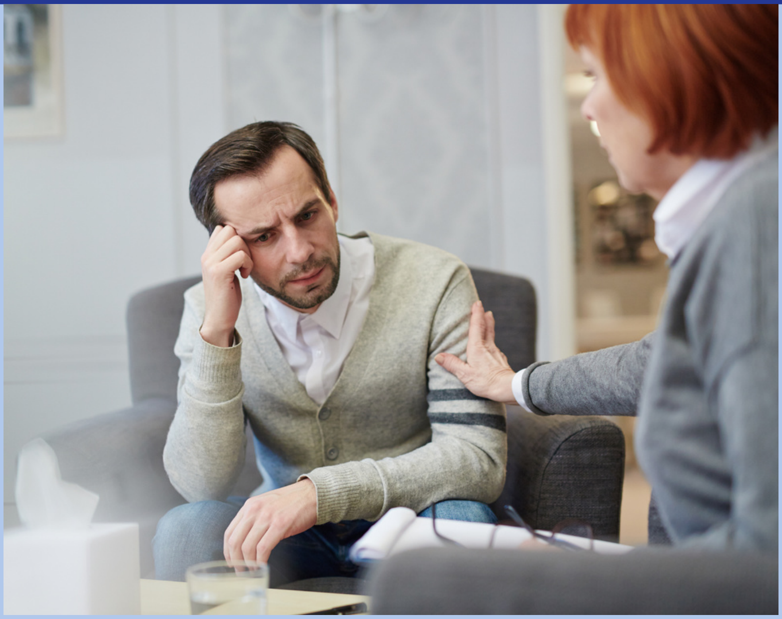

Treating the psychological symptoms of emotional and physical pain is a critical part of your recovery and overall well-being. Psychological conditions can be treated by seeking psychotherapy treatment with a mental health provider. You may learn about cognitive-behavioral strategies to reduce your symptoms of emotional distress, as well as learning about mindfulness strategies that will help you become aware of, and combat, thoughts, and feelings that may serve to increase your pain. Cognitive-behavioral strategies and relaxation skills training can help individuals effectively cope with sensations of physical and emotional distress. With the assistance of a mental health professional, you can learn to develop skills that may help you to effectively manage the psychological side-effects of living with spinal cord injury and chronic pain, which can ultimately result in optimizing your overall quality of life.

In some cases, psychotropic medications are used to treat depression and/or anxiety. These medications may be taken for short periods (i.e. 6 months to 2 years), or for longer periods if needed. The potential benefits of these medications in stabilizing hormonal imbalances that may produce and/or maintain a state of depression and/or anxiety, far outweigh the cost of “taking yet another pill”. You can ask your doctor about how to access these resources.

Some words for significant others, family members/caregivers

It can be challenging to watch a loved one suffer, let alone suffer from chronic pain which can be difficult to soothe and manifests in a variety of ways. Caregivers can be left feeling powerless and stressed. Caregivers are a crucial part of the persons' support system as well as the health system. Taking care of caregivers becomes an important task in taking care of our loved ones suffering from chronic pain. You, the caregiver, are the backbone to your loved one's care as you stay in the background, seldom complain, and juggle a variety of responsibilities. Feelings of helplessness when we see our loved ones in pain can increase our stress response which can lead to symptoms such as diminishing self-esteem, irritability, and loss of focus on your own life. Perhaps you feel that you have less personal time, feel isolated from your own social support system, and experience an increase in worry. What can be done about this array of emotions? The following steps are ways that you, the caregiver, can keep building your resilience, your well-being, and remind yourself that "you can do it!"

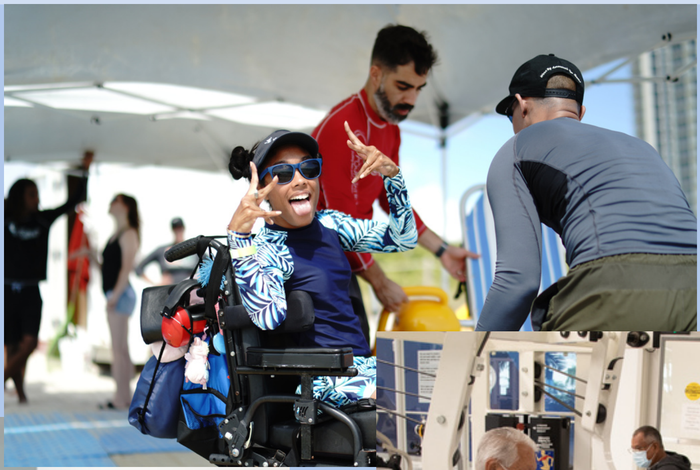

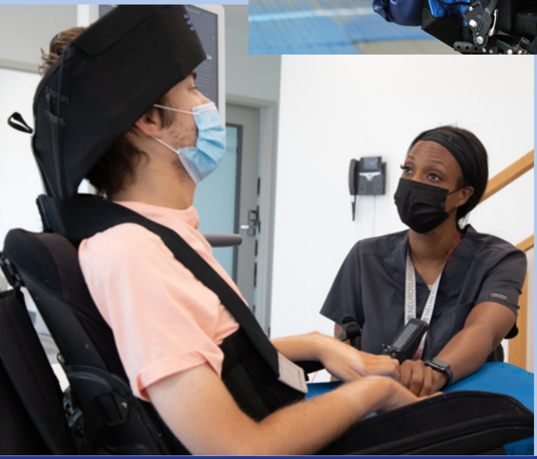

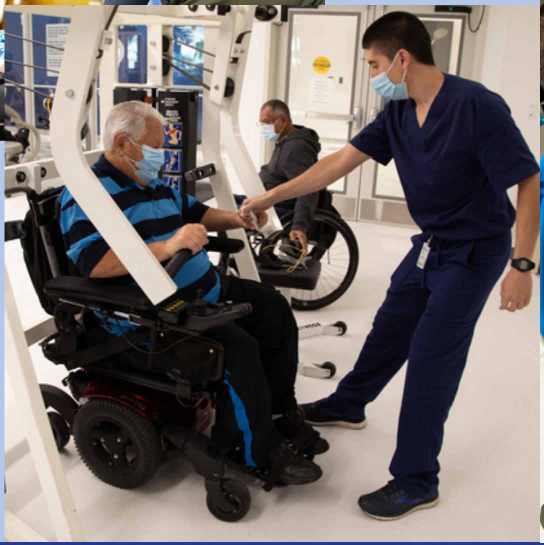

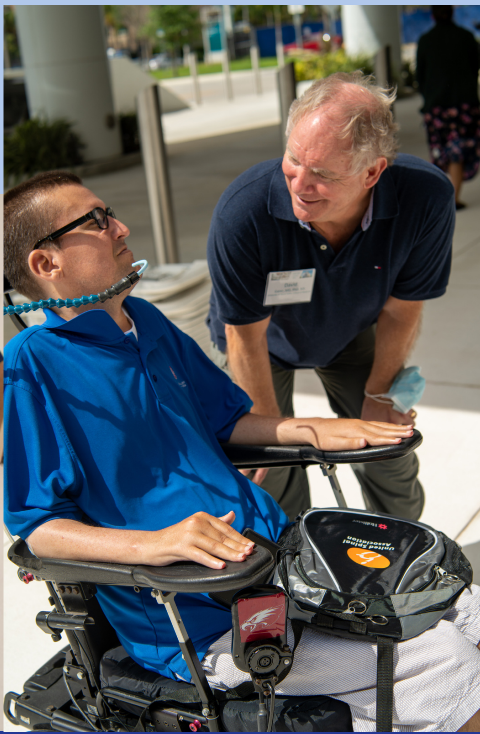

- Ask for help: Help can come in a variety of ways such as a talk with a friend, or a series of talks with a professional (known as psychotherapy), or if finances allow, getting some help to divide your workload. Resources such as support groups are also available to you to help you share your feelings with others who just might be experiencing your exact thoughts and emotions. Family or group psychotherapy is another option available to you and your loved one to help mediate any challenges in your relationship.

- Identify your emotional distress triggers: You can better understand your personal anxiety and/or depression by listing the triggers in your life that make you anxious and/or sad/distressed. Review your list and rate each item from lowest to highest as a source of distress. You can then describe how you physically and emotionally experience distress around these triggers. Being able to predict your triggers and effectively respond to them can help prevent and/or alleviate emotional distress.

- Seek out your social support system: Research has shown that not just high quality, long visits, improve our well-being, but short and frequent visits with others will also lower our stress levels. Reach out to friends, family members, and other individuals who will provide you with positive moments.

- Get out of the house: Arrange to leave your home once a day whether that is to take a walk, go to the grocery store, spend time alone or spend time with friends. Make sure to also obtain sunlight on your skin throughout the day. Research has also shown that sunlight (and vitamin D) has a significant impact on your mood and stress levels. Aim for 30 minutes of sunlight per day and perhaps include your loved one in this adventure if suitable.

- Ground yourself: Learning to meditate, and taking time to do so, can also help re-center you. Perhaps taking 15 minutes each day to meditate, or to simply sit out in your garden and breathe in the fresh air, allowing yourself to be fully present at the moment, can help re-energize you. Try and make this a daily practice.

- Breath Work: Working with the breath is one of the simplest yet most powerful forms of relaxation training. Practicing abdominal or diaphragmatic breathing (taking slow deep breaths) can increase the amount of oxygen in your body which enhances energy production, strengthens the immune system, quiets the mind, improves mental concentration, and reduces anxiety and muscle tension.

- Keep a gratitude journal: Make a habit of writing 3 things you are grateful for each day. It may be as simple as spotting a bird in your garden or enjoying a cup of coffee. Research has shown that focusing on at least 3 things each day that we can be grateful for helps to improve mood.

- Positive Affirmations: Affirmations are self-messages that affirm ideas. We send ourselves these messages all day long, sometimes unconsciously. Being able to identify these messages and determine if they are helpful or not to us can be valuable. Sending ourselves negative messages will raise our anxiety, yet sending positive messages or affirmations, such as "I know this will be challenging, but I can handle it," can serve to reduce our anxiety.
- Be Mindful of each moment: Allow yourself to not become overwhelmed by thoughts of the future, or by past events, but rather stay focused on the here and now. This will help reduce unnecessary worries that get created by living in the imagined future and will help you see that if you stay present in the moment, what you are feeling in this moment will pass. Remember that the only permanence in life is change itself.

While you may feel sad, stressed, or helpless, you, the caregiver, are the essential support for your loved one and, therefore, are the very individual that also requires care. During this time, remember to care for yourself by implementing these strategies, as well as any other positive.

Remember also that the techniques mentioned above have different degrees of effectiveness for different people, and it is important to experiment with each in order to find which technique works the best for you. Therefore, being patient and practicing the chosen techniques on a regular basis over time is key.

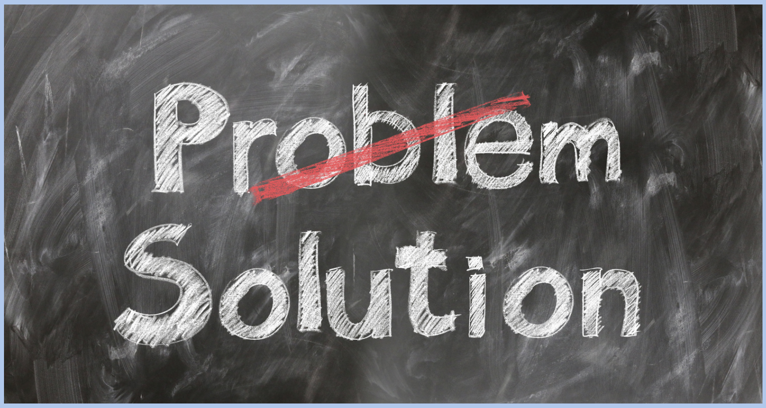

Final Message

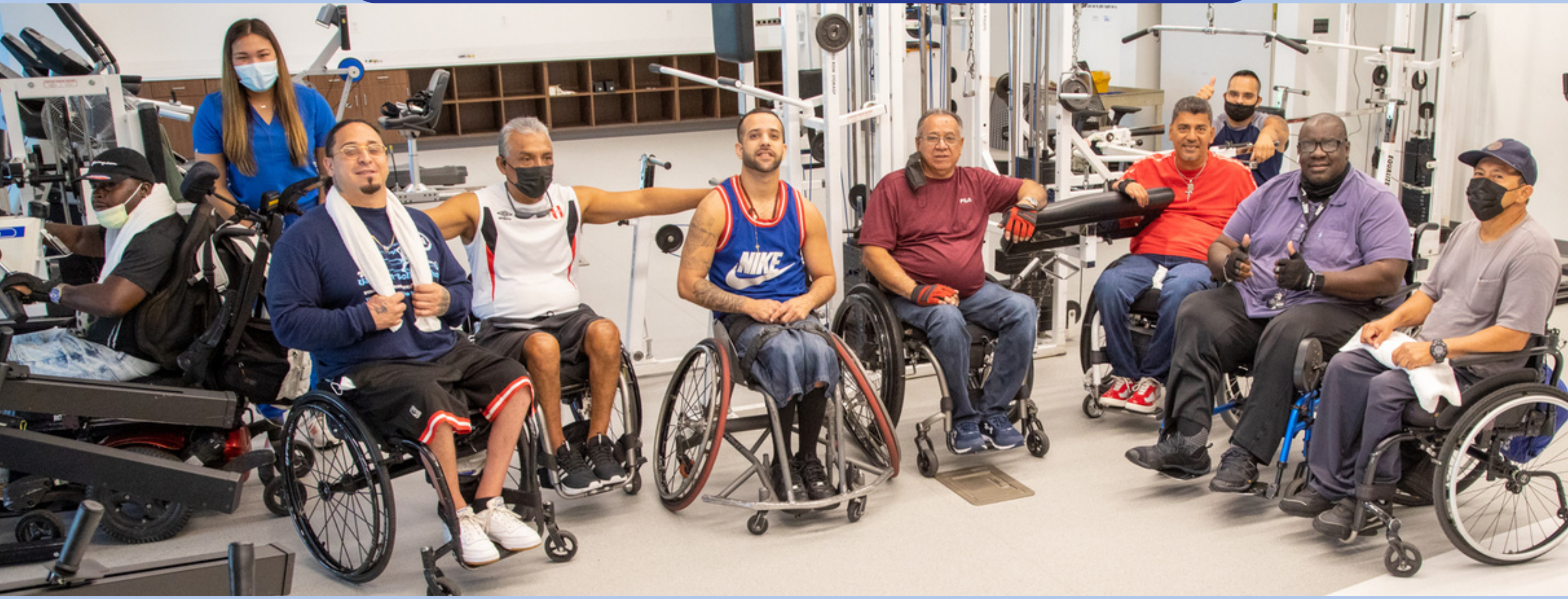

"Like I said you have to do what works for you. Take in the knowledge and then do what works for you."

But remember that...

YOU ARE NOT ALONE!

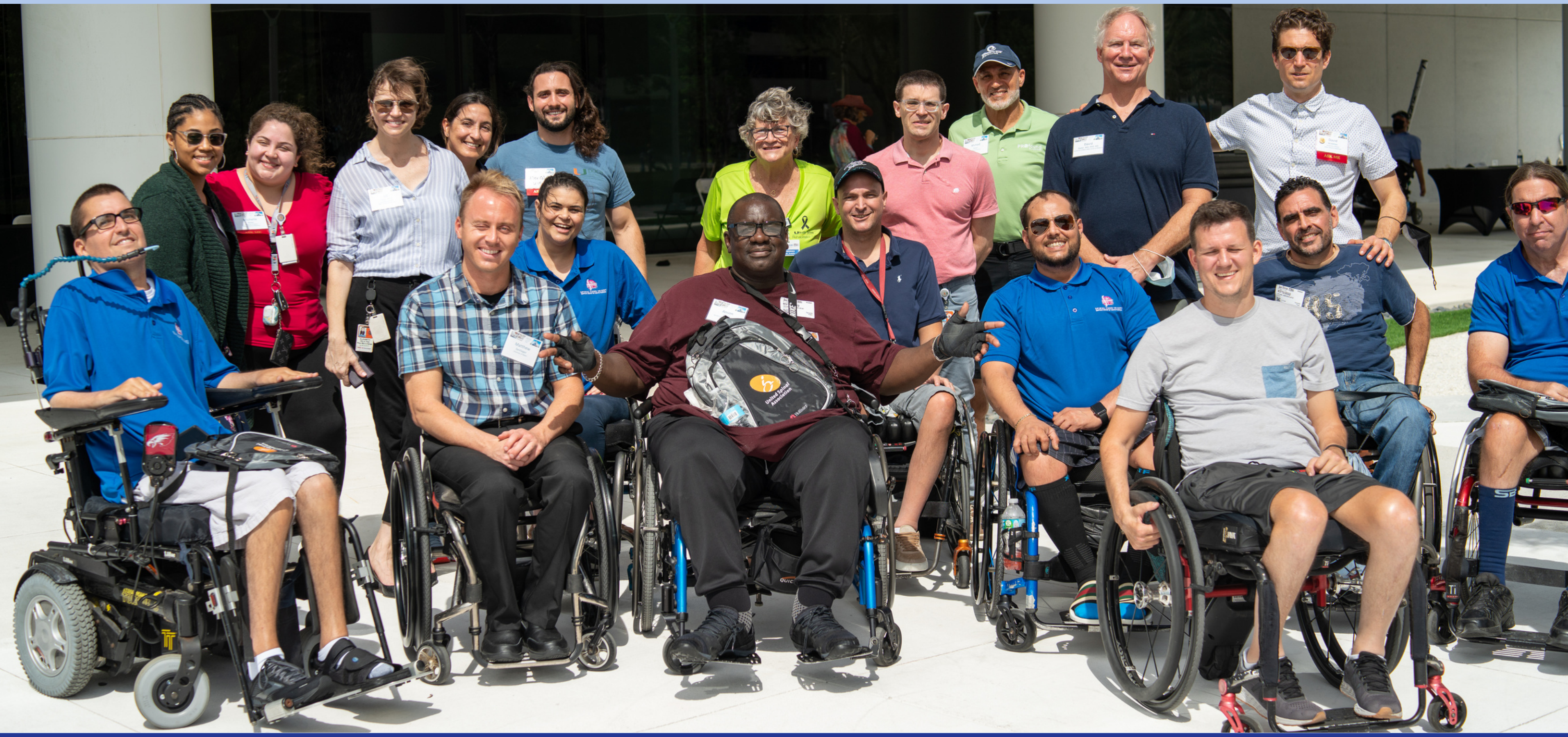

NOTES ABOUT MY PAIN

WHAT FACTORS TRIGGER/INCREASE MY PAIN?

WHAT RELIEVES MY PAIN?

WHAT TYPE OF TREATMENT WOULD I LIKE TO TRY?

Resources

Pain Management Network – An excellent guide to understanding your pain featuring videos and explanations from people with SCI and pain at https://painmanagement.network/

Model Systems Knowledge Translation Center - SCI Pain – a reliable and easy to understand brief guide to understanding and managing pain after SCI at https://msktc.org/sci

Scientific articles:

- "Cellular and Molecular Mechanisms of Pain" by Basbaum, et al. 2009: https://www.ncbi.nlm.nih.gov/pmc/articles/PMC2852643/?tool=pmcentrez. A free, comprehensive article on the biology of pain.
- "Central sensitization: implications for the diagnosis and treatment of pain." https://www.ncbi.nlm.nih.gov/pubmed/20961685
- "Neuronal-Glial Interactions Maintain Chronic Neuropathic Pain after Spinal Cord Injury" https://www.ncbi.nlm.nih.gov/pmc/articles/PMC5603132/. A free review article on the biology of neuropathic pain after SCI.
- Opioid Basics https://www.cdc.gov/drugoverdose/opioids/index.html
- Understanding the Epidemic https://www.cdc.gov/drugoverdose/epidemic/index.html
- What is the U.S. Opioid Epidemic https://www.hhs.gov/opioids/about-the-epidemic/index.html
- Cheatle, MD. (2018). Balancing the Risks and Benefits of Opioid Therapy for Patients with Chronic Nonmalignant Pain: Have We Gone Too Far or Not Far Enough? Pain Medicine. 19:642–645. https://doi.org/10.1093/pm/pny037
- Gallagher RM, Rosenthal LJ. (2008). Chronic Pain and Opiates: Balancing Pain Control and Risks in Long-Term Opioid Treatment. Arch Phys Med Rehabil. 89:S77-82. doi: 10.1016/j.apmr.2007.12.003.

Phone Apps

- Mindfulness Coach: https://mobile.va.gov/app/mindfulness-coach
- Calm: https:/www.calm.com/
- Headspace: https://www.headspace.com/

PDFs and Websites for pain management:

- Mindfulness: https://www.aci.health.nsw.gov.au/__data/assets/pdf_file/0005/212864/Mindfulness_1.pdf
- Imagery: https://www.aci.health.nsw.gov.au/__data/assets/pdf_file/0004/212863/Imagery_1.pdf
- Progressive muscle relaxation: https://www.aci.health.nsw.gov.au/__data/assets/pdf_file/0003/212862/Progressive_Muscle_Relaxation_2.pdf
- Slow Focused Breathing: https://www.aci.health.nsw.gov.au/__data/assets/pdf_file/0006/212865/Slow_Focused_Breathing_1.pdf
- Better Pain Management https://www.hhs.gov/opioids/about-the-epidemic/hhs-response/better-pain-management/index.html

Wordlist with definitions

- Allodynia: When a stimulus that is usually not painful triggers pain.
- At-level neuropathic pain: Pain that occurs around the level of injury and starts early after the spinal cord injury.
- Below-level neuropathic pain: Pain that occurs below the level of injury and usually develops over the first year of injury.
- Central nervous system: A part of the nervous system that merges information from our senses and coordinates all body activities.
- Central sensitization: State in which neurons that carry pain signals become very sensitive (easily activated).
- Chronic pain: Persistent or intermittent pain that lasts for more than three months.
- Complete SCI: Total loss of sensory and motor function below the level of injury.
- Dermatome: Skin area innervated by specific spinal nerves.
- Evidence-based treatment: Treatment that has been shown, in research studies, to be more effective than a placebo.
- Hyperalgesia: When a stimulus that usually triggers mild pain produces severe pain.
- Incomplete SCI: Some sensation and/or movement is present below the level of injury.
- Injury level: Refers to where along the spinal cord the injury occurred.
- Microglial activation: State in which microglia becomes active and releases inflammatory factors.
- Nerve: A bundle of fibers that uses electrical and chemical signals to transmit sensory and motor information from one body part to another.
- Neurological Level of injury: The most caudal spinal cord segment with both normal sensory and motor function.
- Neuropathic pain: Pain associated with a lesion or disease affecting the brain, spinal cord, or peripheral nerves.
- Neuroplasticity: The ability of the nervous system to reorganize in response to learning or the following injury.
- Nociceptive musculoskeletal pain: Pain associated with the activation of nociceptors in muscles, joints, and bones.
- Nociceptive pain: Pain caused by the activation of nociceptors.
- Nociceptive visceral pain: Pain caused by the activation of nociceptors in internal organs.
- Nociceptors: Pain sensors located throughout the body.
- Opioid: A substance that acts on opioid receptors to produce pain relief.
- Pain: An unpleasant sensory and emotional experience from actual or potential tissue damage.
- Placebo: A substance or treatment that is designed to have no therapeutic effects on the patient.
- Spinal cord injury: A physical damage to the spinal cord which can alter normal motor, sensory or autonomic function.

References

- Classification of chronic pain: descriptions of chronic pain syndromes and definitions of pain terms. (IASP Press, 1994).
- Banks, S. M. & Kerns, R. D. Explaining high rates of depression in chronic pain: A diathesis-stress framework. Psychol. Bull. (1996). doi:10.1037/0033-2909.119.1.95
- Hammell, K. W., Miller, W. C., Forwell, S. J., Forman, B. E. & Jacobsen, B. A. Fatigue and spinal cord injury: a qualitative analysis. Spinal Cord 47, 44–49 (2009).
- Nicholson Perry, K., Nicholas, M. K. & Middleton, J. Spinal cord injury-related pain in rehabilitation: a cross-sectional study of relationships with cognitions, mood and physical function. Eur. J. Pain Lond. Engl. 13, 511–517 (2009).
- Apkarian, A. V., Bushnell, M. C., Treede, R.-D. & Zubieta, J.-K. Human brain mechanisms of pain perception and regulation in health and disease. Eur. J. Pain Lond. Engl. 9, 463–484 (2005).
- Derbyshire, S. W. et al. Pain processing during three levels of noxious stimulation produces differential patterns of central activity. Pain 73, 431–445 (1997).
- Baliki, M. N. et al. Chronic pain and the emotional brain: specific brain activity associated with spontaneous fluctuations of intensity of chronic back pain. J. Neurosci. Off. J. Soc. Neurosci. 26, 12165–12173 (2006).
- Baliki, M. N., Geha, P. Y., Apkarian, A. V. & Chialvo, D. R. Beyond feeling: chronic pain hurts the brain, disrupting the default-mode network dynamics. J. Neurosci. Off. J. Soc. Neurosci. 28, 1398–1403 (2008).
- Apkarian, A. V. et al. Chronic back pain is associated with decreased prefrontal and thalamic gray matter density. J. Neurosci. Off. J. Soc. Neurosci. 24, 10410–10415 (2004).
- Li, X.-Y. et al. Alleviating Neuropathic Pain Hypersensitivity by Inhibiting PKMζ in the Anterior Cingulate Cortex. Science 330, 1400–1404 (2010).
- Apkarian, A. V. et al. Chronic pain patients are impaired on an emotional decision-making task. Pain 108, 129–136 (2004).
- Dick, B. D. & Rashiq, S. Disruption of attention and working memory traces in individuals with chronic pain. Anesth. Analg. 104, 1223–1229, tables of contents (2007).
- Siddall, P., McClelland, J., Rutkowski, S. & Cousins, M. A longitudinal study of the prevalence and characteristics of pain in the first 5 years following spinal cord injury. Pain 103, 249–257 (2003).
- Finnerup, N. B. et al. Phenotypes and Predictors of Pain Following Traumatic Spinal Cord Injury: A Prospective Study. J. Pain 15, 40–48 (2014).
- Widerström-Noga, E. G., Felipe-Cuervo, E. & Yezierski, R. P. Chronic pain after spinal injury: Interference with sleep and daily activities. Arch. Phys. Med. Rehabil. 82, 1571–1577 (2001).
- Amsters, D. et al. Factors Which Facilitate or Impede Interpersonal Interactions and Relationships after Spinal Cord Injury: A Scoping Review with Suggestions for Rehabilitation. Rehabilitation Research and Practice (2016). doi:10.1155/2016/9373786
- NHS. (n.d.). Duloxetine. NHS choices. Retrieved from https://www.nhs.uk/medicines/duloxetine/.
